# I_2_/DMSO-Catalyzed Transformation of *N*-tosylhydrazones to 1,2,3-thiadiazoles

**DOI:** 10.3389/fchem.2020.00466

**Published:** 2020-06-12

**Authors:** Weiwei Li, Jun Zhang, Jing He, Liang Xu, Luigi Vaccaro, Ping Liu, Yanlong Gu

**Affiliations:** ^1^The Key Laboratory for Green Processing of Chemical Engineering of Xinjiang Bingtuan, School of Chemistry and Chemical Engineering, Shihezi University, Shihezi, China; ^2^Key Laboratory of Material Chemistry for Energy Conversion and Storage, Ministry of Education, Hubei Key Laboratory of Material Chemistry and Service Failure, School of Chemistry and Chemical Engineering, Huazhong University of Science and Technology, Wuhan, China; ^3^Laboratory of Green S.O.C., Dipartimento di Chimica, Biologia e Biotecnologie, Università degli Studi di Perugia, Perugia, Italy

**Keywords:** *N*-tosylhydrazone, 1,2,3-thiadiazoles, iodine, DMSO, sulfur

## Abstract

An iodine/DMSO catalyzed selective cyclization of *N*-tosylhydrazones with sulfur without adding external oxidant was developed for the synthesis of 4-aryl-1,2,3-thiadiazoles. In this reaction, oxidation of HI by using DMSO as dual oxidant and solvent is the key, which allowed the regeneration of I_2_, ensuring thus the success of the synthesis. This protocol features by simple operation, high step-economy (one-pot fashion), broad substrate scope as well as scale-up ability.

## Introduction

**Figure 1:**
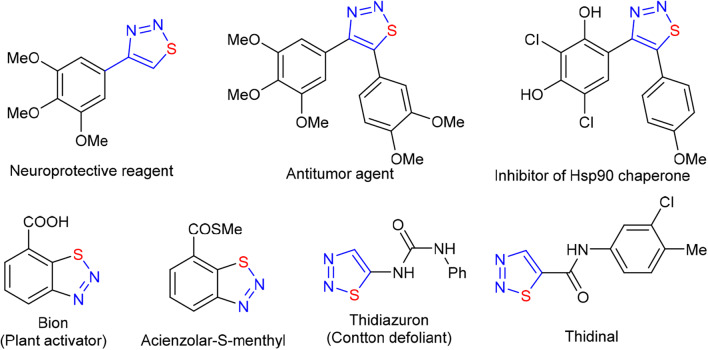
Bioactive molecules with a 1,2,3-thiadiazole moiety.

1,2,3-Thiadiazole, as an important 2N1S-heterocyclic structural unit, is ubiquitous in natural products and drug molecules (Figure 1; Bakulev and Dehaen, 2004; Shafran et al., 2018). Because of their unique biological activity and intrinsic reactivity, 1,2,3-thiadiazoles were widely used in medicine (Mloston and Huisgen, 1985, 1989; Thomas et al., 1985; Huisgen and Mloston, 1999; Wu et al., 2007; Cikotiene et al., 2009; Atta et al., 2010; Dong et al., 2010; Amirhamzeh et al., 2013), pesticides (Jalilian et al., 2003; Li et al., 2005; Fan et al., 2009; Wang et al., 2009; Zheng et al., 2010) and organic synthesis (Förster et al., 1997; Takimiya et al., 1997; Androsov and Neckers, 2007; Androsov, 2008; Teplyakov et al., 2013). In the past two decades, many efforts have been made to construct the 1,2,3-thiadiazole skeleton. The reported methods can be cataloged as the followings: (a) the 1,3-dipolar cycloaddition of diazoalkanes to thiocarbonyl compounds (Pechmann and Nold, 1896; Sheehan and Izzo, 1949; Martin and Mucke, 1965; Capuano et al., 1983; Aoyama et al., 1986); (b) Hurd-Mori synthesis and the analogous processes (Hurd and Mori, 1955; Kumar et al., 2012; Mo et al., 2019; Zhang et al., 2019); (c) the cyclization of Lawesson reagent with diazotized α-aminoketone (Caron, 1986); and (d) the [3 + 2] cycloaddition of α-enolic dithioester with tosyl azide (Singh et al., 2013). Although these methods provided some promising routes to access 1,2,3-thiadiazoles, the reported protocols also plagued by some drawbacks, such as the use of highly reactive reagents or pre-functionalized substrates, harsh reaction conditions, and a limited scope of substrate. Therefore, an effective route to construct 1,2,3-thiadiazole skeleton by using readily available chemicals is appealingly needed.

**Scheme 1:**
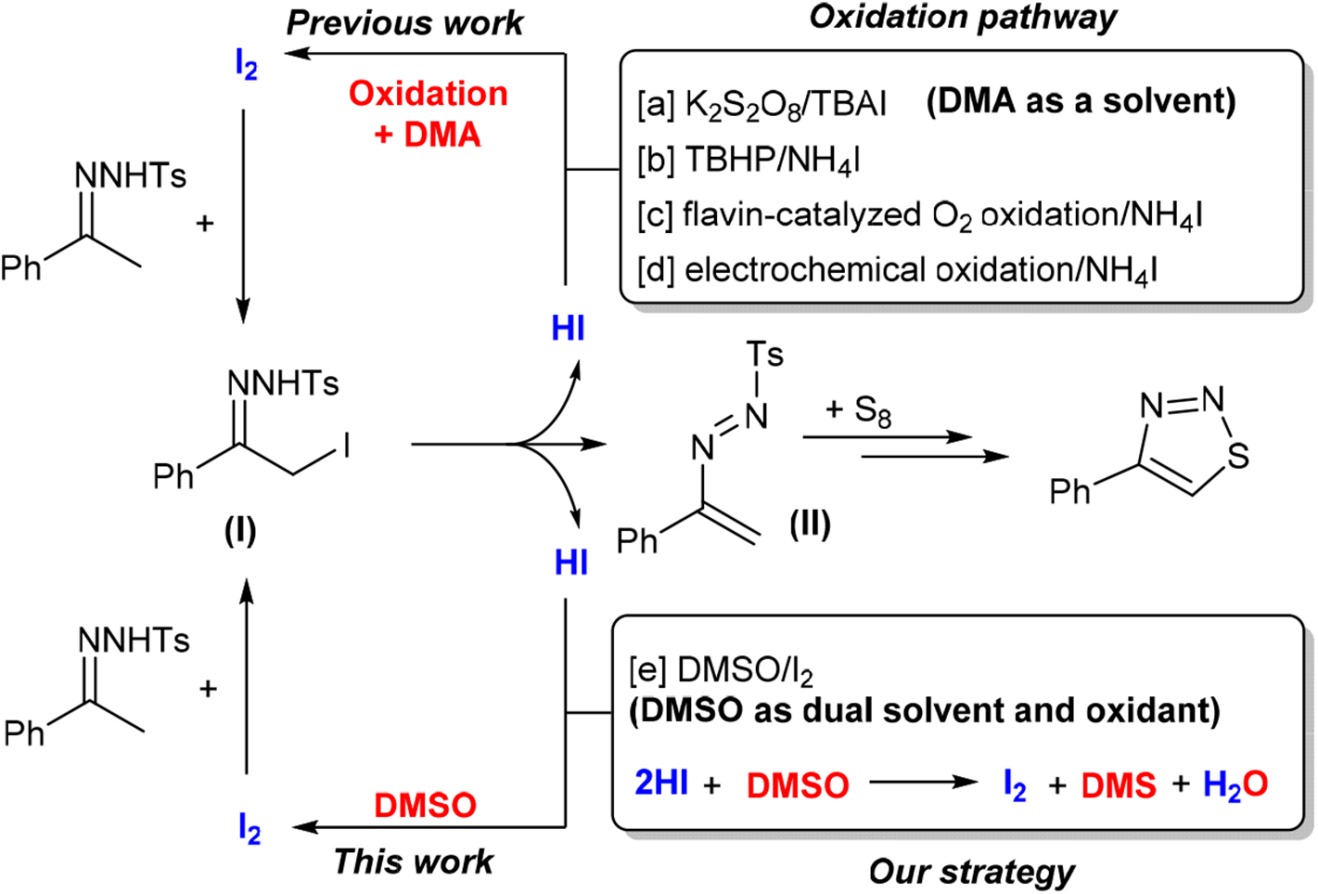
Iodine-catalyzed cyclization of *N*-tosylhydrazone with S_8_.

Recently, *N*-tosylhydrazones, which are readily accessible and inexpensive chemicals, have attracted much attention in the construction of heterocyclic compounds (Xia and Wang, 2017). In particular, iodine-catalyzed cyclization of *N*-tosylhydrazone with elemental sulfur has become one of the most efficient methods to synthesize 4-aryl-1,2,3-thiadiazoles (Chen et al., 2015; Ishikawa et al., 2017; Liu et al., 2018; Li et al., 2019). This transformation was triggered by α-iodation of acetophenone tosylhydrazone, which was generated *in situ* from the corresponding precursors (Scheme 1). One molecule of hydrogen iodide (HI) was also formed at the same time. To avoid the detrimental effect of acidic HI, and also to facilitate progress of a catalytic reaction, in the previous reports, an oxidizing reagent was adopted to convert HI to I_2_. With this strategy, some effective systems, such as K_2_S_2_O_8_/TBAI (Scheme 1, method a; Chen et al., 2015), TBHP/NH_4_I (method b; Li et al., 2019), and flavin-catalyzed O_2_ oxidation/NH_4_I (method c; Liu et al., 2018), have been developed successfully. Electrochemical oxidation in the combination of using NH_4_I as additive was also proved to be effective (method d; Ishikawa et al., 2017). Although the reported synthesis of 4-aryl-1,2,3-thiadiazoles through cyclization of *N*-tosylhydrazones is promising, owing to the addition of a large amount of oxidizing reagent or the use of special equipment, the effectiveness and the greenness of the synthesis were negatively affected by a time-consuming product separation procedure and the generation of waste.

Very recently, Wu et al. reported an efficient I_2_/CuCl_2_-promoted one-pot three-component strategy for the construction of 1,2,3-thiadiazoles from aliphatic- or aromatic-substituted methyl ketones, *p*-toluenesulfonyl hydrazide, and potassium thiocyanate in the presence of DMSO as solvent (Wang et al., 2019). However, excess stoichiometric amounts of I_2_ and CuCl_2_ are required. On the other hand, the combination of I_2_ and DMSO emerged recently as an effective and eco-friendly oxidative system for organic synthesis (Saba et al., 2015, 2016; Rafique et al., 2016; Silva et al., 2017; Monga et al., 2018). The past decade already witnessed the powerful productivity of this unique system to strengthen atom- and step-economic organic synthesis. Particularly, under appropriate conditions, regeneration of I_2_ from HI with the aid of DMSO proven to be practically feasible (Kalmode et al., 2014; Wu et al., 2014; Deshidi et al., 2015; Mohammed et al., 2015; Huang et al., 2019; Li et al., 2019). This not only minimized the dosage of I_2_ but also enabled us to establish some new reactions without adding external auxiliary reagent, simplifying thus the reaction system. Based on this observation, we envisaged that the I_2_/DMSO system may be applicable in the cyclization of *N*-tosylhydrazone with elemental sulfur. The dual role of DMSO as both solvent and oxidant, if it works, will allow us to synthesize 1,2,3-thiadiazoles in a simple system. Our preliminary results show that our speculation is indeed reasonable. Herein, we wish to report a facile synthesis of 4-aryl-1,2,3-thiadiazoles via an I_2_-catalyzed reaction between *N*-tosylhydrazones and element sulfur in DMSO solvent (Scheme 1, method e).

## Results and Discussion

Our study commenced from a reaction of *N*-tosylhydrazone **1a** and sulfur (S_8_). Initially, 0.30 mmol of **1a** was mixed with 0.90 mmol of S_8_. The reaction was performed in DMSO, and the obtained results are listed in Table 1. No reaction occurred after 5 h of heating under air at 100°C (entry 1). Addition of 20 mol% of KI or tetrabutylammonium iodide (TBAI) cannot initiate the reaction either (entries 2 and 3). This is quite reasonable because, under non-acidic conditions, it is difficult to oxidize the iodide anion to elemental iodine by DMSO. Ammonium iodide (NH_4_I) has stronger acidity compared with that of TBAI. By using NH_4_I as a catalyst, the reaction proceeded slowly. And after 5 h, the desired product, 4-phenyl-1,2,3-thiadiazole **3a**, was obtained in 11% of yield (entry 4). Intriguingly, I_2_ can catalyze the cyclization reaction effectively in conjunction with using DMSO as solvent, and the reaction yield reached 79% (entry 5). The choice of solvent is crucial. When DMSO was replaced by the other organic solvents, such as toluene, DMF, and 1,4-dioxane, the reaction proceeded hardly (entries 6–8). In an alcoholic solvent, isopropanol (IPA), the reaction can be initiated, but it proceeded very slowly. As a result, the yield stopped only at 15% (entry 9). To further improve the reaction yield, the effect of the dosage of I_2_ was scrutinized. Interestingly, it was found that the reaction yield could be improved to 86% by decreasing the amount of I_2_ to 10 mol% (entry 10). However, a further decrease of the I_2_ loading resulted in a drastic loss of the reaction yield (entry 11). With 2.5 mol% of I_2_, **3a** can be isolated only in 10% yield (entry 12). We also tested the model reaction under argon. In this case, the reaction proceeded smoothly, and **3a** can be isolated in 90% yield (entry 13). The yield can be slightly improved by decreasing the amount of S_8_ to 2.0 equivalents (92%, entry 14). From the viewpoint of green chemistry, the best system should allow also the use of an equal amount of precursors. Unfortunately, when **1a** and **2a** were charged equally, the reaction proceeded sluggishly (entry 15). Also, adjustment of the reaction temperature, reaction time, and the amount of DMSO did not significantly promote the reaction (entries 16–21). And finally, to reach a compromise of all of the reaction parameters, the optimal conditions were confirmed to be I_2_ catalyst (10 mol%), DMSO solvent, the ratio of **1a**/**2a** is 1/2. The performance of NBS or NIS, which has also been used as an oxidizing reagent (Huang et al., 2017; Gu et al., 2018a,b; Xu et al., 2019), was also examined under the optimal conditions. However, only a trace amount of **3a** can be detected (entries 22 and 23). This result demonstrated that to take the oxidizing ability of DMSO as a means to implement the synthesis, the use of I_2_ is mandatory.

**Table 1:** Optimization of reaction conditions^a^.

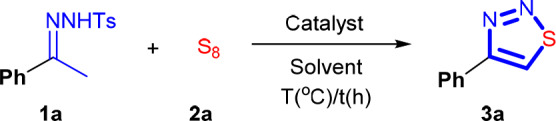					
**Entry**	**Catalyst**	**Solvent**	**T/°C**	**t/h**	**Yield(%)^b^**
1	—	DMSO	100	5	0
2	KI (20 mol%)	DMSO	100	5	Trace
3	TBAI (20 mol%)	DMSO	100	5	Trace
4	NH_4_I (20 mol%)	DMSO	100	5	11
5	I_2_ (20 mol%)	DMSO	100	5	79
6	I_2_ (20 mol%)	Toluene	100	5	Trace
7	I_2_ (20 mol%)	DMF	100	5	Trace
8	I_2_ (20 mol%)	1,4-Dioxane	100	5	Trace
9	I_2_ (20 mol%)	IPA	100	5	15
10	I_2_ (10 mol%)	DMSO	100	5	86
11	I_2_ (5.0 mol%)	DMSO	100	5	52
12	I_2_ (2.5 mol%)	DMSO	100	5	10
13	I_2_ (10 mol%)	DMSO	100	5	90
14^c^	I_2_ (10 mol%)	DMSO	100	5	92
15^d^	I_2_ (10 mol%)	DMSO	100	5	58
16	I_2_ (10 mol%)	DMSO	80	5	86
17	I_2_ (10 mol%)	DMSO	120	5	59
18	I_2_ (10 mol%)	DMSO	100	2	78
19	I_2_ (10 mol%)	DMSO	100	10	75
20	I_2_ (10 mol%)	DMSO(1 mL)	100	5	72
21	I_2_ (10 mol%)	DMSO(0.5 mL) + DMA(2.0 mL)	100	5	68
22	NBS (10 mol%)	DMSO	100	10	Trace
23	NIS (10 mol%)	DMSO	100	10	Trace

a *Reaction conditions: **1a** (0.30 mmol), **2a** (0.90 mmol), solvent (3 mL), under air (entries 1–12); under argon (entries 13–23)*.

b *Isolated yield*.

c *0.60 mmol of **2a** was added*.

d *0.30 mmol of **2a** was added*.

**Scheme 2:**
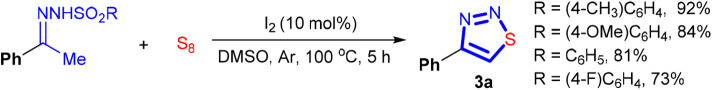
Effect of arylsulfonyl groups on yield of **3a**.

With the optimized reaction conditions in hand, we then explored the effect of the arylsulfonyl group in the sulfonylhydrazone component on the reaction. As shown in Scheme 2, all the examined *N*-arylsulfonylhydrazones reacted with sulfur readily. Electron-rich *N*-arylsulfonylhydrazones seemingly like favorable for producing **3a**. For example, while 81% of yield was obtained with an *N*-phenylsulfonylhydrazone formed from PhSO_2_NHNH_2_, the electron-deficient congener from 4-F-C_6_H_4_SO_2_NHNH_2_ gave **3a** in 73% yield. Similarly, the reactions with electron-rich *N*-arylsulfonylhydrazones, like *N*-tosylhydrazone or its analogous with a methoxy group formed from 4-OMe-C_6_H_4_SO_2_NHNH_2_, provided a slightly higher yield of **3a** than the former two (92 and 84%).

**Scheme 3:**
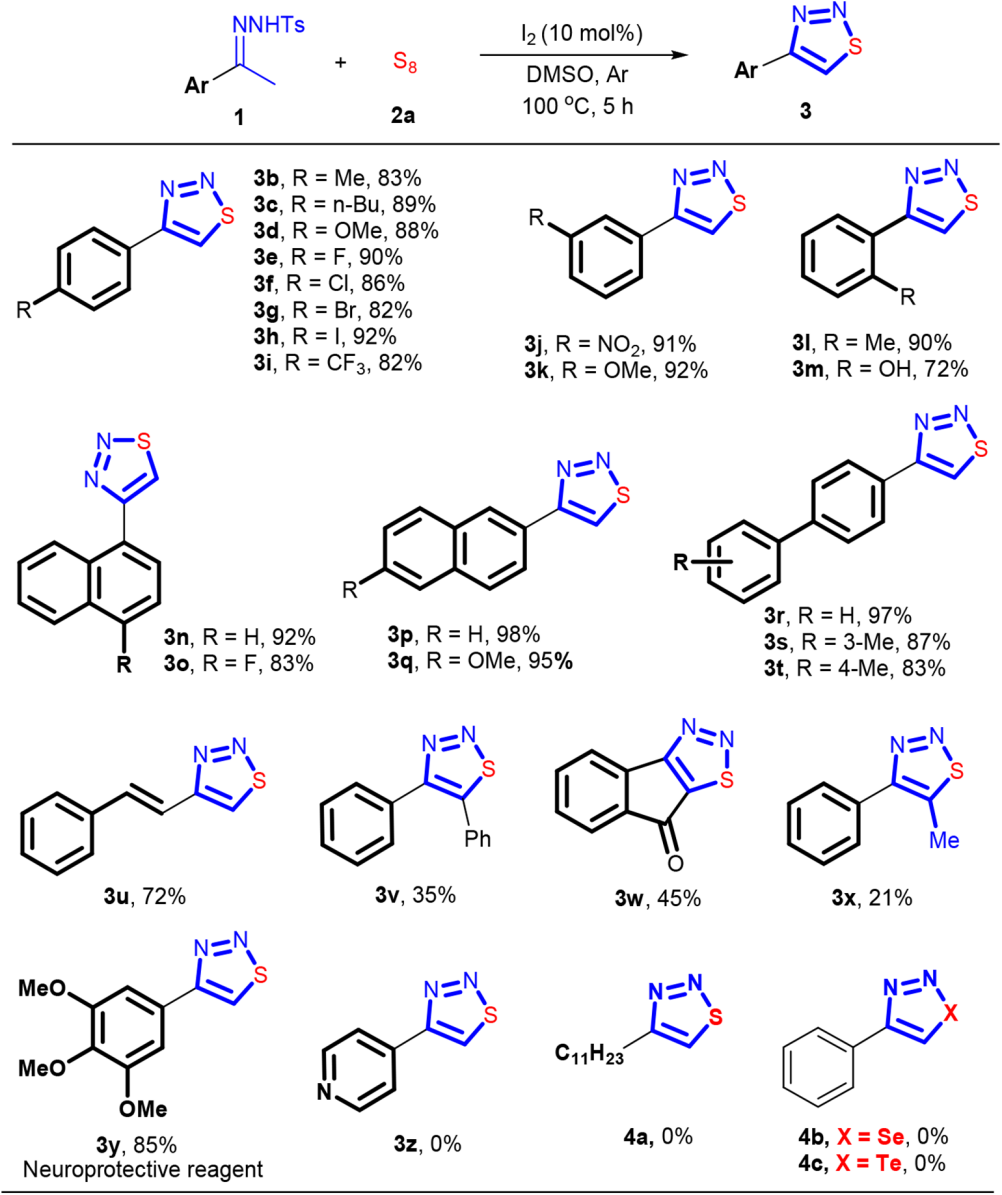
The substrate scope of *N*-tosylhydrazones. Reaction conditions: 0.3 mmol **1**, 0.6 mmol S_8_, I_2_ (10 mol%), DMSO (3 mL), 5 h, under argon. Isolated yield.

The effect of the imine part in the *N*-tosylhydrazone component on the reaction was also investigated, and the results are shown in Scheme 3. *N*-tosylhydrazones with different functional groups on the arene ring of the imine part all worked well under the standard conditions, efficiently providing the corresponding 4-aryl-1,2,3-thiadiazoles **3b−3i** with yields ranging from 82 to 92%. The electronic nature of the substituents on the arene ring of the imine part, involving electron-donating (4-Me, 4-nBu, and 4-MeO) and electron-withdrawing (4-F, 4-Cl, 4-Br, 4-I, and 4-CF_3_) groups, had no obvious effect on the yields. Further, substituents in the *meta* position of the arene ring also showed good compatibility, giving cyclization products **3j** and **3k** in 91 and 92% yield, respectively. The *N*-tosylhydrazones with an *ortho*-substituted arene in their imine parts were also applicable in this reaction. For example, **3l** and **3m** can be synthesized in 90 and 72% yield, respectively. More sterically demanding *N*-tosylhydrazones derived from substituted acetylnaphthalenes participated also successfully in the reaction, furnishing the desired products **3n−3q** in 83–98% yields. Similarly, biaryl substituted *N*-tosylhydrazones could also be used in this transformation, delivering the desired products **3r−3t** in 83–97% yields. The *N*-tosylhydrazone synthesized from trans-4-phenyl-3-buten-2-one participated readily into this reaction as well. And the double bond in the substrate **1** was delivered into the structure of the expected product **3u**, without any damage. While good yield was obtained with acetophenone-derived *N*-tosylhydrazones, the introduction of a functional group on the carbon of the imine group significantly decreased the reactivity of substrate **1**. As a result, the *N*-tosylhydrazones came from 2-phenylacetophenone, 1*H*-indene-1,3(2*H*)-dione, and propiophenone, reluctantly engaged in the reaction, giving **3v−3x** in <50% yield. The usefulness of this method was demonstrated by a very efficient synthesis of the neuroprotective reagent **3y**. Through a reaction of 1-(3,4,5-trimethoxyphenyl)ethan-1-one *N*-tosylhydrazone with sulfur, **3y** was obtained in 85% yield. It should be noted that, although the reported method for synthesizing **3y** started also from the same *N*-tosylhydrazone derivative (Thomas et al., 1985), owing to the use of highly toxic reagent, thionyl chloride, our method can be considered as a green protocol for implementing the synthesis of **3y**. Furthermore, the transformations of aliphatic and heterocyclic *N*-tosylhydrazones with sulfur were also investigated, but the desired products **3z** and **4a** were not obtained. We also tried to use selenium or tellurium instead of sulfur to perform this reaction, but unfortunately, none of them succeeded.

**Scheme 4:**
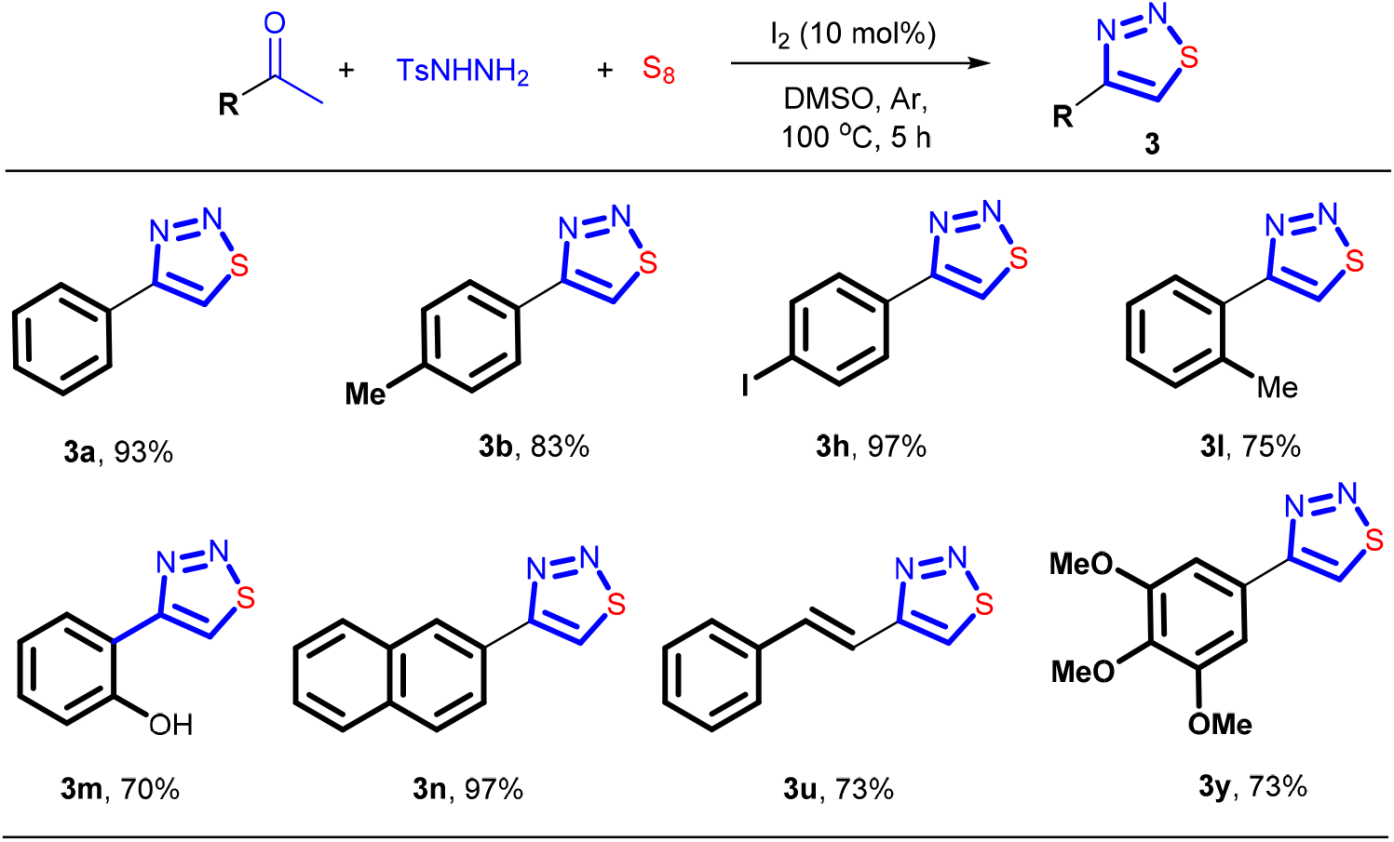
The synthesis of 4-aryl-1,2,3-thiadiazoles via one-pot fashion. Reaction conditions: 0.3 mmol of aryl ketone, 0.33 mmol of TsNHNH_2_, 0.6 mmol of S_8_, I_2_ (10 mol%), DMSO (3 mL), 5 h, under argon. Isolated yield.

Since *N*-tosylhydrazone **1** can be easily formed from *p*-toluenesulfonhydrazide and a ketone (Sun et al., 2018; El-Harairy et al., 2019a,b; Li et al., 2019; Liu et al., 2019), we then investigated whether or not the cyclization reaction could be carried out in one-pot fashion directly using a ketone and the hydrazide as the precursors. If it was established, the isolation and purification of the *N*-tosylhydrazone component can be avoided, thus significantly strengthening the synthetic efficiency. The results showed that this idea was indeed feasible, and the cyclization products were obtained with 70–97% yields (Scheme 4). To ensure a good yield of the reaction, all the three-component reactions were performed under argon atmosphere. It should be noted that 2-hydroxyacetophone that contains a reactive arene ring toward electrophilic iodination can tolerate the I_2_-based conditions. The corresponding product **3m** can be formed in 70% yield.

**Scheme 5:**
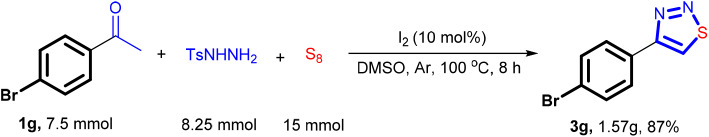
A gram-scale synthesis of **3g**.

The neuroprotective reagent **3y** can also be synthesized in this way, but the yield obtained is slightly inferior compared with the method in Scheme 3. Despite this fact, this three-component protocol is quite promising as it saved one step while minimized the generation of waste. The reaction can also be carried out on a gram-scale synthesis. For instance, the reaction performed well using 7.5 mmol of 4-bromoacetophenone, 8.25 mmol of TsNHNH_2_, and 15 mmol of sulfur, leading to isolation of 1.57 g of the product **3a** with 87% of yield (Scheme 5).

**Scheme 6:**
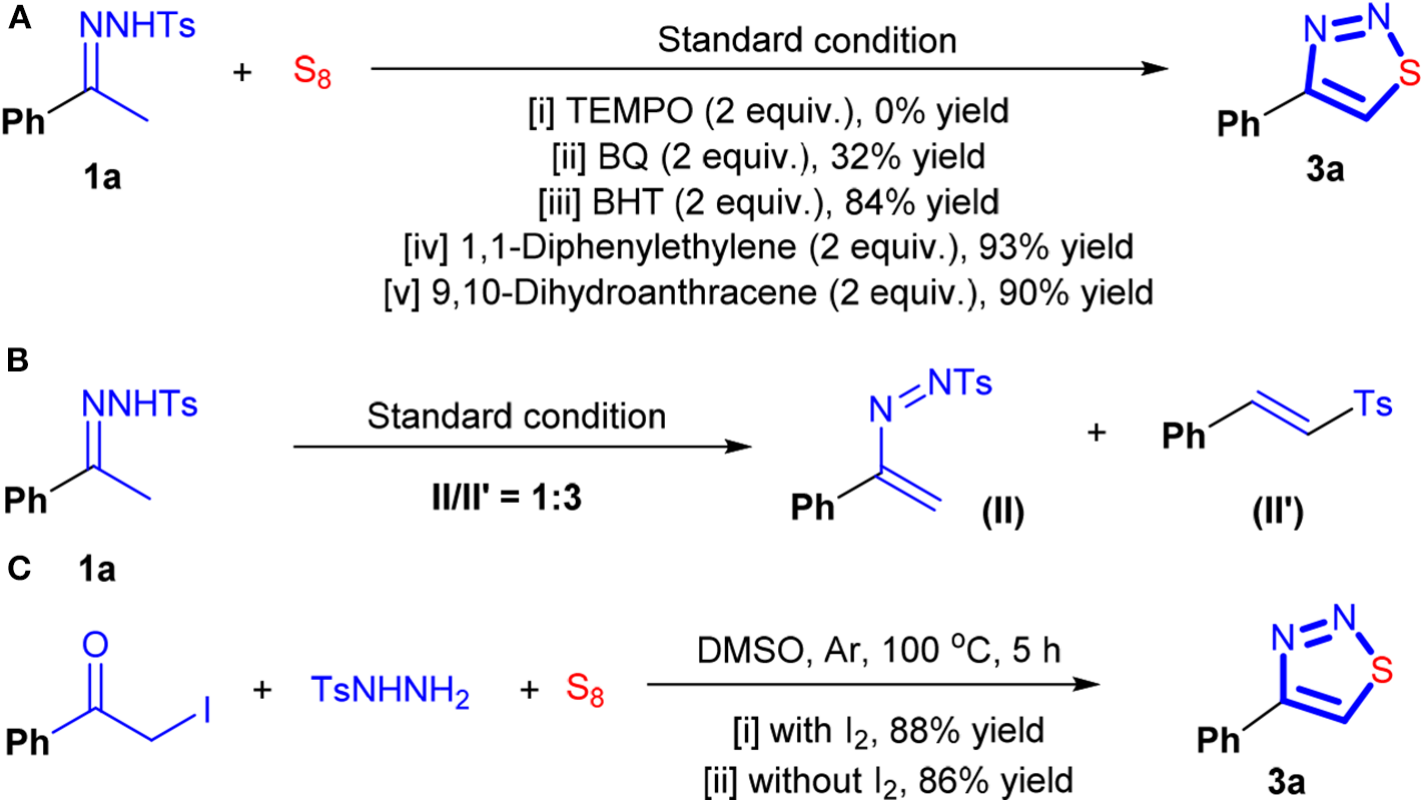
The control experiments.

To shed light on the mechanism, some control experiments were conducted. As shown in Scheme 6, although **1a** was completely consumed, **3a** can be hardly detected when 2.0 equivalents of 2,2,6,6-tetramethylpiperidinooxy (TEMPO) were added. The addition of benzoquinone (BQ) was found to be detrimental for the reaction either. But in this case, the reaction was not quenched, and it proceeded slowly, and **3a** can be isolated in 32% yield under the identical conditions. The effects of 9,10-dihydroanthracene and 1,1-diphenylethylene, which are acid-compatible radical scavengers, on the model reaction were also investigated. And **3a** can be isolated in 93 and 90% yields, respectively. The reaction proceeded uneventfully in the presence of 2,6-di-tert-butyl-4-methylphenol (BHT). In the absence of sulfur, decomposition of **1a** occurred, providing II′ as the main product (Pramanik et al., 2019). In this case, the isolated II′ is not pure, and the ^1^H and ^13^C NMR spectra led us to have speculation on the formation of II. HRMS also supported our speculation as a peak at 287.0575 (M + H^+^) can be observed. The treatment of the mixture of 2-iodo-1-phenylethan-1-one and TsNHNH_2_ with sulfur resulted in the formation of **3a**. It should be noted that, when DMSO was used as solvent, the transformation was always successful either in the presence or in the absence of I_2_. However, replacing DMSO with the other solvents, such as DMF and toluene, resulted in a dramatic loss of the reaction yield. These results indicated that the choice of solvent is the key to ensure a good yield of this cyclization.

**Scheme 7:**
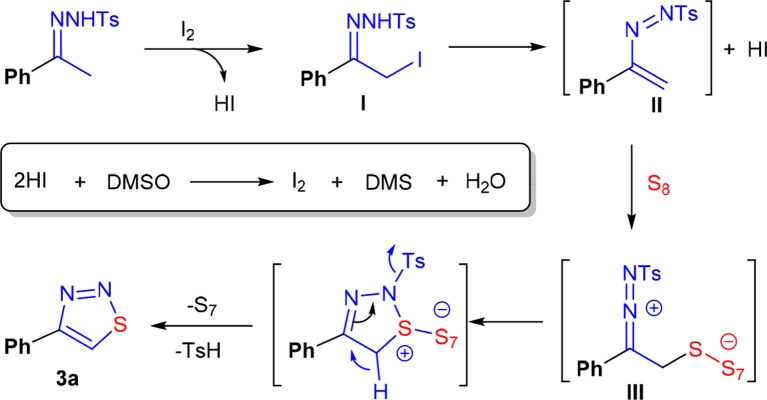
Proposed mechanism.

Based on all these observations, a plausible mechanism was proposed. We conjectured that the reaction followed a polar reaction mechanism rather than a free-radical mechanism. As shown in Scheme 7, the initial event should be α-iodation of **1a**, which gives I as an intermediate. Then, an elimination of one molecule of HI of I occurred, providing an intermediate II. Because of the presence of an electron-rich vinyl group, this species behaves like a nucleophile, can thus react with sulfur to form an intermediate III (Chen et al., 2015; Ishikawa et al., 2017; Liu et al., 2018; Li et al., 2019). Finally, **3a** was formed through an intramolecular cyclization and the following elimination of TsH and S_7_. In the first two steps of the reaction, two molecules of HI were generated. To establish a catalytic cycle, HI must be oxidized to I_2_. The unique oxidizing ability of the solvent DMSO played the key role in regenerating I_2_ (Steuer et al., 2011; Deshidi et al., 2014, 2015; Kalmode et al., 2014, 2015; Wu et al., 2014; Mohammed et al., 2015; Mupparapu et al., 2015). The combination of I_2_ and DMSO ensured the success of this synthetic reaction.

## Conclusion

In summary, we have developed an iodine-catalyzed cyclization of *N*-tosylhydrazones with sulfur using DMSO a dual solvent and oxidant. This reaction provided an efficient approach to produce diversified 4-aryl-1,2,3-thiadiazoles in good yields. This method can be used in a gram scale synthesis. Furthermore, a one-pot synthesis was also established, which allowed the direct use of ketone as substrate, without isolating the *N*-tosylhydrazone intermediate. This approach was proven also to be applicable in the synthesis of a neuroprotective reagent. An eminent advantage of this strategy is that it avoided the use of external oxidants. Considering expensive photochemical catalyst or electrochemical instruments are required in the previously reported methods, the present method can be considered as a practically applicable and environmentally benign approach for the synthesis of 1,2,3-thiadiazoles.

## Materials and Methods

Chemicals were obtained commercially and used as received. NMR spectra were recorded on a Bruker DPX−400 spectrometer using TMS as the internal standard. DMSO as solvent was used directly without any treatment. All products were isolated by short chromatography on a silica gel (200–300 mesh) column using petroleum ether (60–90°C), unless otherwise noted. All of reagents were of analytical grade quality, purchased from Adamas-beta Pharmaceuticals, Inc.

### General Procedure for I_2_/DMSO-Catalyzed Transformation From *N*-tosylhydrazones and Sulfur to 4-aryl 1,2,3-thiadiazoles

A mixture of substituted *N*-tosylhydrazones (0.3 mmol), sulfur (0.6 mmol), I_2_ (10 mol%) were loaded into a Schlenk tube (25 mL). Then, the tube was degassed for 30 s and filled with Argon. This process was repeated for a total of three times. Afterward, DMSO (3 mL) was added under an argon atmosphere. The resulting reaction mixture was stirred and heated to 100°C for 5 h. After reaction completion, the solution was quenched the saturated solution of sodium thiosulfate (5 mL) and extracted with EtOAc (3 × 10 mL). The combined EtOAc extracts were dried over anhydrous Na_2_SO_4_, filtered, and concentrated under reduced pressure. The crude residue was purified by flash column chromatography on silica gel using PE/EtOAc as the eluent.

### General Procedure for the Synthesis of 4-aryl-1,2,3-thiadiazoles via One-Pot Fashion

A Schlenk tube (25 mL) equipped with a stir bar was charged with TsNHNH_2_ (0.33 mmol), sulfur (0.6 mmol), I_2_ (10 mol%). Then, the tube was degassed for 30 s and filled with Argon. This process was repeated for a total of three times. Afterward, aryl ketone (0.3 mmol) and DMSO (3 mL) was added under an argon atmosphere. The resulting reaction mixture was stirred and heated to 100°C for 5 h. After reaction completion, the solution was quenched the saturated solution of sodium thiosulfate (5 mL) and extracted with EtOAc (3 × 10 mL). The combined EtOAc extracts were dried over anhydrous Na_2_SO_4_, filtered, and concentrated under reduced pressure. The crude residue was purified by flash column chromatography on silica gel using PE/EtOAc as the eluent.

## Data Availability Statement

The raw data supporting the conclusions of this article will be made available by the authors, without undue reservation, to any qualified researcher.

## Author Contributions

PL, YG, and LV constructed the workflow. WL, JZ, and JH synthesized and purified the compounds. LX performed the NMR spectrometric analysis. PL and YG completed the paper.

## Conflict of Interest

The authors declare that the research was conducted in the absence of any commercial or financial relationships that could be construed as a potential conflict of interest.
