# Supplementary material for: I2/DMSO-Catalyzed Transformation of N-tosylhydrazones to 1,2,3-thiadiazoles
Source: Front Chem. 2020 Jun 12;8:466. doi: 10.3389/fchem.2020.00466 (PMC7304252; doi:10.3389/fchem.2020.00466)
Supplement: Supplementary file 1 [file Data_Sheet_1.PDF]

I_2/DMSO-Catalyzed Transformation of *N*-Tosylhydrazones to 1,2,3-Thiadiazoles

Weiwei Li, [1] Jun Zhang, [1] Jing He,[1] Liang Xu, [1] Luigi Vaccaro,[3] Ping Liu,*[1] Yanlong Gu,*[2] and Bin Dai[1]

[1] School of Chemistry and Chemical Engineering, the Key Laboratory for Green Processing of Chemical Engineering of Xinjiang Bingtuan, Shihezi University, Shihezi City, 832004, China.

[2] Key Laboratory of Material Chemistry for Energy Conversion and Storage, Ministry of Education, Hubei Key Laboratory of Material Chemistry and Service Failure, School of Chemistry and Chemical Engineering, Huazhong University of Science & Technology, 1037 Luoyu road, Hongshan District, Wuhan 430074, China.

[3] Laboratory of Green S.O.C. – Dipartimento di Chimica, biologia e Biotecnologie, Università degli Studi di Perugia, Via Elce di Sotto 8, 06123 – Perugia

E-mail: liuping1979112@aliyun.com (P. Liu) and klgyl@hust.edu.cn (Y. Gu)

Experimental Section... 2
References.. 8
The Spectra of ^{1}H NMR and ^{13}C NMR... 9

Experimental Section

Materials and instruments

Chemicals were obtained commercially and used as received. NMR spectra were recorded on a Bruker DPX–400 spectrometer using TMS as the internal standard. DMSO as solvent was used directly without any treatment. All products were isolated by short chromatography on a silica gel (200–300 mesh) column using petroleum ether (60–90 °C), unless otherwise noted. All of reagents were of analytical grade quality, purchased from Adamas-beta Pharmaceuticals, Inc.

General procedure for the synthesis of *aryl sulfonylhydrazones* ***1***

To a round bottom flask (25 mL), anhydrous ethanol (3 mL), aryl ketone (2 mmol), and aryl sulfonyl hydrazide (2.1 mmol) were added. The reaction mixture was stirred at 60 °C for 4 h, and the complete consumption of aryl ketone was confirmed by TLC. The solution was cooled until solid precipitate. The product aryl sulfonylhydrazone **1** was obtained by filtering, washing with petroleum ether, and drying in vacuo.

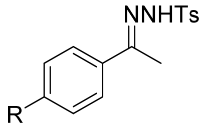 **1a**, R = H, 98%<br>**1b**, R = CH_3, 98%<br>**1c**, R = n-C_4H_9, 85%<br>**1d**, R = MeO, 93%<br>**1e**, R = F, 88%<br>**1f**, R = Cl, 85%<br>**1g**, R = Br, 85%<br>**1h**, R = I, 80%<br>**1i**, R = CF_3, 92%	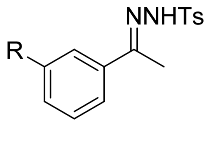<br>**1j**, R = NO_2, 70%<br>**1k**, R = MeO, 90%	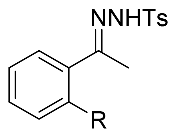<br>**1l**, R = CH_3, 90%<br>**1m**, R = OH, 75%
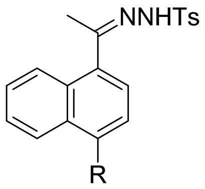<br>**1n**, R = H, 98%<br>**1o**, R = F, 80%	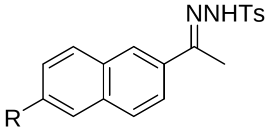<br>**1p**, R = H, 95%<br>**1q**, R = OMe, 90%	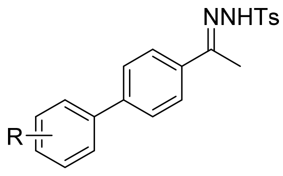<br>**1r**, R = H, 98%<br>**1s**, R = 3-Me, 65%<br>**1t**, R = 4-Me, 80%
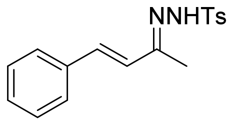<br>**1u**, Yield: 98%	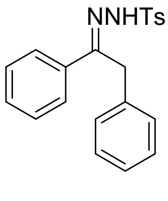<br>**1v**, Yield: 60%	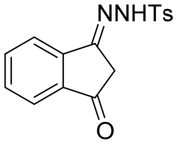<br>**1w**, Yield: 58%

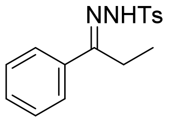 **1x**, Yield: 65%	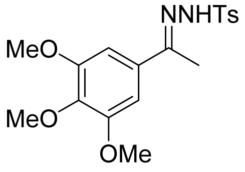 **1y**, Yield: 95%	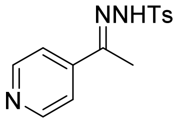 **1z**, Yield: 98%
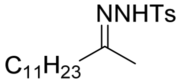 **1aa**, Yield: 85%	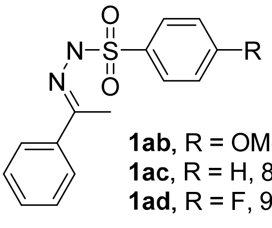 **1ab**, R = OMe, 93%<br>**1ac**, R = H, 85%<br>**1ad**, R = F, 90%	

General procedure for a I_2/DMSO-catalyzed transformation from N-tosylhydrazones and sulfur to 4-aryl 1,2,3-thiadiazoles

A mixture of substituted *N*-tosylhydrazones (0.3 mmol), sulfur (0.6 mmol), I_2 (10 mol%) were loaded into a Schlenk tube (25 mL). Then, the tube was degassed for 30 s and filled with Argon. This process was repeated for a total of three times. Afterward, DMSO (3 mL) was added under an argon atmosphere. The resulting reaction mixture was stirred and heated to 100 °C for 5 h. After reaction completion, the solution was quenched the saturated solution of sodium thiosulfate (5 mL) and extracted with EtOAc (3 × 10 mL). The combined EtOAc extracts were dried over anhydrous Na_2SO_4, filtered and concentrated under reduced pressure. The crude residue was purified by flash column chromatography on silica gel using PE/EtOAc as the eluent.

General procedure for the synthesis of 4-aryl-1,2,3-thiadiazoles via one-pot fashion

A Schlenk tube (25mL) equipped with a stir bar was charged with $TsNHNH_2$ (0.33 mmol), sulfur (0.6 mmol), I_2 (10 mol%). Then, the tube was degassed for 30 s and filled with Argon. This process was repeated for a total of three times. Afterward, aryl ketone (0.3 mmol) and DMSO (3 mL) was added under an argon atmosphere. The resulting reaction mixture was stirred and heated to 100 °C for 5 h. After reaction completion, the solution was quenched the saturated solution of sodium thiosulfate (5 mL) and extracted with EtOAc (3 × 10 mL). The combined EtOAc extracts were dried over anhydrous Na_2SO_4, filtered and concentrated under reduced pressure. The crude residue was purified by flash column chromatography on silica gel using PE/EtOAc as the eluent.

*4-Phenyl-1,2,3-thiadiazole [**3a**]* [1]

White solid; Yield: 44.8 mg (92%); mp 76-77 ºC (lit., 76-77 ºC); ^{1}H NMR (400 MHz, $CDCl_3$): δ 8.65 (s, 1H), 8.04 (d, *J* = 8.0 Hz, 2H), 7.51 (t, *J* = 8.0 Hz, 2H), 7.44 (t, *J* = 8.0 Hz, 1H).^{13}C NMR (101 MHz, $CDCl_3$): δ 162.88, 130.81, 130.04, 129.45, 129.19, 127.41.

*4-(p-Tolyl)-1,2,3-thiadiazole [**3b**]* [1]

White solid; Yield: 43.6 mg (83%); mp 74-75 ºC (lit., 74-75 ºC); ^{1}H NMR (400 MHz, $CDCl_3$): δ 8.58 (s, 1H), 7.92 (d, *J* = 8.0 Hz, 2H), 7.30 (d, *J* = 8.0 Hz, 2H), 2.41 (s, 3H). ^{13}C NMR (101 MHz, $CDCl_3$): δ 162.97, 139.50, 129.86, 129.38, 128.06, 127.30, 21.38.

*4-(4-Butylphenyl)-1,2,3-thiadiazole [**3c**]* [2]

White solid; Yield: 58.3 mg (89%); mp 68-69 ºC (lit., 68-69 ºC); ^{1}H NMR (400 MHz, $CDCl_3$): δ 8.58 (s, 1H), 7.94 (d, *J* = 8.0 Hz, 2H), 7.31 (d, *J* = 8.0 Hz, 2H), 2.67 (t, *J* = 8.0 Hz , 2H), 1.68 – 1.60 (m, 2H), 1.43 – 1.34 (m, 2H), 0.94 (t, *J* = 8.0 Hz, 3H). ^{13}C NMR (101MHz, $CDCl_3$): δ 163.01, 144.52, 129.36, 129.23, 128.26, 127.32, 35.48, 33.49, 22.36, 13.98.

*4-(4-Methoxyphenyl)-1,2,3-thiadiazole [**3d**]* [1]

White solid; Yield: 50.9 mg (88%); mp 89-90 ºC (lit., 89-90 ºC); ^{1}H NMR (400 MHz, $CDCl_3$): δ 8.43 (s, 1H), 7.90 – 7.86 (m, 2H), 6.95 – 6.91 (m, 2H), 3.78 (s, 3H). ^{13}C NMR (101MHz, $CDCl_3$): δ 162.70, 160.52, 128.74, 128.50, 123.55, 114.54, 55.40.

*4-(4-Fluorophenyl)-1,2,3-thiadiazole [**3e**]* [1]

White solid; Yield: 48.7 mg (90%); mp 101-102 ºC (lit., 101-102 ºC); ^{1}H NMR (400 MHz, $CDCl_3$): δ 8.61 (s, 1H), 8.06 – 8.01 (m, 2H), 7.23 – 7.17 (m, 2H). ^{13}C NMR (101 MHz, $CDCl_3$): δ 163.38 (d, $J_{C\text{-}F}$=250.48 Hz), 161.85, 129.77, 129.25 (d, $J_{C\text{-}F}$=9.09 Hz), 127.08 (d, $J_{C\text{-}F}$=4.04 Hz), 116.24 (d, $J_{C\text{-}F}$=22.22 Hz).

*4-(4-Chlorophenyl)-1,2,3-thiadiazole [**3f**]* [1]

White solid; Yield: 50.8 mg (86%); mp 136-137 ºC (lit., 136-137 ºC); ^{1}H NMR (400 MHz, $CDCl_3$): δ 8.65 (s, 1H), 8.01 – 7.98 (m, 2H), 7.51 – 7.47 (m, 2H). ^{13}C NMR (101 MHz, $CDCl_3$): δ 161.73, 135.43, 130.18, 129.43, 129.29, 128.63.

*4-(4-Bromophenyl)-1,2,3-thiadiazole [**3g**]* [1]

White solid; Yield: 59.2 mg (82%)/1.57 g (87%, gram-scale synthesis); mp 150-151 ºC (lit., 150-151 ºC); ^{1}H NMR (400 MHz, $CDCl_3$): δ 8.66 (s, 1H), 7.94 – 7.91 (m, 2H), 7.66 –

7.62 (m, 2H). ^{13}C NMR (101 MHz, $CDCl_3$): δ 161.75, 132.38, 130.28, 129.72, 128.87, 123.66.

*4-(4-Iodophenyl)-1,2,3-thiadiazole [**3h**]* [1]

White solid; Yield: 79.6 mg (92%); mp 161-162 ºC (lit., 161-162 ºC); ^{1}H NMR (400 MHz, $CDCl_3$): δ 8.66 (s, 1H), 7.85 (d, *J* = 8.0 Hz, 2H), 7.79 (d, *J* = 8.0 Hz, 2H). ^{13}C NMR (101 MHz, $CDCl_3$): δ 161.86, 138.34, 130.28, 128.97, 95.42.

*4-(4-(Trifluoromethyl)phenyl)-1,2,3-thiadiazole [**3i**]* [1]

White solid; Yield: 56.7 mg (82%); mp 92-93 ºC (lit., 92-93 ºC); ^{1}H NMR (400 MHz, $CDCl_3$): δ 8.70 (s, 1H), 8.09 (d, *J* = 8.0 Hz, 2H), 7.69 (d, *J* = 8.0 Hz, 2H). ^{13}C NMR (101 MHz, $CDCl_3$): δ 161.34, 134.07, 131.48, 131.23 (q, J_{C-F}=33.33 Hz), 127.65, 126.18 (q, J_{C-F}=4.04Hz), 123.9 (q, J_{C-F}=272.7 Hz).

*4-(3-Nitrophenyl)-1,2,3-thiadiazole [**3j**]* [3]

White solid; Yield: 57.0 mg (91%); mp 175-176 ºC (lit., 209-211 ºC); ^{1}H NMR (400 MHz, $CDCl_3$): δ 8.79 (s, 1H), 8.39 (dt, *J* = 7.8, 1.2 Hz, 1H), 8.24 (ddd, *J* = 8.3, 2.3, 1.1 Hz, 1H), 7.65 (t, *J* = 8.0 Hz, 1H). ^{13}C NMR (101 MHz, $CDCl_3$): δ 160.41, 148.82, 133.17, 132.42, 131.83, 130.35, 124.02, 122.15.

*4-(3-Methoxyphenyl)-1,2,3-thiadiazole [**3k**]* [3]

White solid; Yield: 53.1 mg (92%); mp 84-85 ºC (lit., 70-73 ºC); ^{1}H NMR (400 MHz, $CDCl_3$): δ 8.64 (s, 1H), 7.65 (s, 1H), 7.56 (d, *J* = 4.0 Hz, 1H), 7.40 (t, *J* = 8.0 Hz, 1H), 6.99 (dd, *J* = 4.0, 2.0 Hz, 1H), 3.88 (s, 3H). ^{13}C NMR (101 MHz, $CDCl_3$) δ 162.72, 160.19, 132.03, 130.31, 130.23, 119.70, 115.37, 112.73, 55.45.

*4-(o-Tolyl)-1,2,3-thiadiazole [**3l**]* [1]

White solid; Yield: 47.4 mg (90%); mp 74-75 ºC (lit., 74-75 ºC); ^{1}H NMR (400 MHz, $CDCl_3$): δ 8.51 (s, 1H), 7.64 (d, *J* = 8.0 Hz, 1H), 7.41 – 7.29 (m, 3H), 2.45 (s, 3H). ^{13}C NMR (101 MHz, $CDCl_3$): δ 162.55, 136.74, 133.01, 131.14, 130.49, 130.37, 129.33, 126.24, 21.02.

*2-(1,2,3-Thiadiazol-4-yl)phenol [**3m**]* [3]

White solid; Yield: 38.3 mg (72%); mp 102-103 ºC (lit., 155-157 ºC); ^{1}H NMR (400 MHz, $CDCl_3$): δ 10.53 (s, 1H), 8.79 (s, 1H), 7.64 (d, *J* = 4.0 Hz, 1H), 7.35 (t, *J* = 8.0 Hz, 1H),

7.13 (d, J = 8.0 Hz, 1H), 6.98 (t, J = 8.0 Hz, 1H). ^{13}C NMR (101 MHz, $CDCl_3$) δ 162.21, 156.00, 131.36, 130.13, 127.51, 120.11, 118.27, 114.62.

*4-(Naphthalen-1-yl)-1,2,3-thiadiazole [**3n**]* [3]

White solid; Yield: 58.5 mg (92%); mp 198-200 ºC (lit., 197-201 ºC); ^{1}H NMR (400 MHz, $CDCl_3$): δ 8.66 (s, 1H), 8.11 – 8.04 (m, 1H), 7.99 – 7.91 (m, 2H), 7.75 (d, J = 6.9 Hz, 1H), 7.61 – 7.47 (m, 3H). ^{13}C NMR (101 MHz, $CDCl_3$): δ 161.97, 134.24, 133.90, 131.47, 130.04, 128.59, 128.45, 127.13, 126.36, 125.31, 125.16.

*4-(4-Fluoronaphthalen-1-yl)-1,2,3-thiadiazole [**3o**]*[2]

White solid; Yield: 57.0 mg (83%); mp 207-208 ºC (lit., 207-208 ºC); ^{1}H NMR (400 MHz, $CDCl_3$): δ 8.64 (s, 1H), 8.19 (d, J = 7.6 Hz, 1H), 8.06 (d, J = 7.6 Hz, 1H), 7.66 (dd, J = 8.0, 5.4 Hz, 1H), 7.54-7.62 (m, 2H), 7.22 (dd, J = 10.0, 7.9 Hz, 1H). ^{13}C NMR (101 MHz, $CDCl_3$): δ 161.32, 159.61 (d, $J_{C\text{-}F}$=256.54 Hz), 134.29, 132.88 (d, $J_{C\text{-}F}$=5.05 Hz), 128.59 (d, $J_{C\text{-}F}$=9.09 Hz), 128.06, 126.70 (d, $J_{C\text{-}F}$=2.02 Hz), 125.26 (d, $J_{C\text{-}F}$=3.03 Hz), 124.61 (d, $J_{C\text{-}F}$=4.04 Hz), 124.04 (d, $J_{C\text{-}F}$=16.16 Hz), 121.02 (d, $J_{C\text{-}F}$=6.06 Hz), 109.16 (d, $J_{C\text{-}F}$=20.2 Hz).

*4-(Naphthalen-2-yl)-1,2,3-thiadiazole [**3p**]* [1]

White solid; Yield: 63.3 mg (98%); mp 202-203 ºC (lit., 202-203 ºC); ^{1}H NMR (400 MHz, $CDCl_3$): δ 8.68 (s, 1H), 8.55 (s, 1H), 8.05 (dd, J = 8.5, 1.7 Hz, 1H), 7.92 (d, J = 8.1 Hz, 2H), 7.88 – 7.82 (m, 1H), 7.54 – 7.49 (m, 2H). ^{13}C NMR (101 MHz, $CDCl_3$) δ 162.90, 133.66, 133.48, 130.19, 129.00, 128.54, 128.08, 127.84, 126.94, 126.89, 126.81, 124.76.

*4-(6-Methoxynaphthalen-2-yl)-1,2,3-thiadiazole [**3q**]* [2]

White solid; Yield: 68.8 mg (95%); mp 150-151ºC (lit., 150-151 ºC); ^{1}H NMR (400 MHz, $CDCl_3$): δ 8.67 (s, 1H), 8.50 (s, 1H), 8.05 (dd, J = 8.6, 1.8 Hz, 1H), 7.83 (dd, J = 8.6, 6.8 Hz, 2H), 7.22 – 7.14 (m, 2H), 3.94 (s, 3H). ^{13}C NMR (101 MHz, $CDCl_3$): δ 163.12, 158.50, 135.00, 130.04, 129.41, 128.92, 127.74, 126.71, 125.99, 125.31, 119.69, 105.79, 55.39.

*4-([1,1'-Biphenyl]-4-yl)-1,2,3-thiadiazole [**3r**]* [1]

White solid; Yield: 69.9mg (97%); mp 183-184ºC (lit., 183-184 ºC); ^{1}H NMR (400 MHz, $CDCl_3$): δ 8.68 (s, 1H), 8.13 (d, J = 8.2 Hz, 2H), 7.75 (d, J = 8.2 Hz, 2H), 7.68 – 7.64 (m,

2H), 7.48 (t, J = 7.7 Hz, 2H), 7.39 (t, J = 7.3 Hz, 1H). ^{13}C NMR (101 MHz, $CDCl_3$): δ 162.60, 142.22, 140.24, 129.83, 129.71, 128.93, 127.84, 127.82, 127.09.

*4-(3'-Methyl-[1,1'-biphenyl]-4-yl)-1,2,3-thiadiazole [**3s**]* [2]

White solid; Yield: 65.7 mg (87%); mp 182-183ºC (lit., 182-183 ºC); ^{1}H NMR (400 MHz, $CDCl_3$): δ 8.65 (s, 1H), 8.15 – 8.07 (m, 2H), 7.77 – 7.69 (m, 2H), 7.45 (d, J = 8.9 Hz, 2H), 7.36 (t, J = 7.6 Hz, 1H), 7.20 (d, J = 7.5 Hz, 1H), 2.44 (s, 3H). ^{13}C NMR (101 MHz, $CDCl_3$): δ 162.63, 142.32, 140.20, 138.56, 129.82, 129.62, 128.85, 128.55, 127.84, 127.77, 124.20, 21.59.

*4-(4'-Methyl-[1,1'-biphenyl]-4-yl)-1,2,3-thiadiazole [**3t**]* [2]

White solid; Yield: 62.7 mg (83%); mp 186-187ºC (lit., 186-187 ºC); ^{1}H NMR (400 MHz, $CDCl_3$): δ 8.64 (s, 1H), 8.13 – 8.06 (m, 2H), 7.77 – 7.69 (m, 2H), 7.57 – 7.52 (m, 2H), 7.28 (d, J = 8.0 Hz, 2H), 2.41 (s, 3H). ^{13}C NMR (101 MHz, $CDCl_3$): δ 162.66, 142.13, 137.67, 137.32, 129.72, 129.66, 129.42, 127.79, 127.60, 126.91, 21.18.

*(E)-4-styryl-1,2,3-thiadiazole [**3u**]* [4]

White solid; Yield: 40.2 mg (72%); mp 80-81ºC (lit., 80-81 ºC); ^{1}H NMR (400 MHz, $CDCl_3$) δ 8.37 (s, 1H), 7.71 (d, *J* = 16.3 Hz, 1H), 7.57 (d, *J* = 7.0 Hz, 2H), 7.43 (d, *J* = 8.4 Hz, 1H), 7.39 (d, *J* = 7.3 Hz, 2H), 7.36 – 7.30 (m, 1H). ^{13}C NMR (101 MHz, $CDCl_3$) δ 161.27, 136.15, 134.69, 130.23, 128.87, 128.72, 126.94, 117.11.

*4,5-diphenyl-1,2,3-thiadiazole[**3v**]* [5]

White solid; Yield: 25.0 mg (35%); mp 92-93ºC (lit., 92-93 ºC); ^{1}H NMR (400 MHz, $CDCl_3$) δ 7.99 (s, 1H), 7.97 (s, 1H), 7.69 – 7.61 (m, 2H), 7.51 (t, *J* = 7.8 Hz, 3H), 7.42 – 7.34 (m, 3H).^{13}C NMR (101 MHz, $CDCl_3$) δ 134.91, 133.00, 131.61, 129.92, 129.35, 129.20, 129.17, 129.04, 128.73, 128.35.

*8H-indeno[1,2-d][1,2,3]thiadiazol-8-one[**3w**]* [6]

Yellow solid; Yield: 19.8 mg (45%); mp 124-125ºC (lit., 124-125 ºC); ^{1}H NMR (400 MHz, $CDCl_3$) δ 7.77 (d, J = 7.4 Hz, 1H), 7.69 (d, J = 7.3 Hz, 1H), 7.58 (td, J = 7.6, 1.1 Hz, 1H), 7.38 (td, J = 7.6, 1.0 Hz, 1H). ^{13}C NMR (101 MHz, $CDCl_3$) δ 179.39, 177.25, 147.20, 138.70, 136.20, 135.56, 130.22, 126.54, 121.63.

*5-methyl-4-phenyl-1,2,3-thiadiazole [**3x**]* [7]

White solid; Yield: 11.1 mg (21%); mp 40-41ºC (lit., 40-41 ºC); ^{1}H NMR (400 MHz,

$CDCl_3$) δ 7.68 (dd, *J* = 8.3, 1.3 Hz, 2H), 7.48 – 7.42 (m, 2H), 7.38 (t, *J* = 7.3 Hz, 1H), 2.64 (s, 3H). ^{13}C NMR (101 MHz, $CDCl_3$) δ 159.72, 146.50, 131.20, 128.83, 128.80, 128.73, 10.55.

*4-(3,4,5-trimethoxyphenyl)-1,2,3-thiadiazole [**3y**]* [8]

White solid; Yield: 64.3 mg (85%); mp 91-93ºC (lit., 91-93 ºC); ^{1}H NMR (400 MHz, $CDCl_3$) δ 8.64 (s, 1H), 7.29 (s, 2H), 3.95 (s, 6H), 3.92 (s, 3H). ^{13}C NMR (101 MHz, $CDCl_3$) δ 162.70, 153.79, 139.16, 129.68, 126.30, 104.68, 61.00, 56.32.

References

[1] S. K. Mo, Q. H. Teng, Y. M. Pan, H. T. Tang, *Adv. Synth. Catal.*, **2019**, 361, 1756-1760.

[2] Li, W., He, J., Liu, P., Zhang, J., Dai, B. *ChemistrySelect*, **2019**, 4, 10587-10590.

[3] J. Chen, Y. Jiang, J. T. Yu, J. Cheng, *J. Org. Chem.*, **2015**, 81, 271-275.

[4] Ishikawa, T., Kimura, M., Kumoi, T., Iida, H. *ACS Catal.*, **2017**, 7, 4986-4989.

[5] Kurandina D, Gevorgyan V. *Org. Lett.*, **2016**, 18, 1804-1807.

[6] L'Abbé G, Dehaen W, Bastin L, et al. *J. Heterocyclic Chem.*, **1992**, 29, 461-465.

[7] Caron M., *J. Org. Chem.*, **1986**, 51, 4075-4077.

[8] Thomas E W, Nishizawa E E, Zimmermann D C, et al. *J. Med. Chem.*, **1985**, 28, 442-446.

The Spectra of ^{1}H NMR and ^{13}C NMR

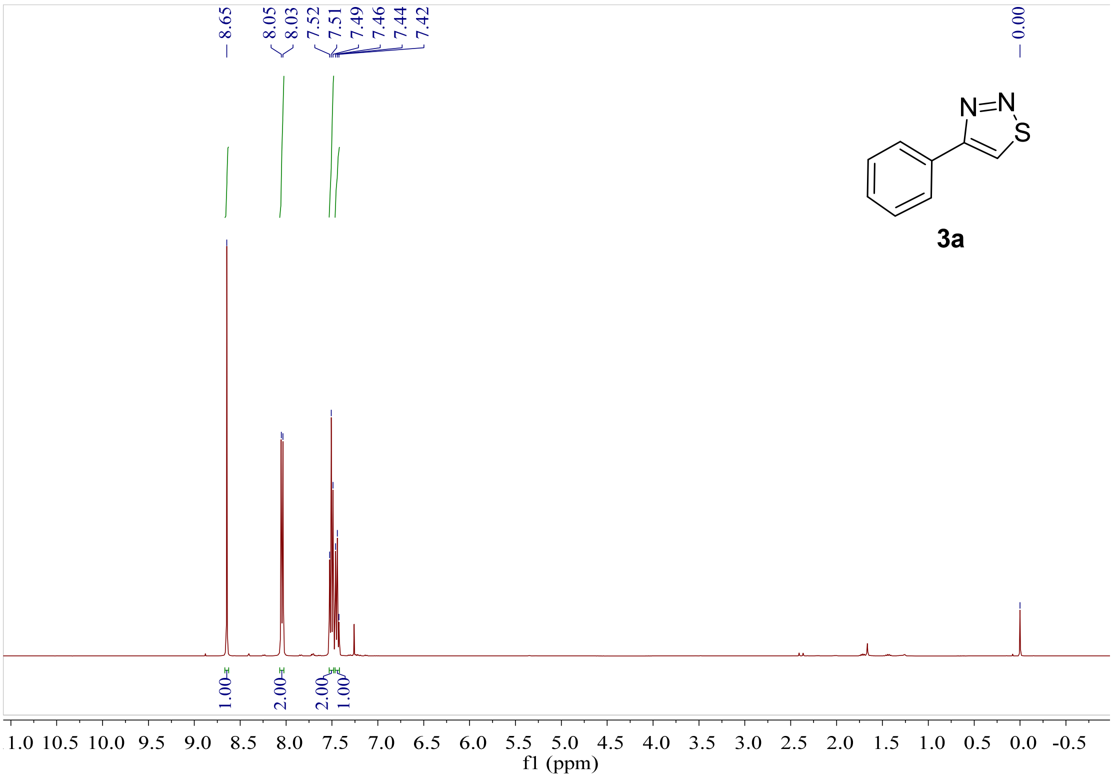

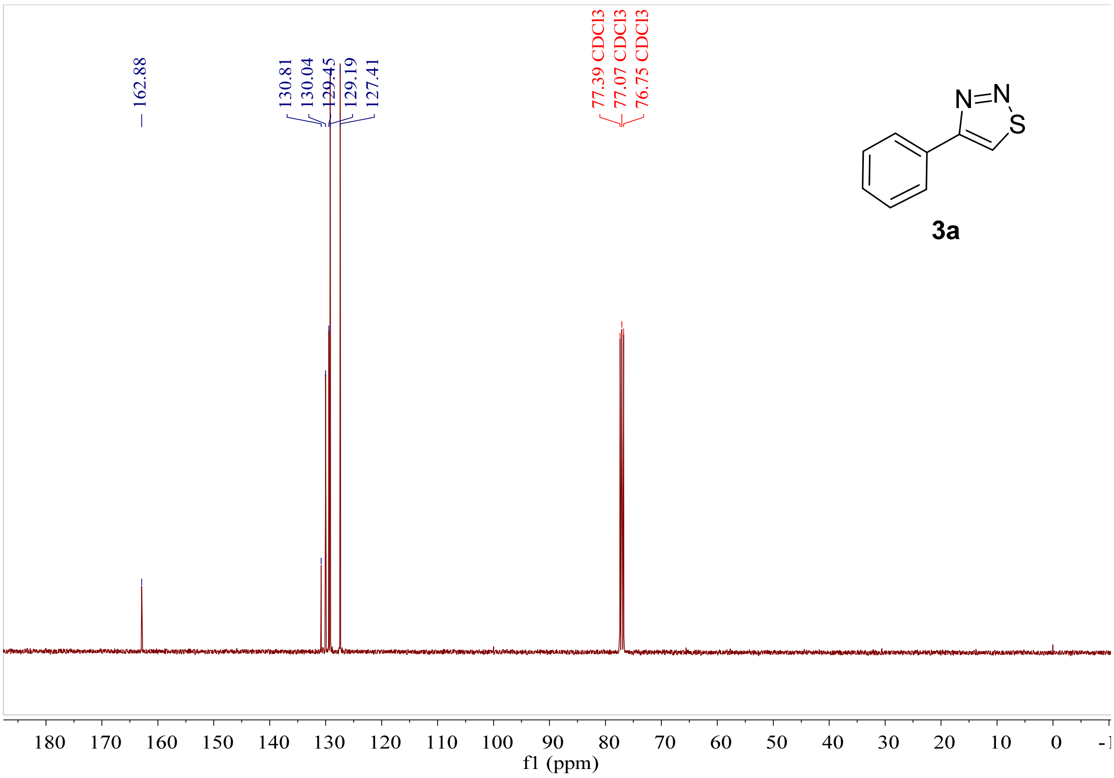

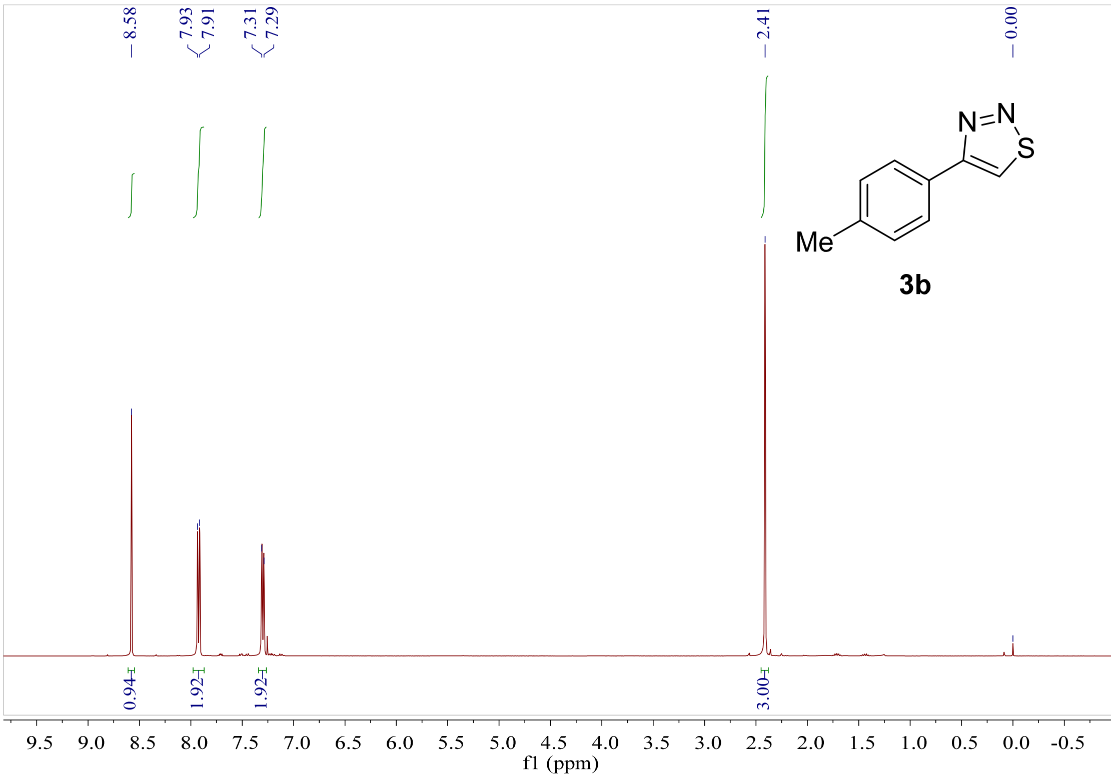
8.58
7.93
7.91
7.31
7.29
2.41
0.00
N=N
S
Me
3b
0.94
1.92
1.92
3.00
9.5 9.0 8.5 8.0 7.5 7.0 6.5 6.0 5.5 5.0 4.5 4.0 3.5 3.0 2.5 2.0 1.5 1.0 0.5 0.0 -0.5
f1 (ppm)

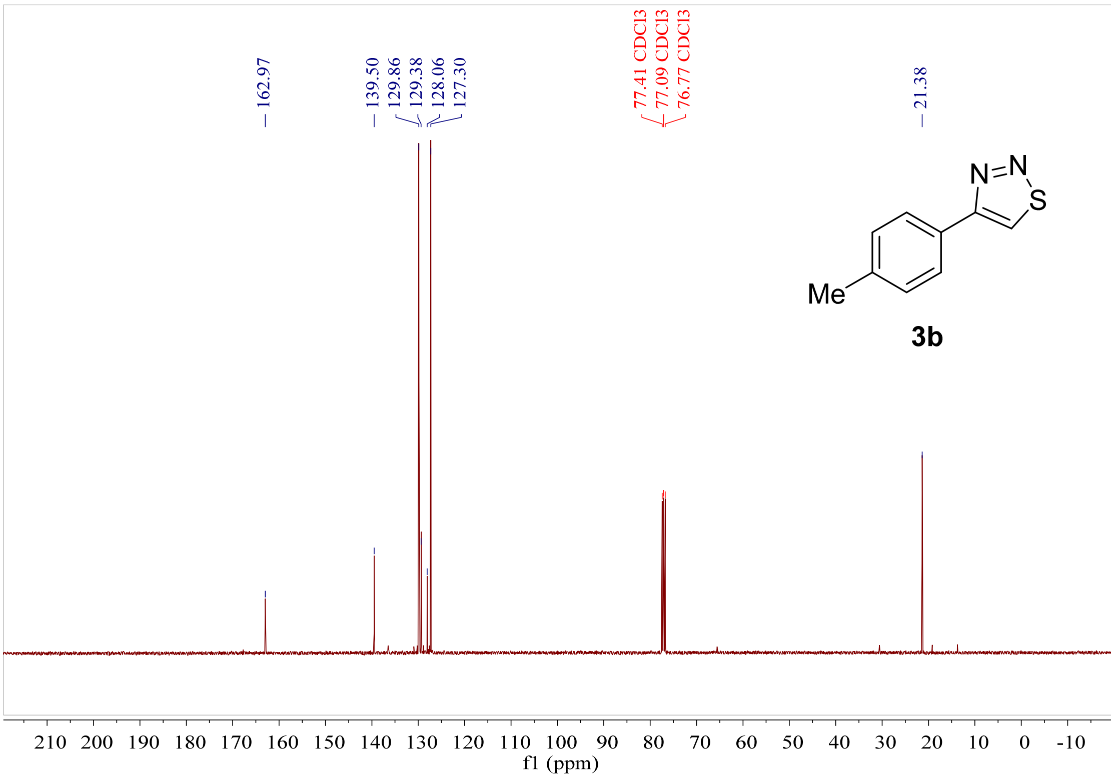
162.97
139.50
129.86
129.38
128.06
127.30
77.41 CDCl3
77.09 CDCl3
76.77 CDCl3
21.38
N=N
S
Me
3b
210 200 190 180 170 160 150 140 130 120 110 100 90 80 70 60 50 40 30 20 10 0 -10
f1 (ppm)

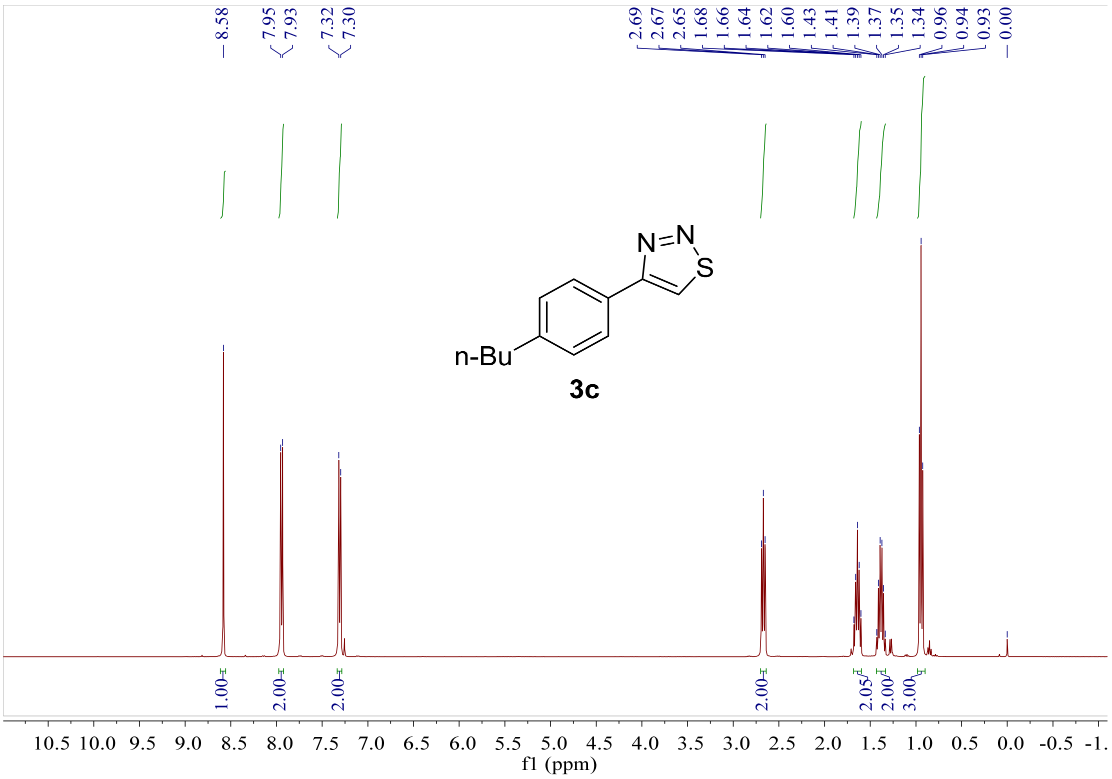
8.58
7.95
7.93
7.32
7.30
2.69
2.67
2.65
1.68
1.66
1.64
1.62
1.60
1.43
1.41
1.39
1.37
1.35
1.34
0.96
0.94
0.93
0.00
N=N
S
n-Bu
3c
1.00
2.00
2.00
2.00
2.05
2.00
3.00
10.5 10.0 9.5 9.0 8.5 8.0 7.5 7.0 6.5 6.0 5.5 5.0 4.5 4.0 3.5 3.0 2.5 2.0 1.5 1.0 0.5 0.0 -0.5 -1.
f1 (ppm)

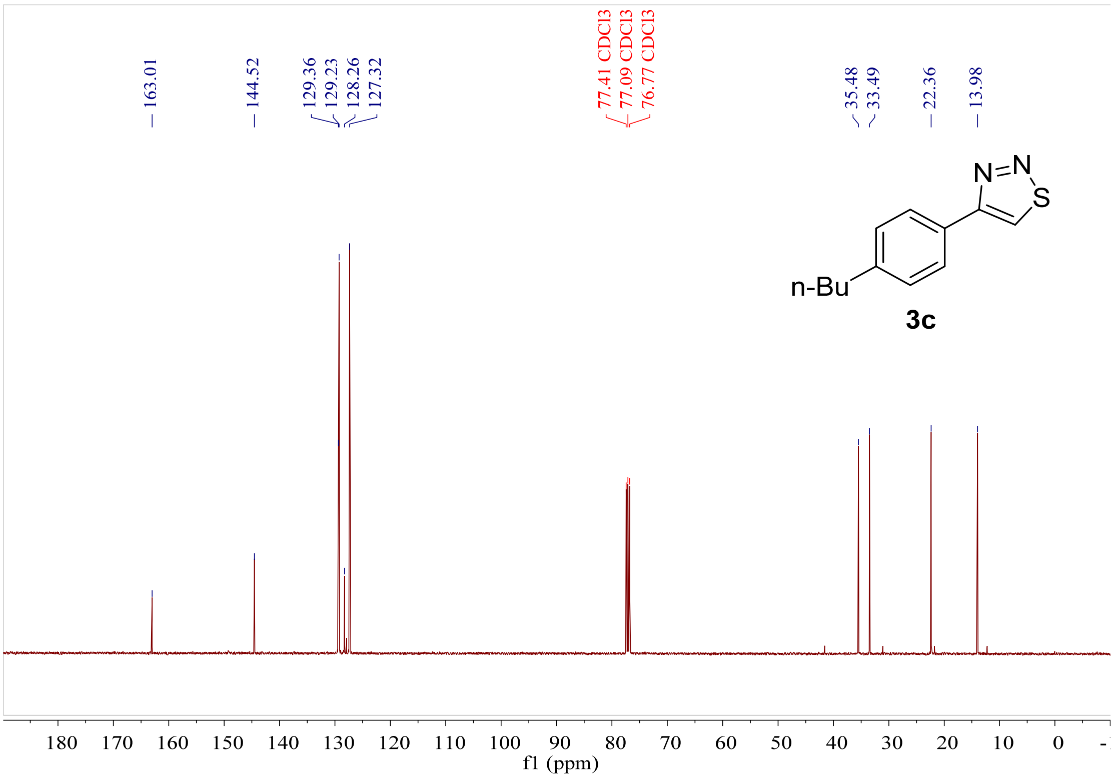
163.01
144.52
129.36
129.23
128.26
127.32
77.41 CDCl3
77.09 CDCl3
76.77 CDCl3
35.48
33.49
22.36
13.98
N=N
S
n-Bu
3c
180 170 160 150 140 130 120 110 100 90 80 70 60 50 40 30 20 10 0
f1 (ppm)

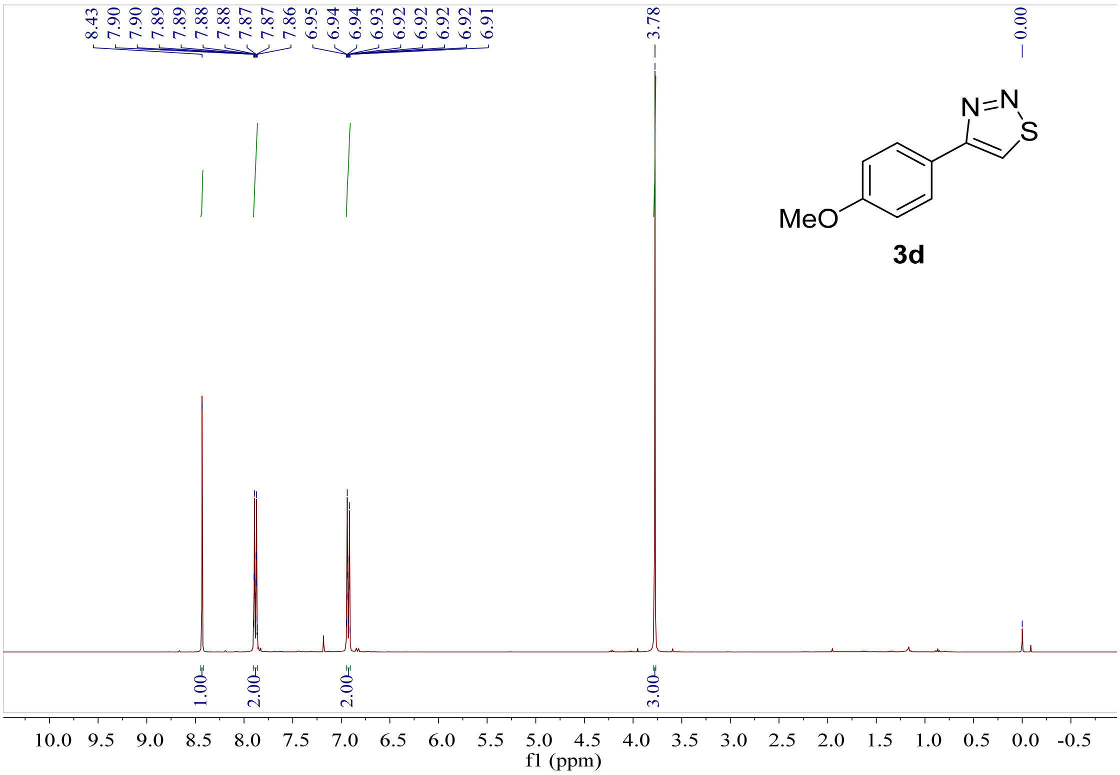
8.43 7.90 7.90 7.89 7.89 7.88 7.88 7.87 7.87 7.86 6.95 6.94 6.94 6.93 6.92 6.92 6.92 6.92 6.92 6.91
3.78
0.00
MeO
N=N
S
3d
1.00
2.00
2.00
3.00
10.0 9.5 9.0 8.5 8.0 7.5 7.0 6.5 6.0 5.5 5.0 4.5 4.0 3.5 3.0 2.5 2.0 1.5 1.0 0.5 0.0 -0.5
f1 (ppm)

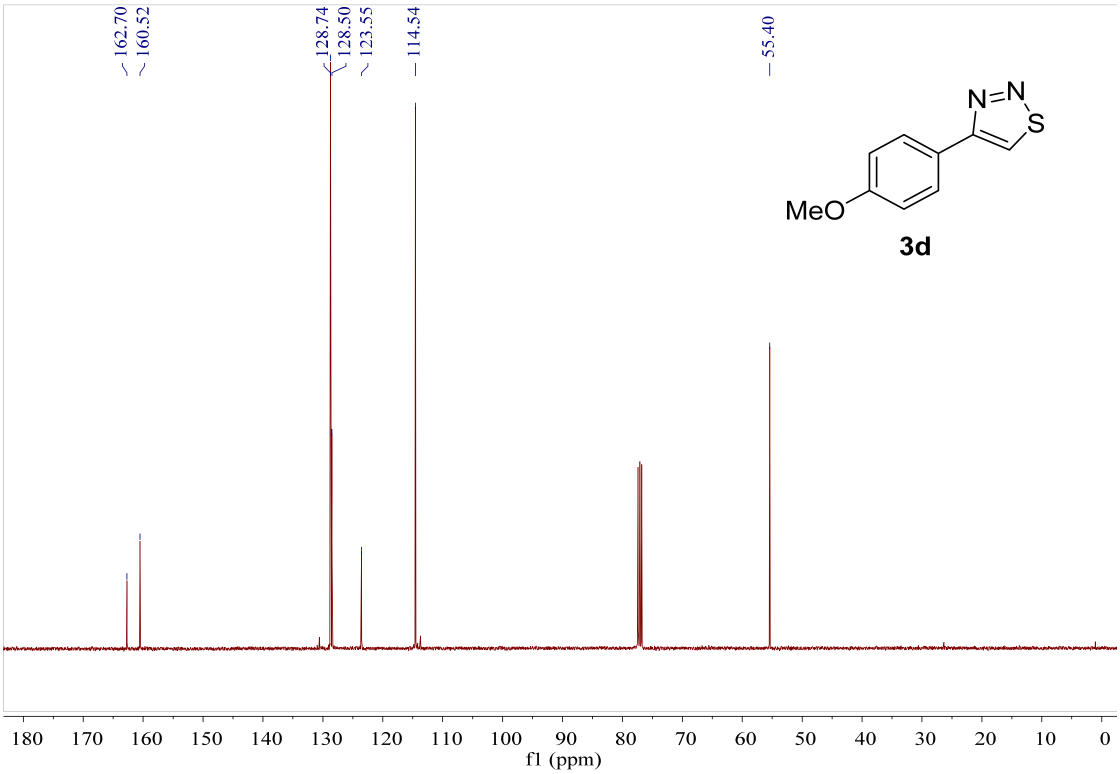
162.70
160.52
128.74
128.50
123.55
114.54
55.40
MeO
N=N
S
3d
180 170 160 150 140 130 120 110 100 90 80 70 60 50 40 30 20 10 0
f1 (ppm)

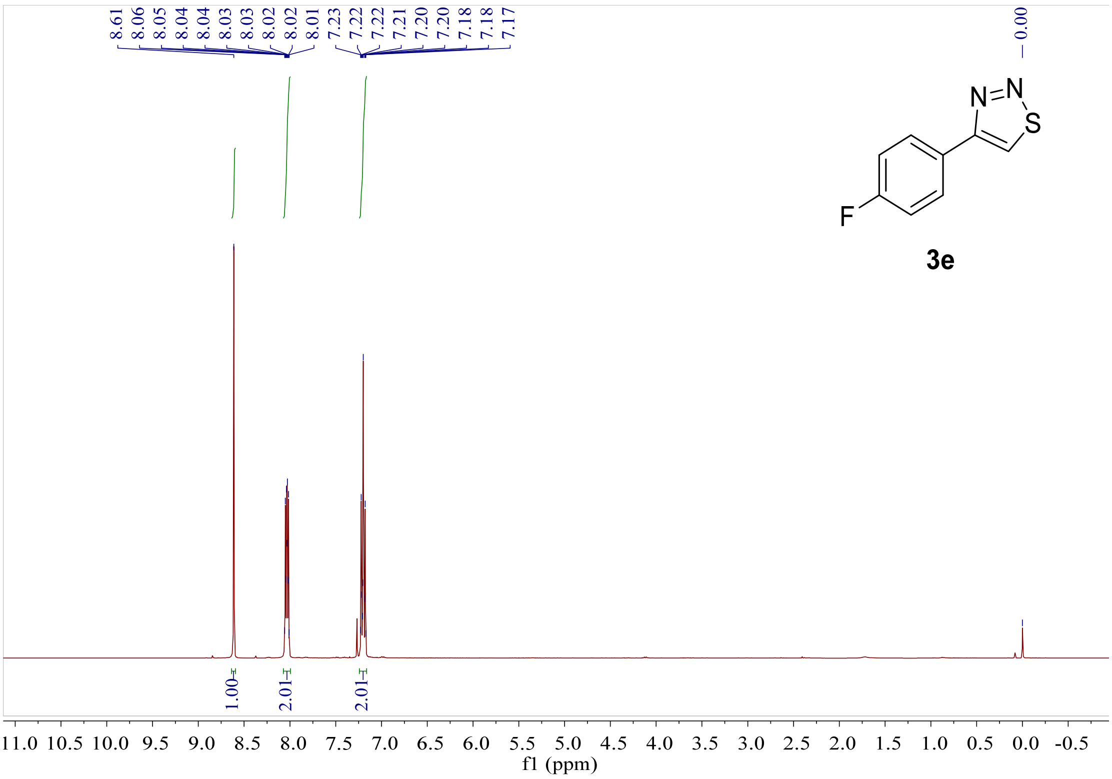
8.61 8.06 8.05 8.04 8.04 8.03 8.03 8.02 8.02 8.01 7.23 7.22 7.22 7.21 7.20 7.20 7.18 7.18 7.17
0.00
N=N
S
F
3e
1.00
2.01
2.01
11.0 10.5 10.0 9.5 9.0 8.5 8.0 7.5 7.0 6.5 6.0 5.5 5.0 4.5 4.0 3.5 3.0 2.5 2.0 1.5 1.0 0.5 0.0 -0.5
f1 (ppm)

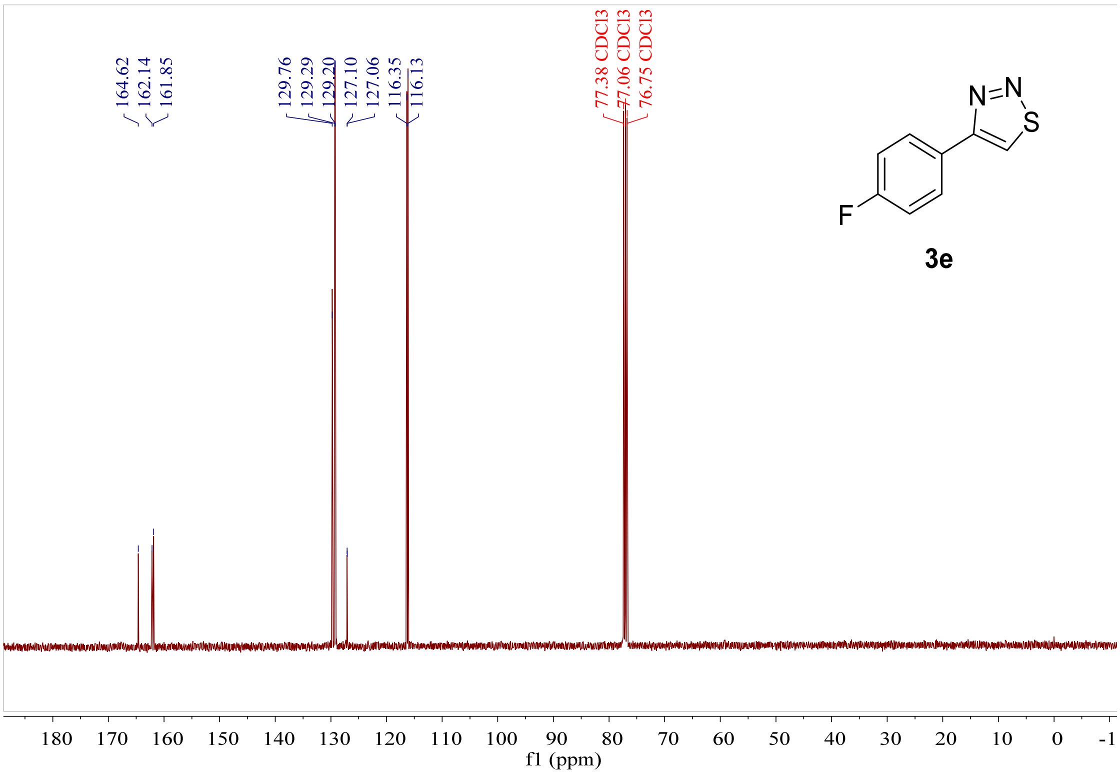
164.62 162.14 161.85
129.76 129.29 129.20 127.10 127.06 116.35 116.13
77.38 CDCl3 77.06 CDCl3 76.75 CDCl3
N=N
S
F
3e
180 170 160 150 140 130 120 110 100 90 80 70 60 50 40 30 20 10 0 -1
f1 (ppm)

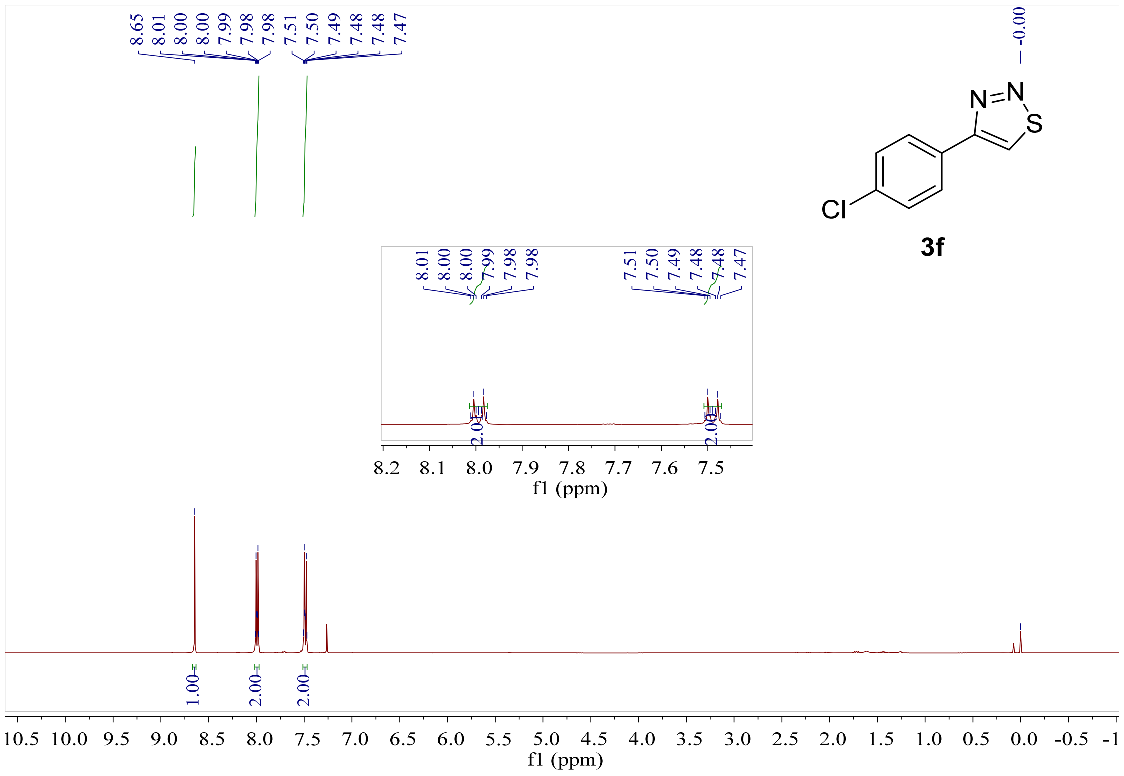

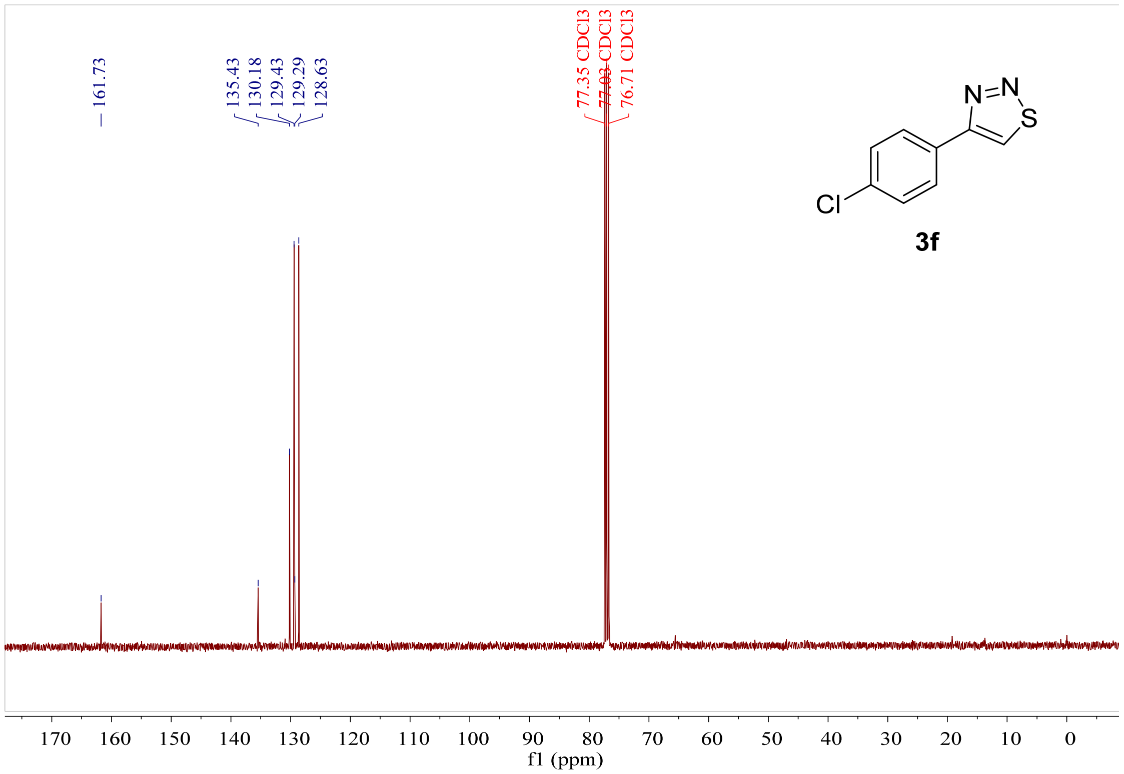

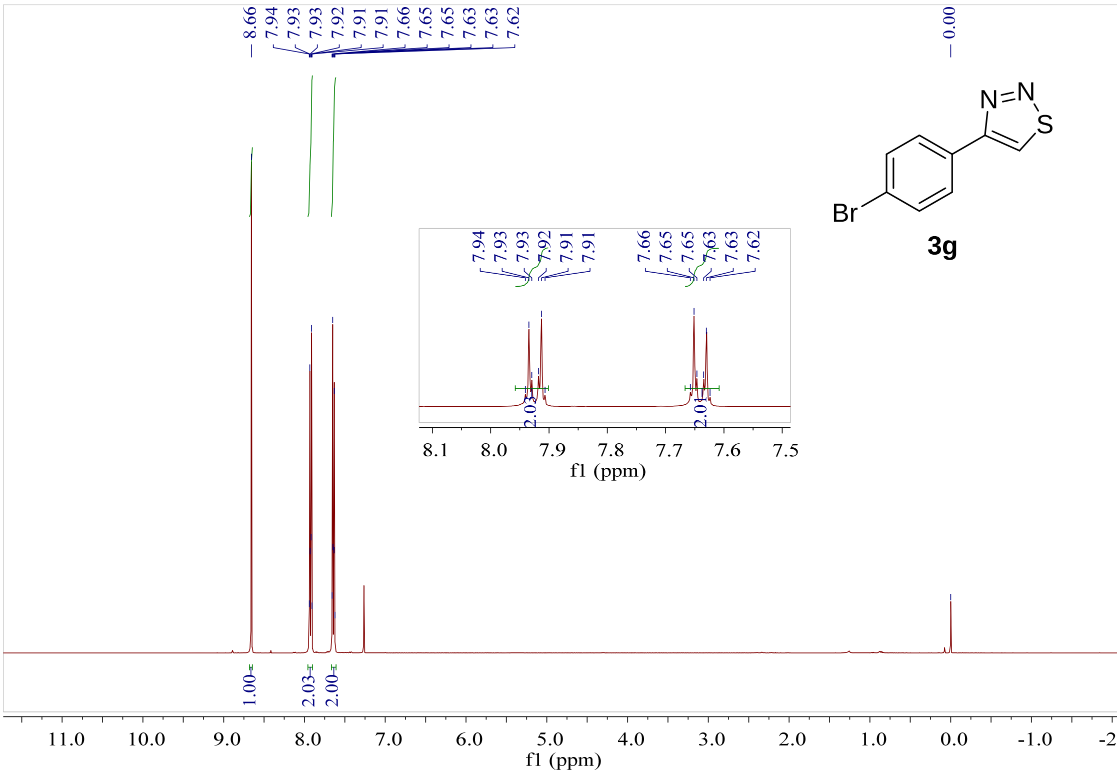
8.66
7.94
7.93
7.93
7.92
7.91
7.91
7.66
7.65
7.65
7.63
7.63
7.62
0.00
N=N
S
Br
3g
1.00
2.03
2.00
2.01
11.0
10.0
9.0
8.0
7.0
6.0
5.0
4.0
3.0
2.0
1.0
0.0
-1.0
-2
f1 (ppm)
8.1
7.9
7.8
7.7
7.6
7.5

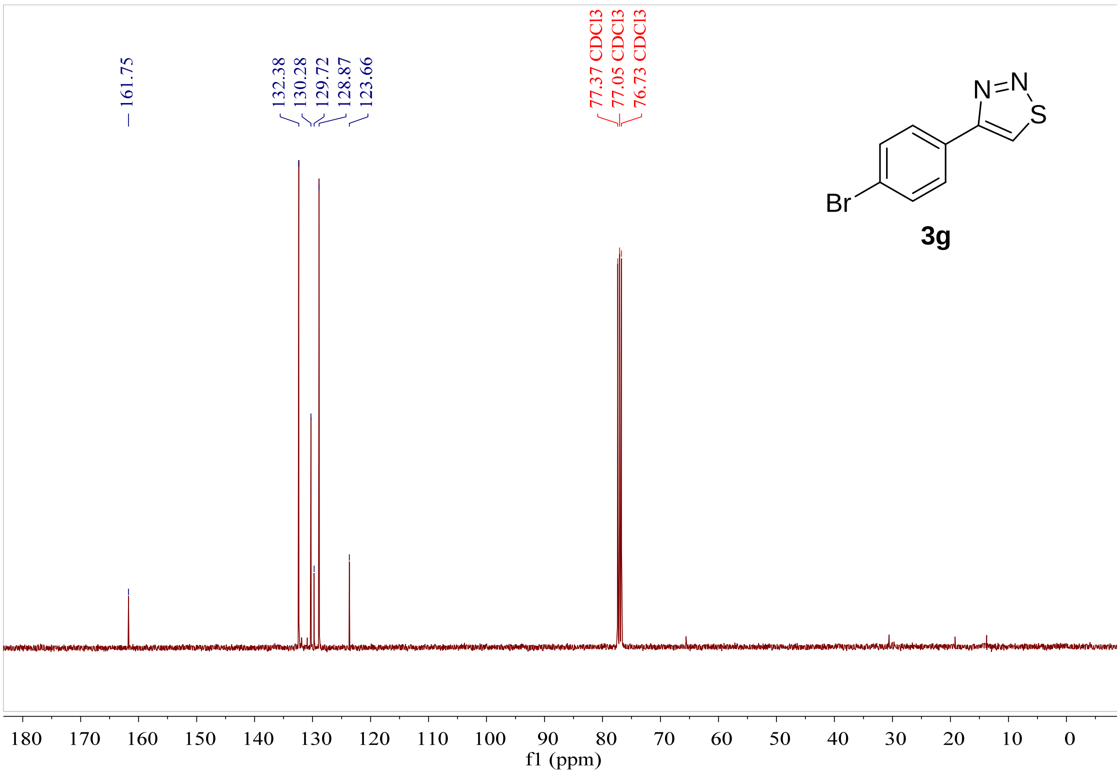
161.75
132.38
130.28
129.72
128.87
123.66
77.37 CDCl3
77.05 CDCl3
76.73 CDCl3
N=N
S
Br
3g
180
170
160
150
140
130
120
110
100
90
80
70
60
50
40
30
20
10
0
f1 (ppm)

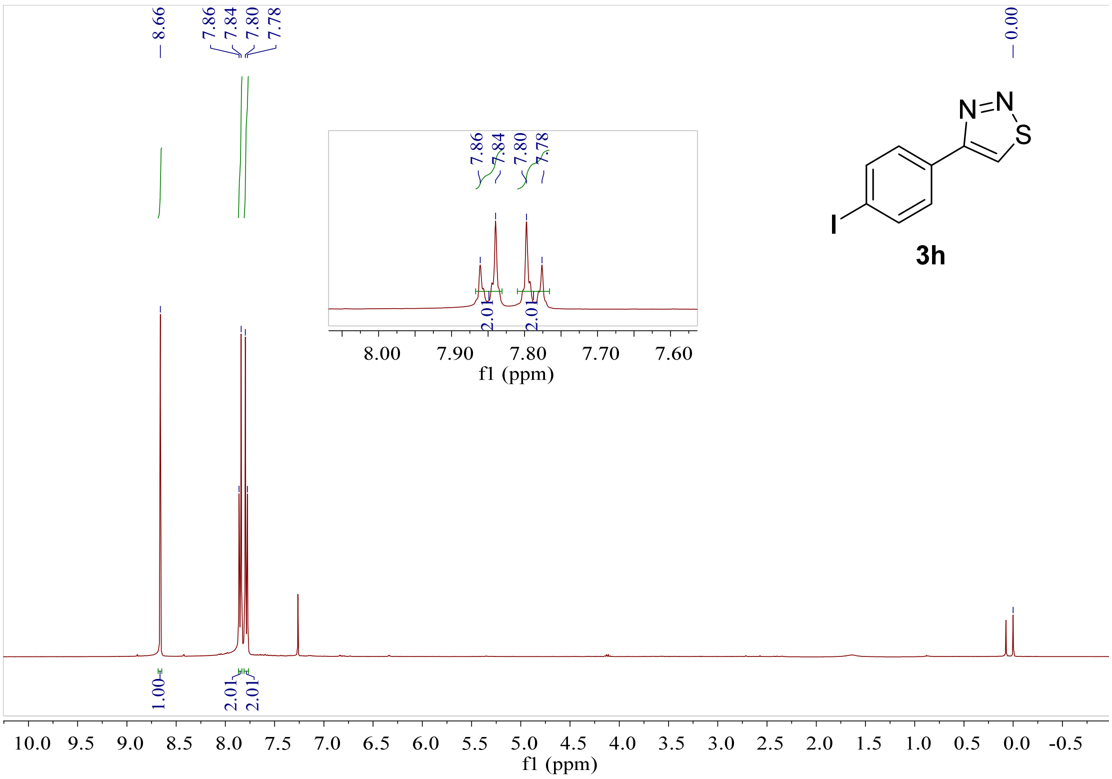
8.66
7.86
7.84
7.80
7.78
0.00
1.00
2.01
2.01
8.00 7.90 7.80 7.70 7.60
f1 (ppm)
N=N
S
I
3h
10.0 9.5 9.0 8.5 8.0 7.5 7.0 6.5 6.0 5.5 5.0 4.5 4.0 3.5 3.0 2.5 2.0 1.5 1.0 0.5 0.0 -0.5
f1 (ppm)

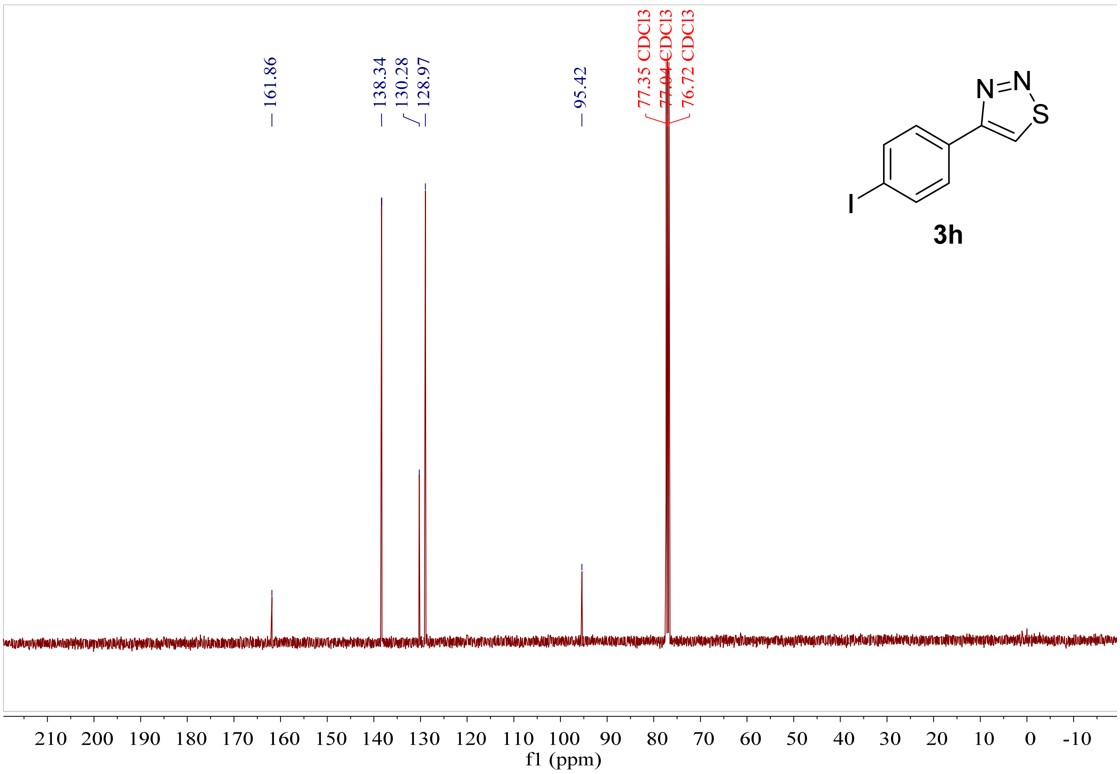
161.86
138.34
130.28
128.97
95.42
77.35 CDCl3
77.04 CDCl3
76.72 CDCl3
N=N
S
I
3h
210 200 190 180 170 160 150 140 130 120 110 100 90 80 70 60 50 40 30 20 10 0 -10
f1 (ppm)

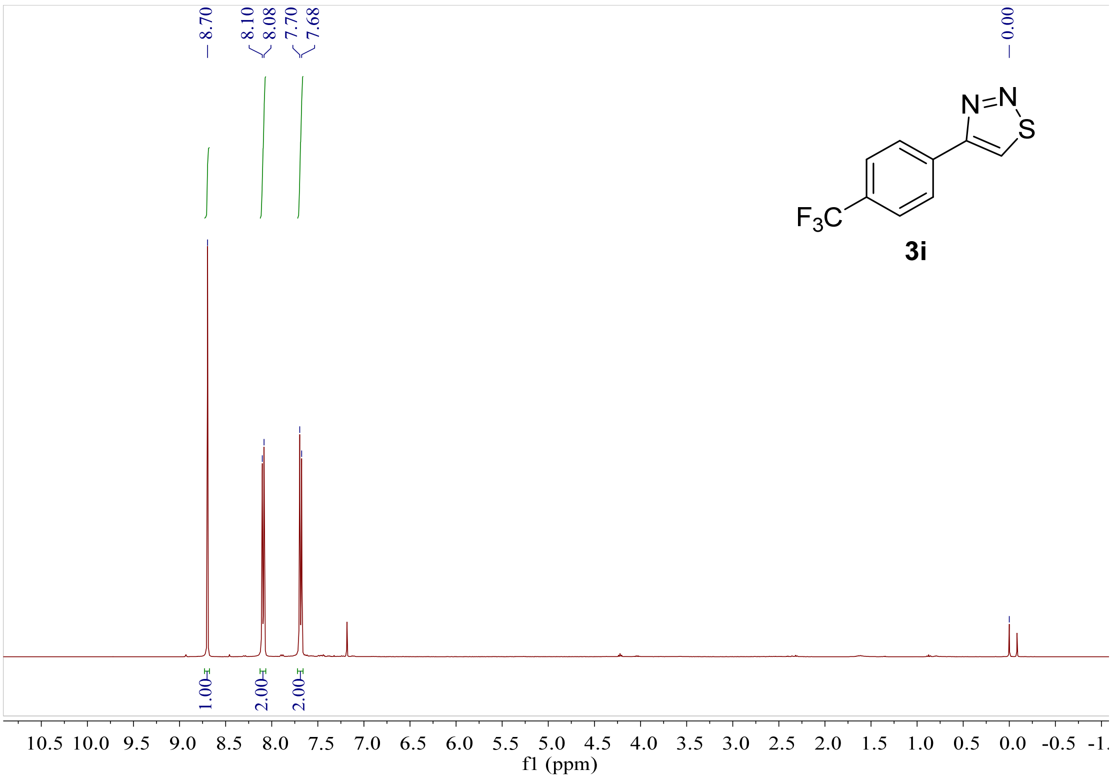

8.70
8.10
8.08
7.70
7.68
0.00
1.00
2.00
2.00
10.5 10.0 9.5 9.0 8.5 8.0 7.5 7.0 6.5 6.0 5.5 5.0 4.5 4.0 3.5 3.0 2.5 2.0 1.5 1.0 0.5 0.0 -0.5 -1.
f1 (ppm)
N=N
S
F3C
3i

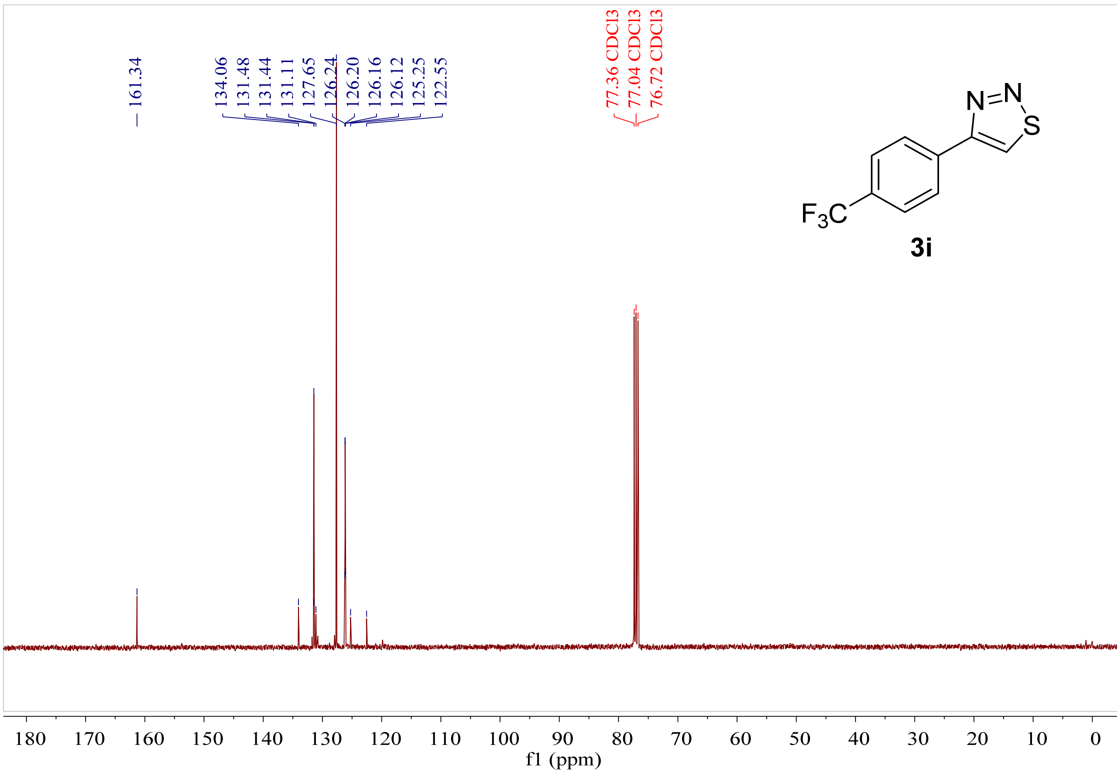

161.34
134.06
131.48
131.44
131.11
127.65
126.24
126.20
126.16
126.12
125.25
122.55
77.36 CDCl3
77.04 CDCl3
76.72 CDCl3
180 170 160 150 140 130 120 110 100 90 80 70 60 50 40 30 20 10 0
f1 (ppm)
N=N
S
F3C
3i

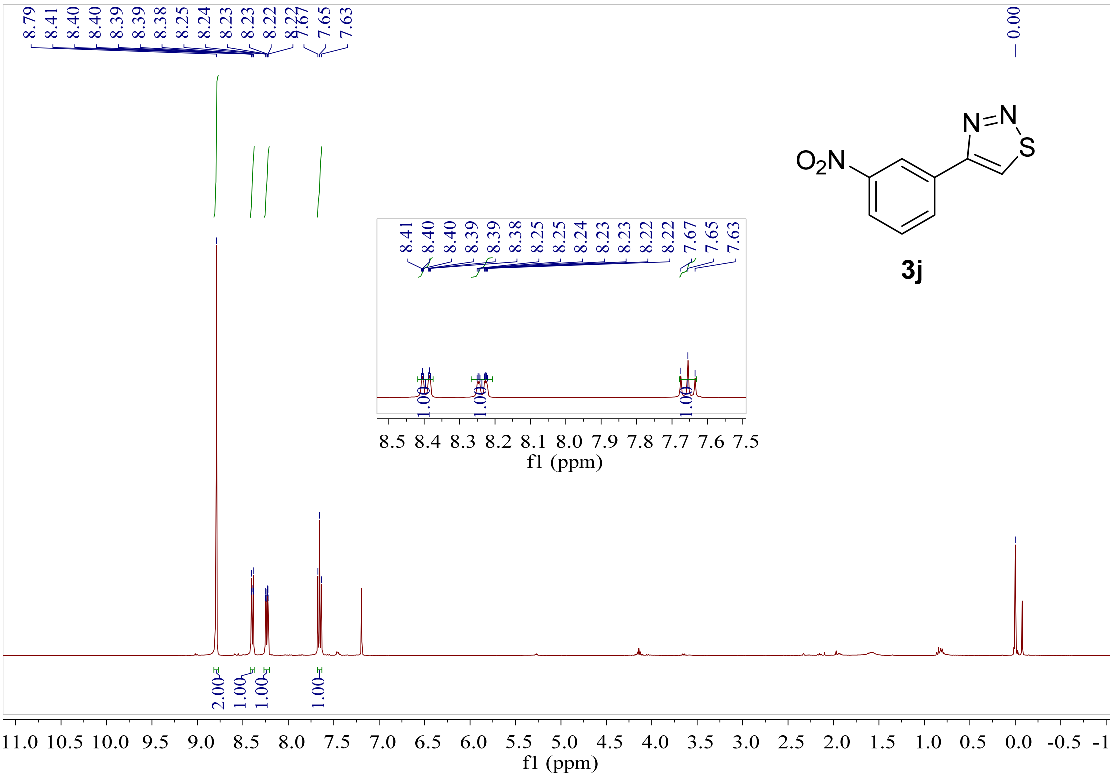

8.79
8.41
8.40
8.40
8.39
8.39
8.38
8.25
8.24
8.23
8.23
8.22
8.22
7.67
7.65
7.63
0.00
O2N
N=N
S
3j
2.00
1.00
1.00
1.00
11.0 10.5 10.0 9.5 9.0 8.5 8.0 7.5 7.0 6.5 6.0 5.5 5.0 4.5 4.0 3.5 3.0 2.5 2.0 1.5 1.0 0.5 0.0 -0.5 -1
f1 (ppm)
8.5 8.4 8.3 8.2 8.1 8.0 7.9 7.8 7.7 7.6 7.5
f1 (ppm)

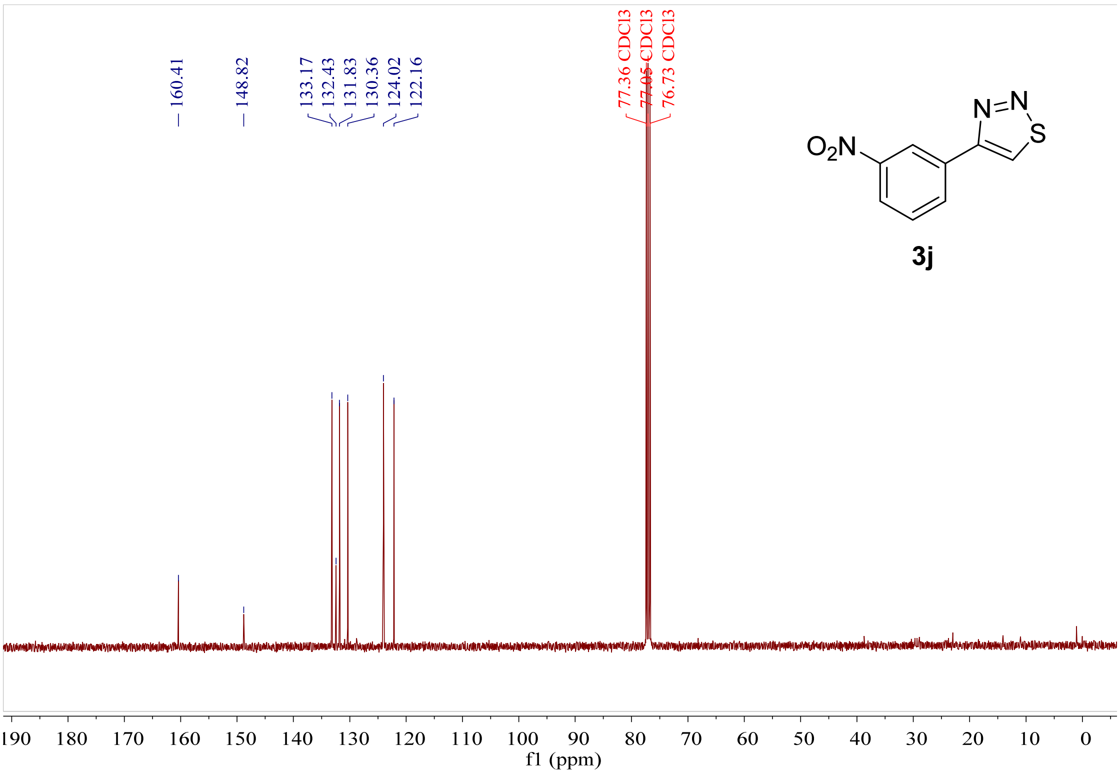

160.41
148.82
133.17
132.43
131.83
130.36
124.02
122.16
77.36 CDCl3
77.05 CDCl3
76.73 CDCl3
O2N
N=N
S
3j
190 180 170 160 150 140 130 120 110 100 90 80 70 60 50 40 30 20 10 0
f1 (ppm)

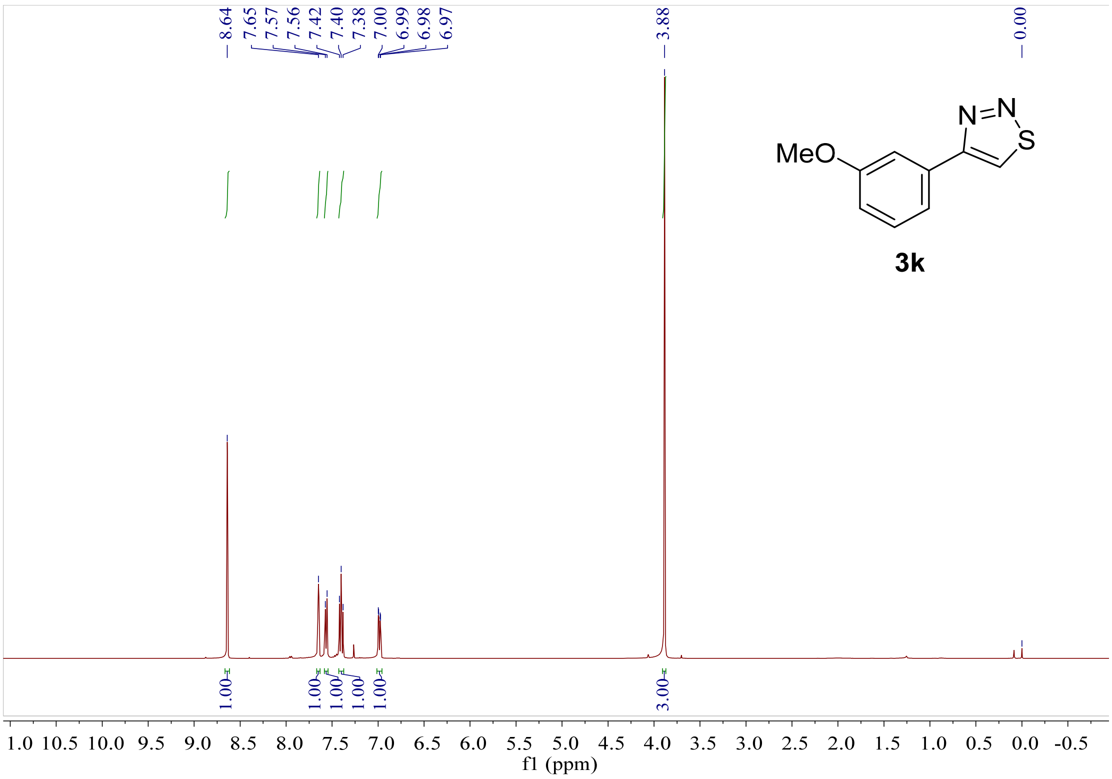
8.64
7.65
7.57
7.56
7.42
7.40
7.38
7.00
6.99
6.98
6.97
3.88
0.00
MeO
N=N
S
3k
1.00
1.00
1.00
1.00
1.00
3.00
1.0 10.5 10.0 9.5 9.0 8.5 8.0 7.5 7.0 6.5 6.0 5.5 5.0 4.5 4.0 3.5 3.0 2.5 2.0 1.5 1.0 0.5 0.0 -0.5
f1 (ppm)

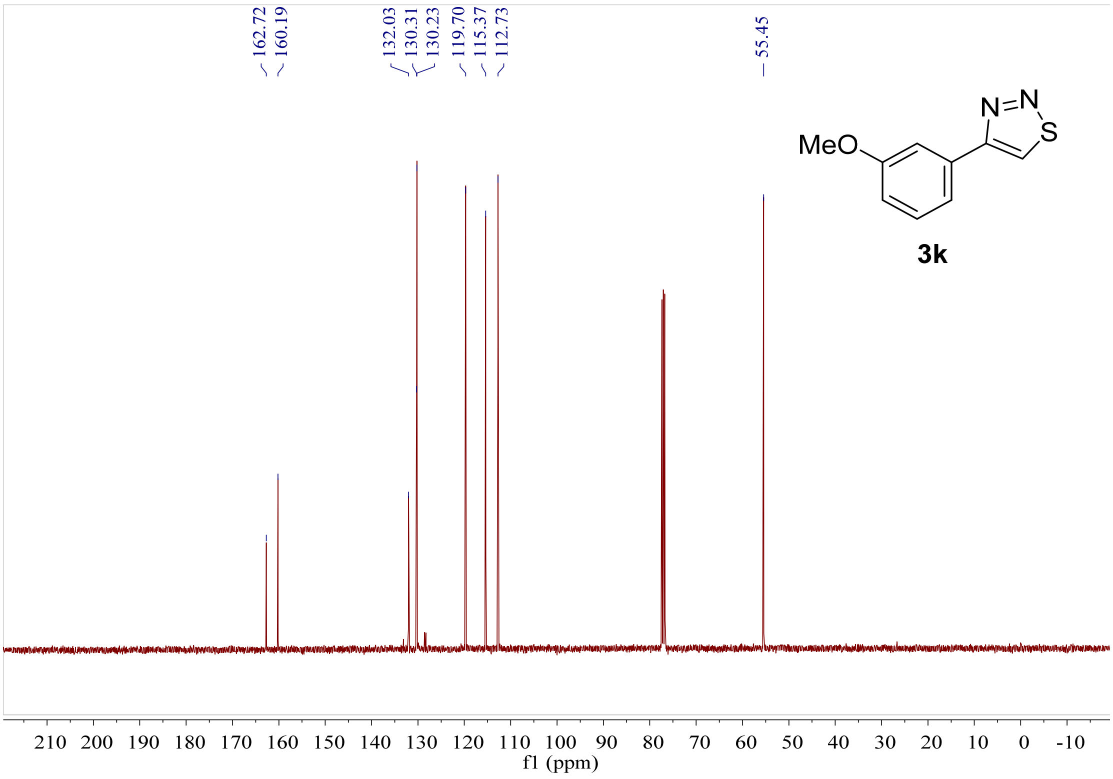
162.72
160.19
132.03
130.31
130.23
119.70
115.37
112.73
55.45
MeO
N=N
S
3k
210 200 190 180 170 160 150 140 130 120 110 100 90 80 70 60 50 40 30 20 10 0 -10
f1 (ppm)

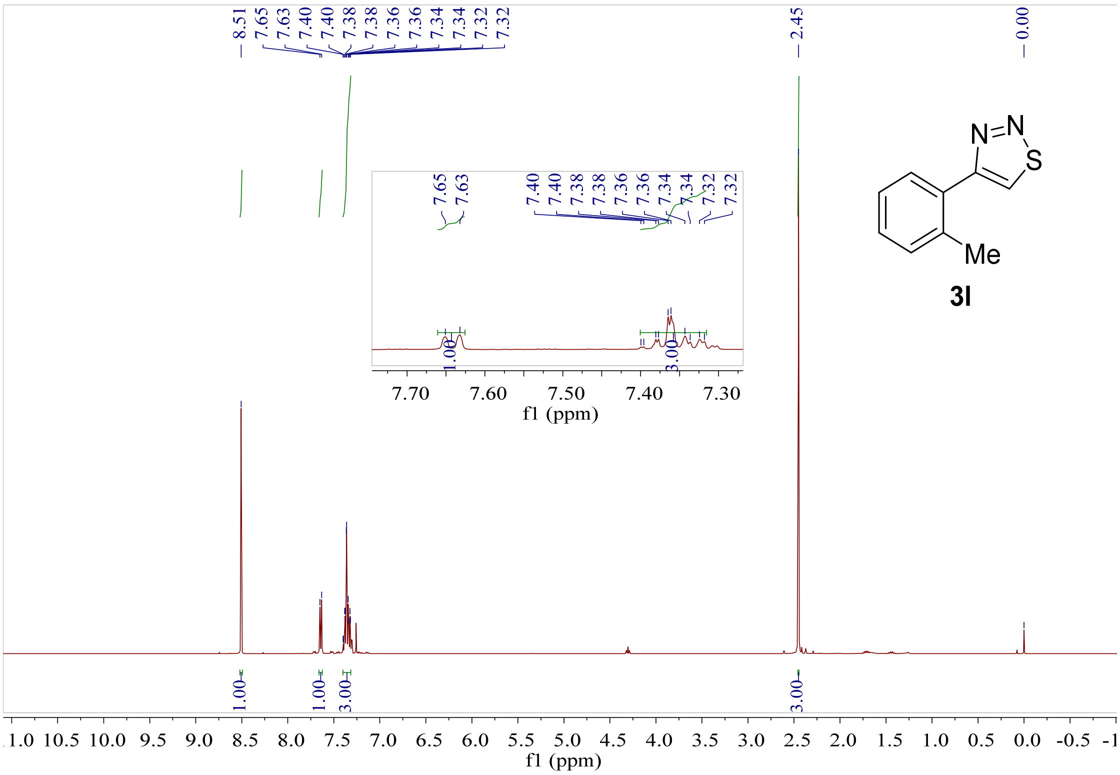
8.51
7.65
7.63
7.40
7.40
7.38
7.38
7.36
7.36
7.34
7.34
7.32
7.32
2.45
0.00
1.00
1.00
3.00
3.00
7.70
7.60
7.50
7.40
7.30
f1 (ppm)
N=N
S
Me
3l
11.0 10.5 10.0 9.5 9.0 8.5 8.0 7.5 7.0 6.5 6.0 5.5 5.0 4.5 4.0 3.5 3.0 2.5 2.0 1.5 1.0 0.5 0.0 -0.5 -1
f1 (ppm)

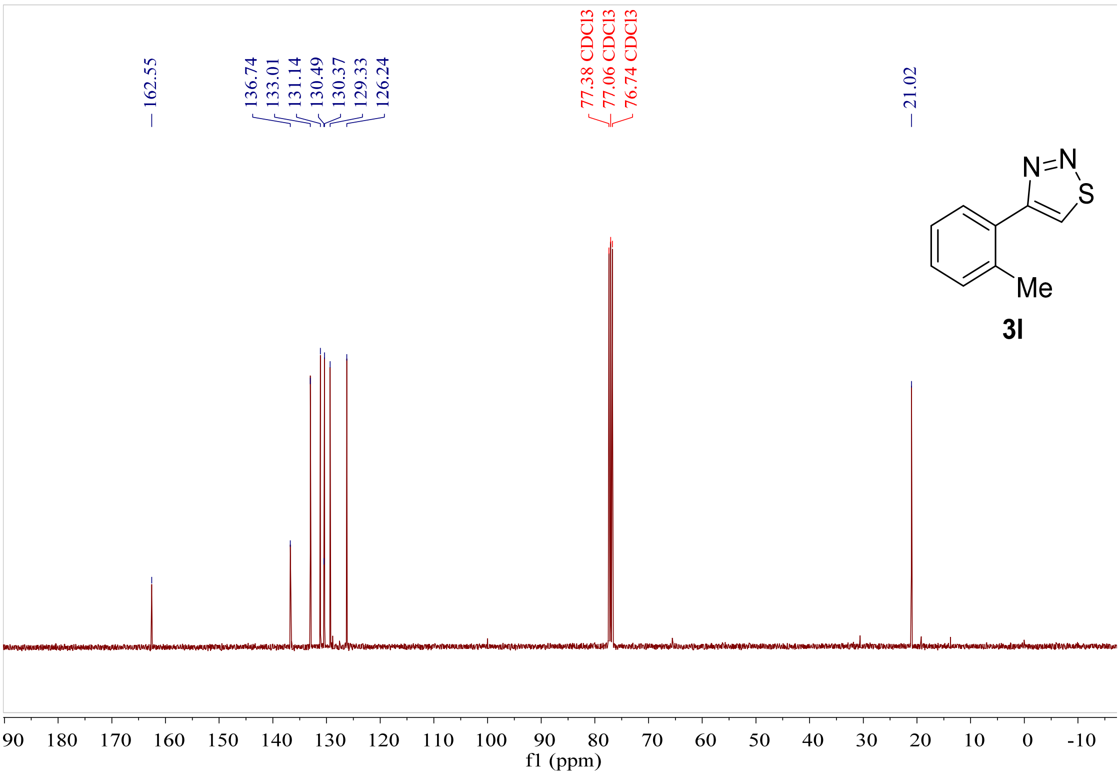
162.55
136.74
133.01
131.14
130.49
130.37
129.33
126.24
77.38 CDCl3
77.06 CDCl3
76.74 CDCl3
21.02
N=N
S
Me
3l
190 180 170 160 150 140 130 120 110 100 90 80 70 60 50 40 30 20 10 0 -10
f1 (ppm)

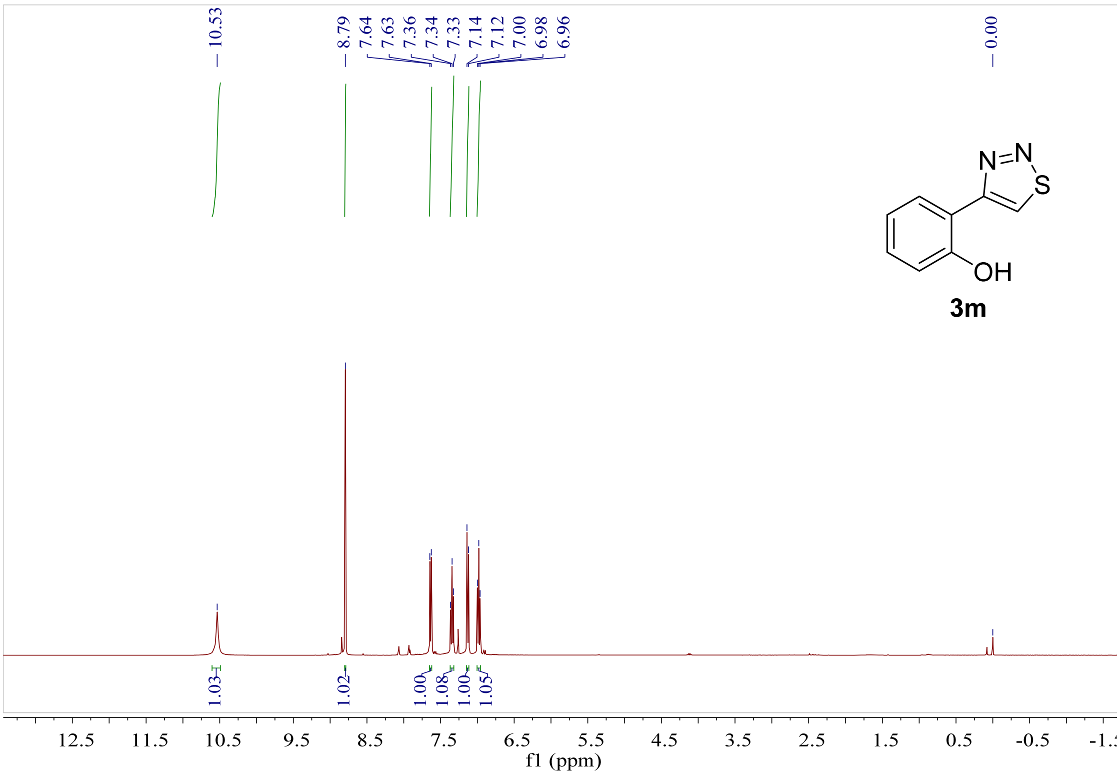
10.53
8.79
7.64
7.63
7.36
7.34
7.33
7.14
7.12
7.00
6.98
6.96
0.00
N=N
S
OH
3m
1.03
1.02
1.00
1.08
1.00
1.05
12.5
11.5
10.5
9.5
8.5
7.5
6.5
5.5
4.5
3.5
2.5
1.5
0.5
-0.5
f1 (ppm)

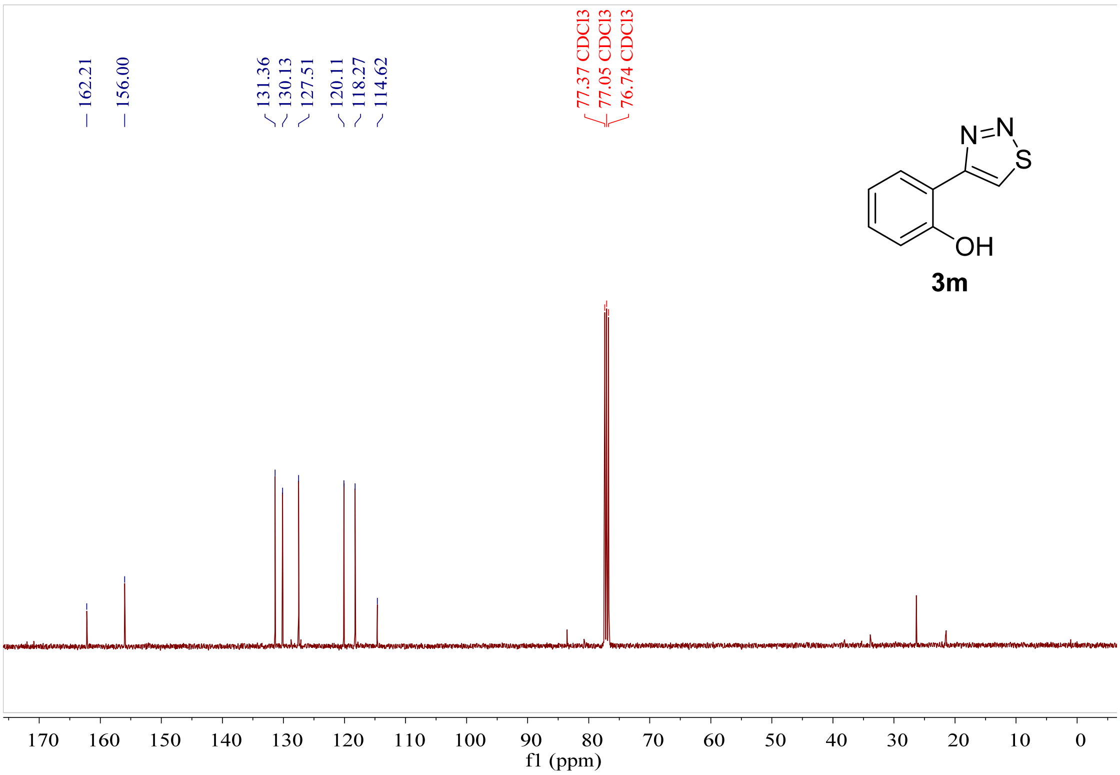
162.21
156.00
131.36
130.13
127.51
120.11
118.27
114.62
77.37 CDCl3
77.05 CDCl3
76.74 CDCl3
N=N
S
OH
3m
170
160
150
140
130
120
110
100
90
80
70
60
50
40
30
20
10
0
f1 (ppm)

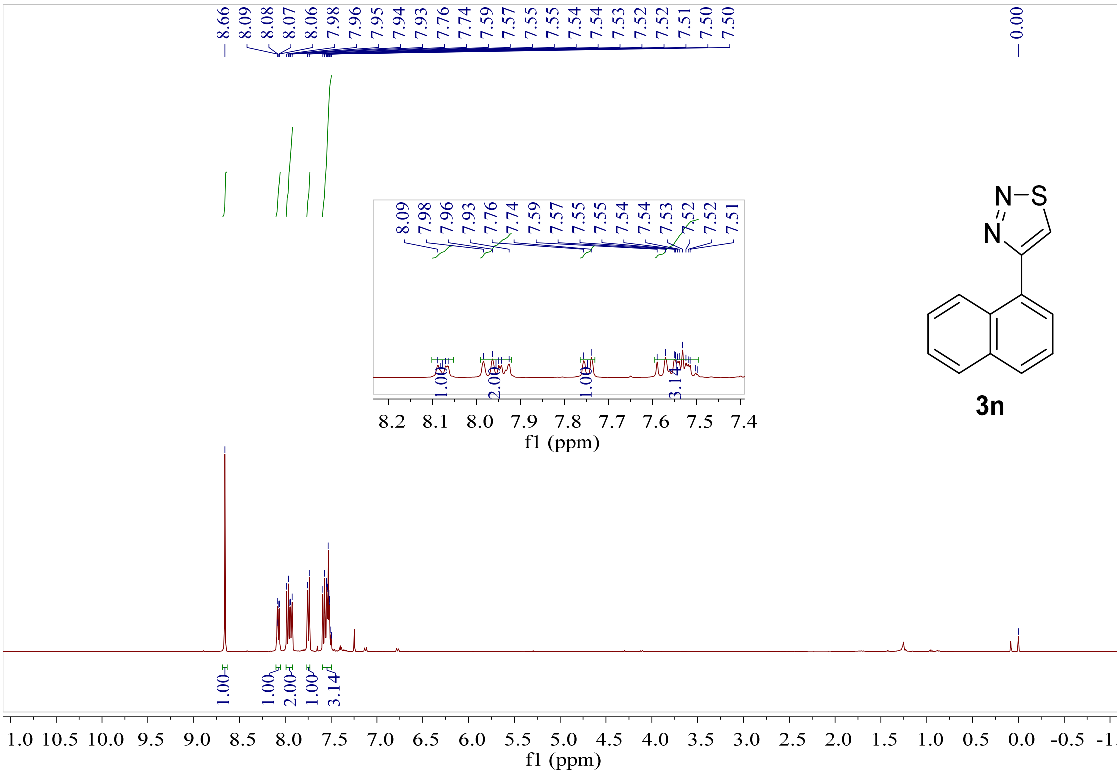
8.66
8.09
8.08
8.07
8.06
7.98
7.96
7.95
7.94
7.93
7.76
7.74
7.59
7.57
7.55
7.55
7.54
7.54
7.53
7.52
7.52
7.51
7.50
7.50
0.00
1.00
1.00
2.00
1.00
3.14
f1 (ppm)
N-S
N
3n

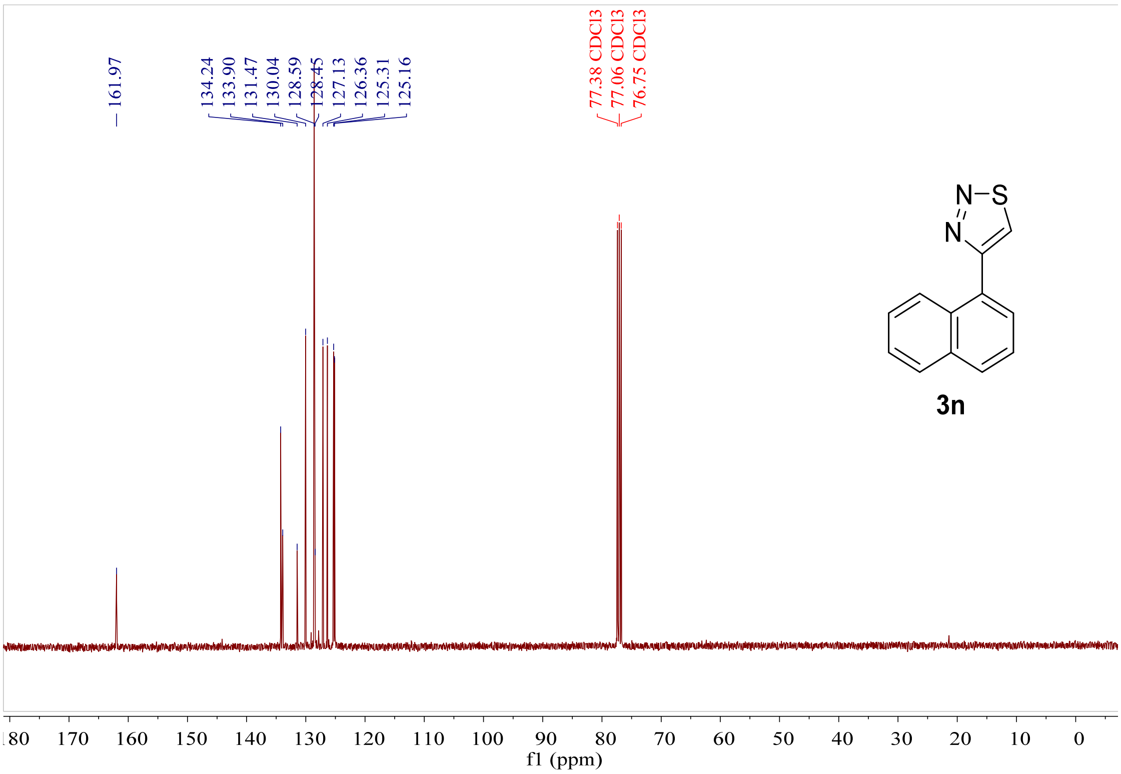
161.97
134.24
133.90
131.47
130.04
128.59
128.45
127.13
126.36
125.31
125.16
77.38 CDCl3
77.06 CDCl3
76.75 CDCl3
f1 (ppm)
N-S
N
3n

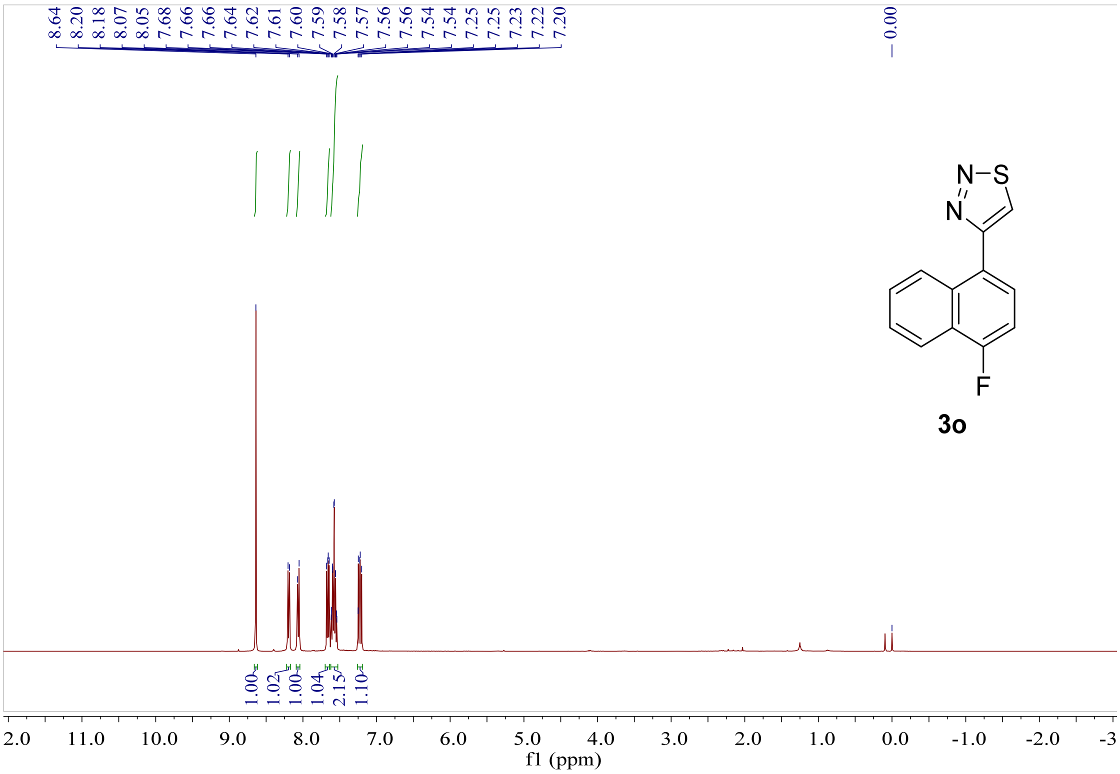
8.64
8.20
8.18
8.07
8.05
7.68
7.66
7.66
7.64
7.62
7.61
7.60
7.59
7.58
7.57
7.56
7.56
7.54
7.54
7.25
7.25
7.23
7.22
7.20
0.00
N–S
N
F
3o
1.00
1.02
1.00
1.04
2.15
1.10
f1 (ppm)

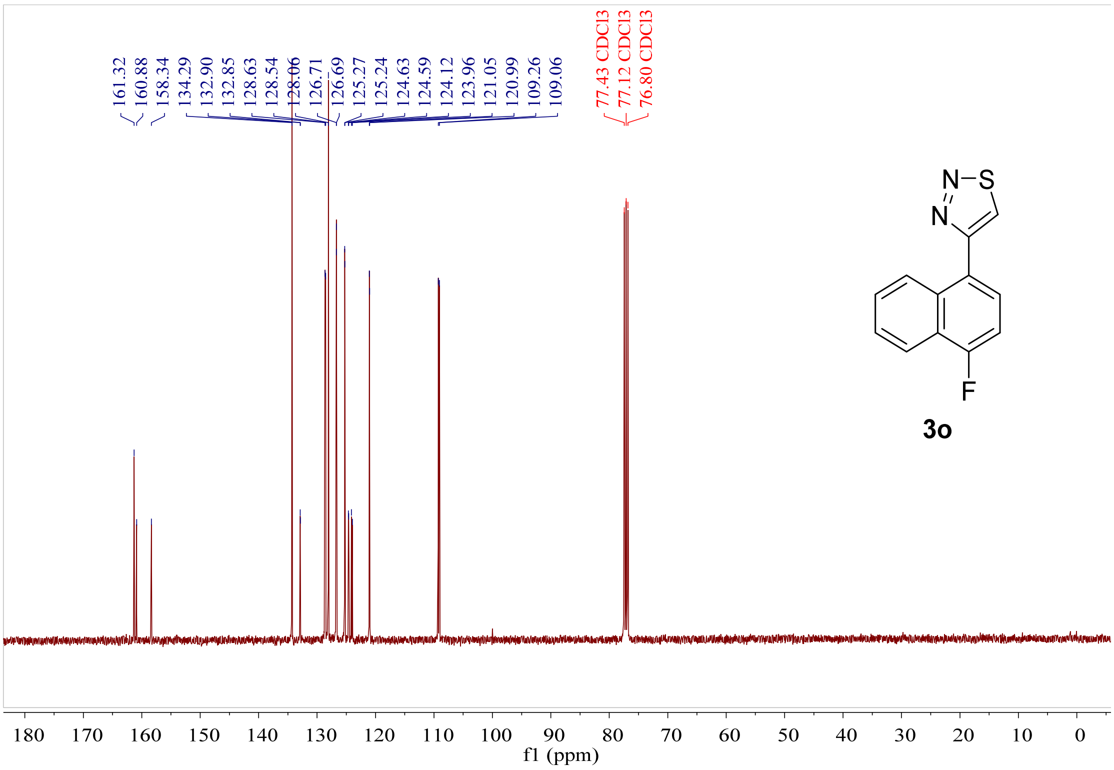
161.32
160.88
158.34
134.29
132.90
132.85
128.63
128.54
128.06
126.71
126.69
125.27
125.24
124.63
124.59
124.12
123.96
121.05
120.99
109.26
109.06
77.43 CDCl3
77.12 CDCl3
76.80 CDCl3
N–S
N
F
3o
f1 (ppm)

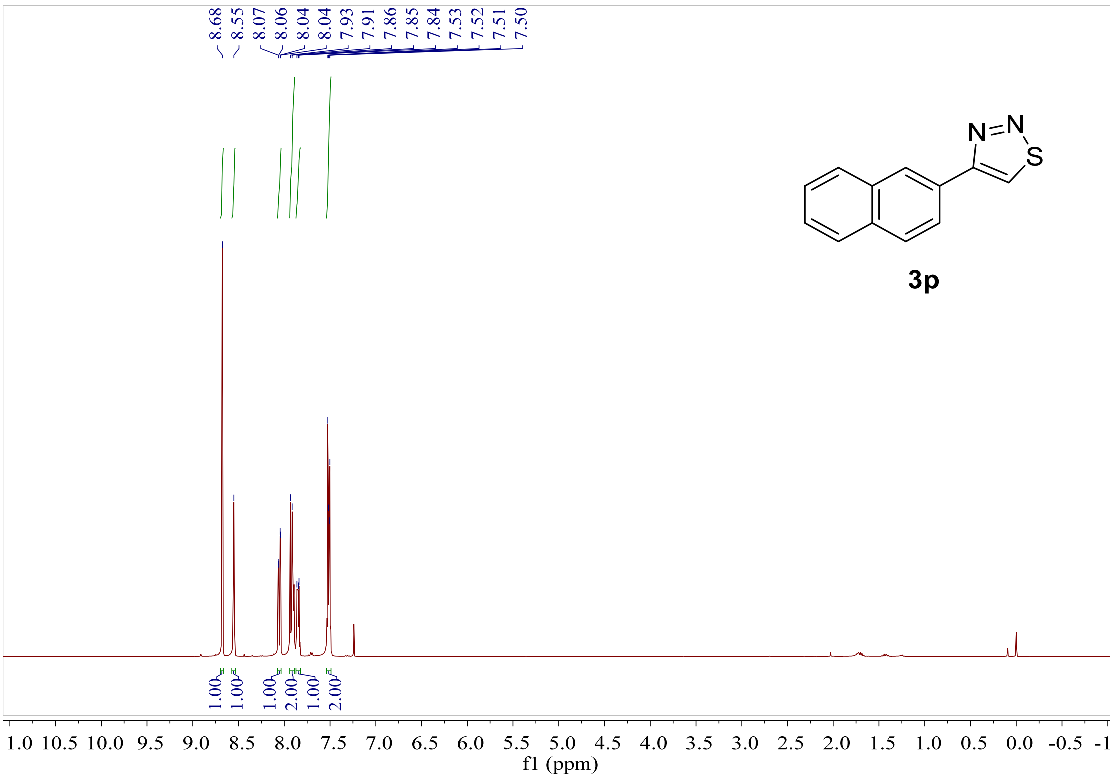
8.68
8.55
8.07
8.06
8.04
8.04
7.93
7.91
7.86
7.85
7.84
7.53
7.52
7.51
7.50
1.00
1.00
1.00
2.00
1.00
2.00
N=N
S
3p
1.0 10.5 10.0 9.5 9.0 8.5 8.0 7.5 7.0 6.5 6.0 5.5 5.0 4.5 4.0 3.5 3.0 2.5 2.0 1.5 1.0 0.5 0.0 -0.5 -1
f1 (ppm)

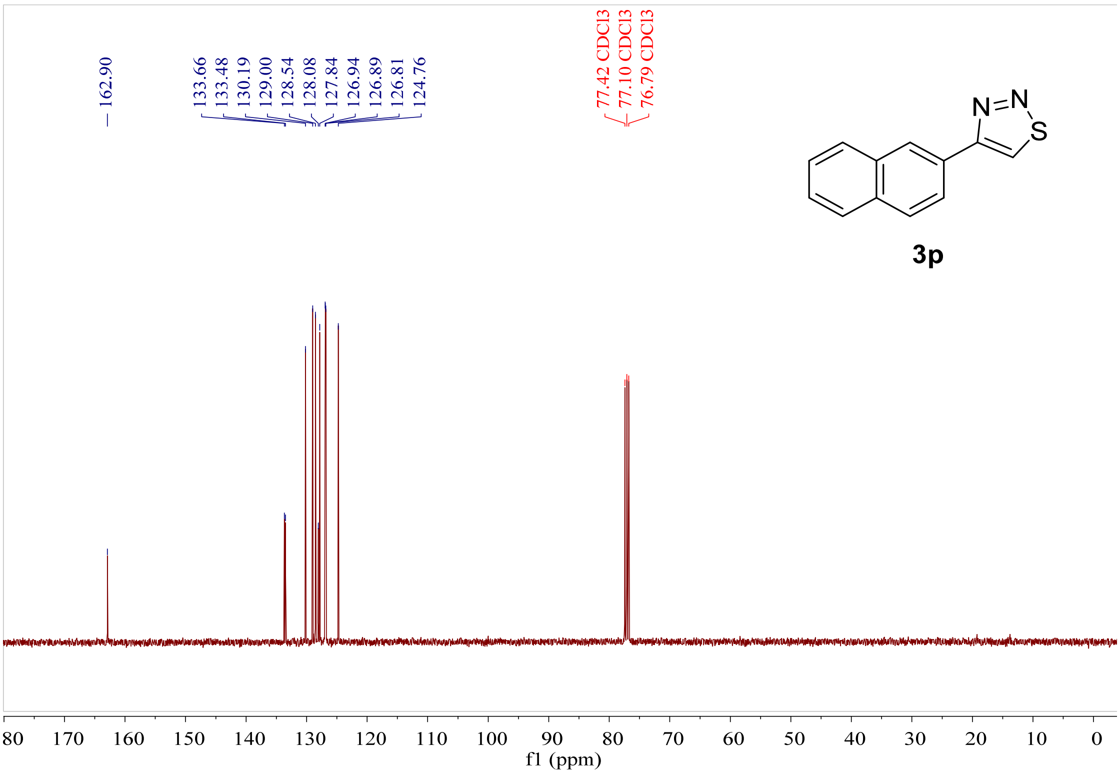
162.90
133.66
133.48
130.19
129.00
128.54
128.08
127.84
126.94
126.89
126.81
124.76
77.42 CDCl3
77.10 CDCl3
76.79 CDCl3
N=N
S
3p
80 170 160 150 140 130 120 110 100 90 80 70 60 50 40 30 20 10 0
f1 (ppm)

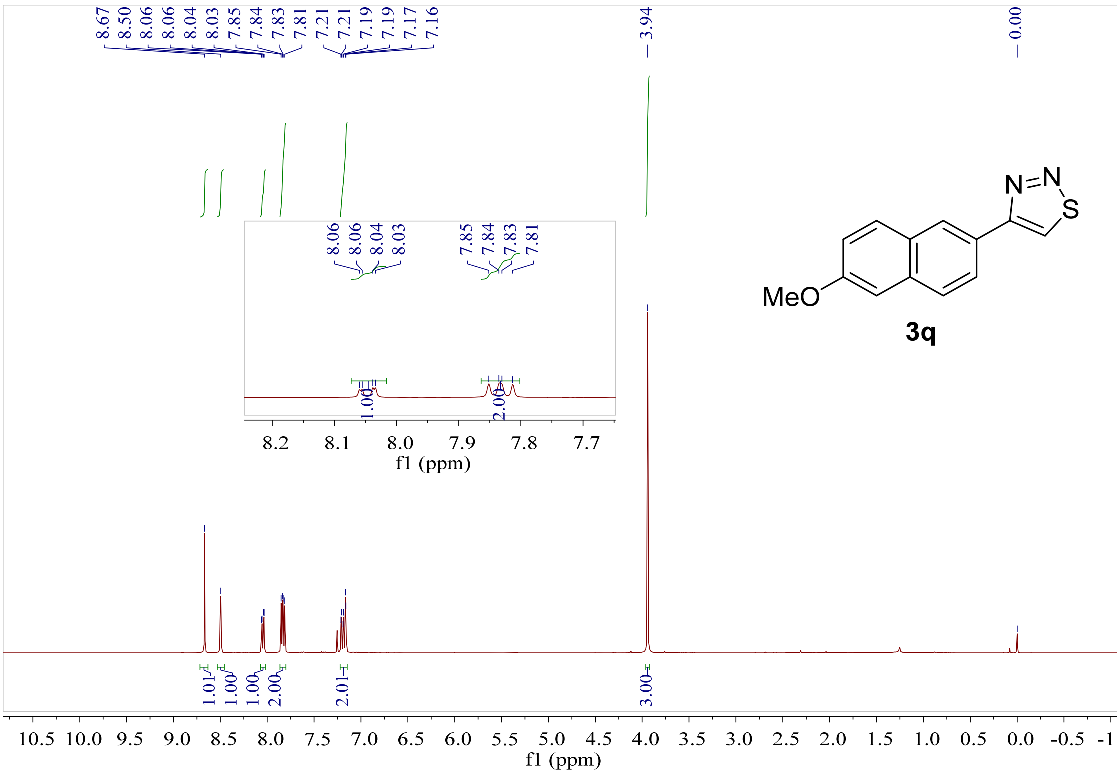
8.67
8.50
8.06
8.06
8.04
8.03
7.85
7.84
7.83
7.81
7.21
7.21
7.19
7.19
7.17
7.16
3.94
0.00
N=N
S
MeO
3q
1.01
1.00
1.00
2.00
2.01
3.00
10.5 10.0 9.5 9.0 8.5 8.0 7.5 7.0 6.5 6.0 5.5 5.0 4.5 4.0 3.5 3.0 2.5 2.0 1.5 1.0 0.5 0.0 -0.5 -1
f1 (ppm)
8.2 8.1 8.0 7.9 7.8 7.7
f1 (ppm)

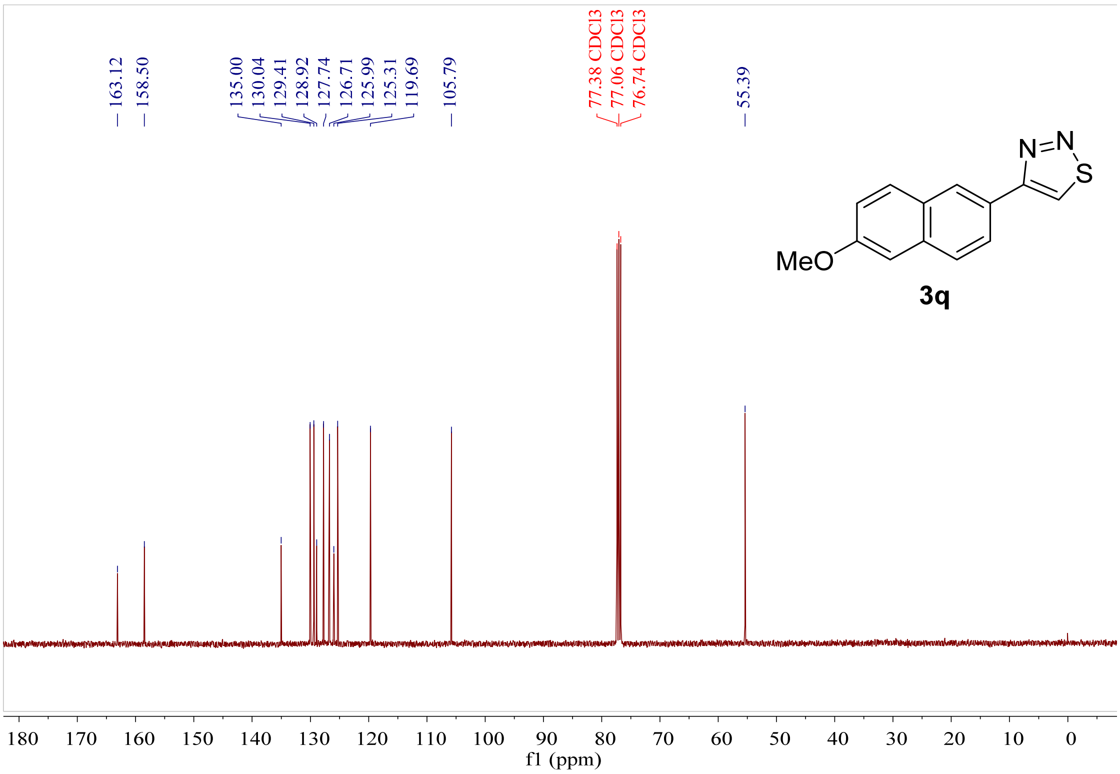
163.12
158.50
135.00
130.04
129.41
128.92
127.74
126.71
125.99
125.31
119.69
105.79
77.38 CDCl3
77.06 CDCl3
76.74 CDCl3
55.39
N=N
S
MeO
3q
180 170 160 150 140 130 120 110 100 90 80 70 60 50 40 30 20 10 0
f1 (ppm)

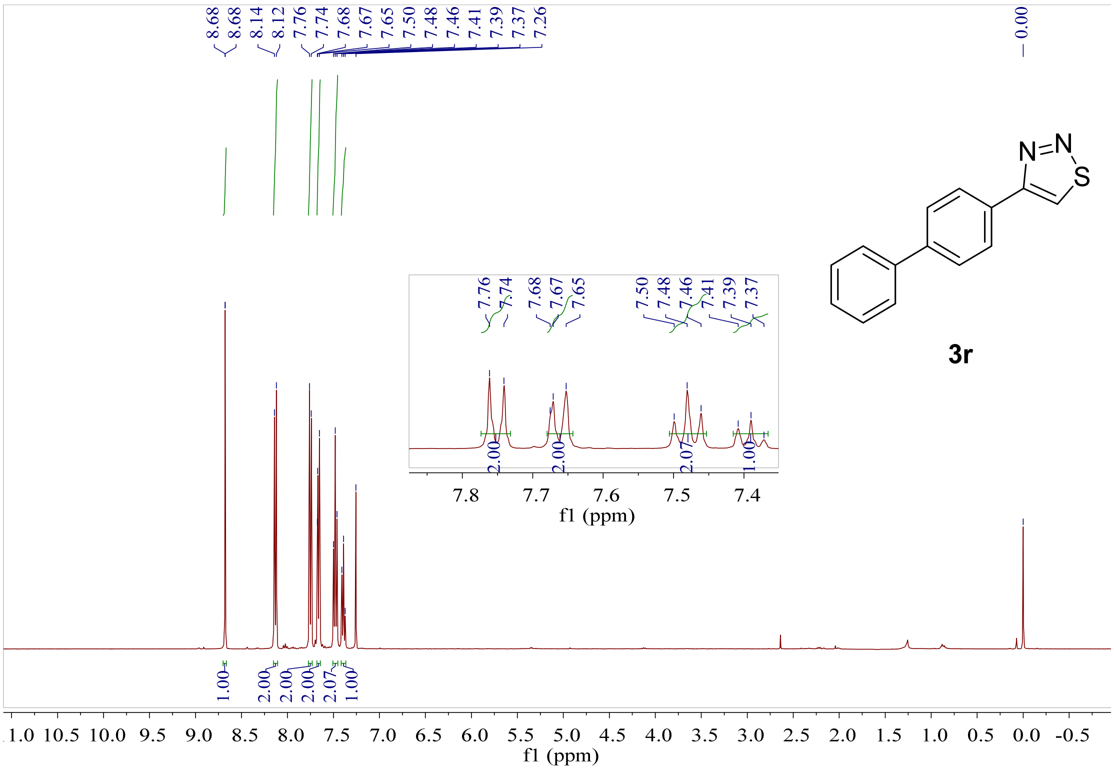
8.68
8.68
8.14
8.12
7.76
7.74
7.68
7.67
7.65
7.50
7.48
7.46
7.41
7.39
7.37
7.26
0.00
N=N
S
3r
1.00
2.00
2.00
2.00
2.07
1.00
f1 (ppm)

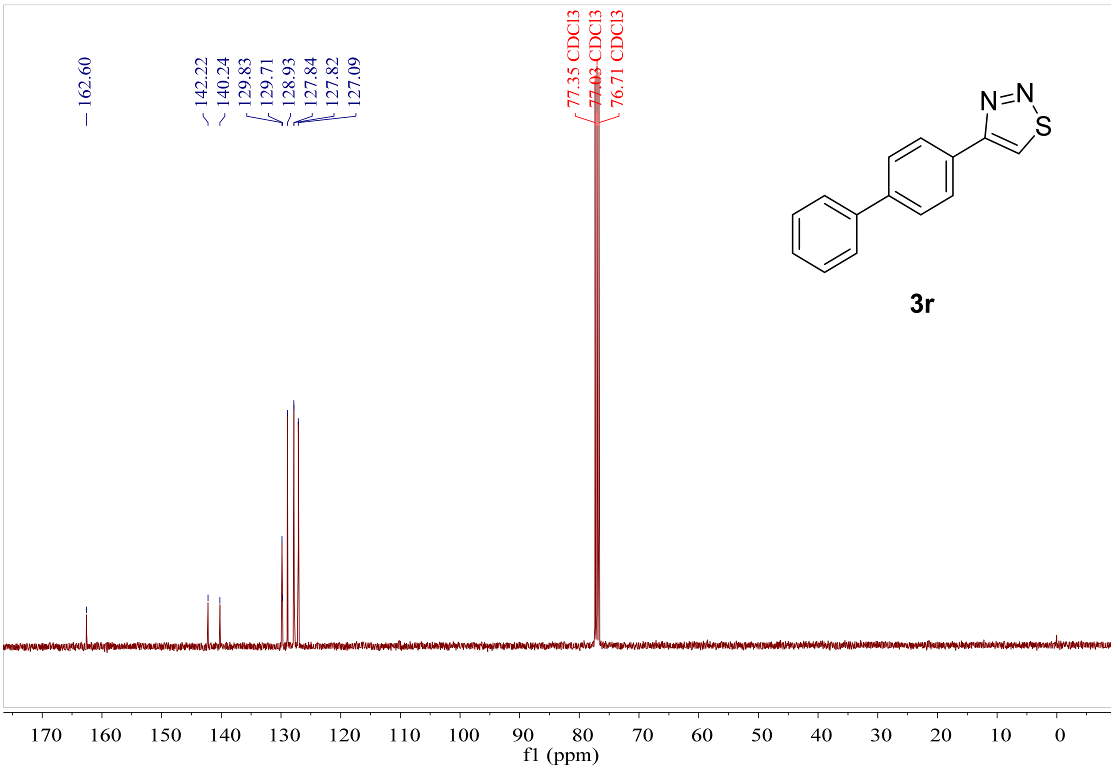
162.60
142.22
140.24
129.83
129.71
128.93
127.84
127.82
127.09
77.35 CDCl3
77.03 CDCl3
76.71 CDCl3
N=N
S
3r
f1 (ppm)

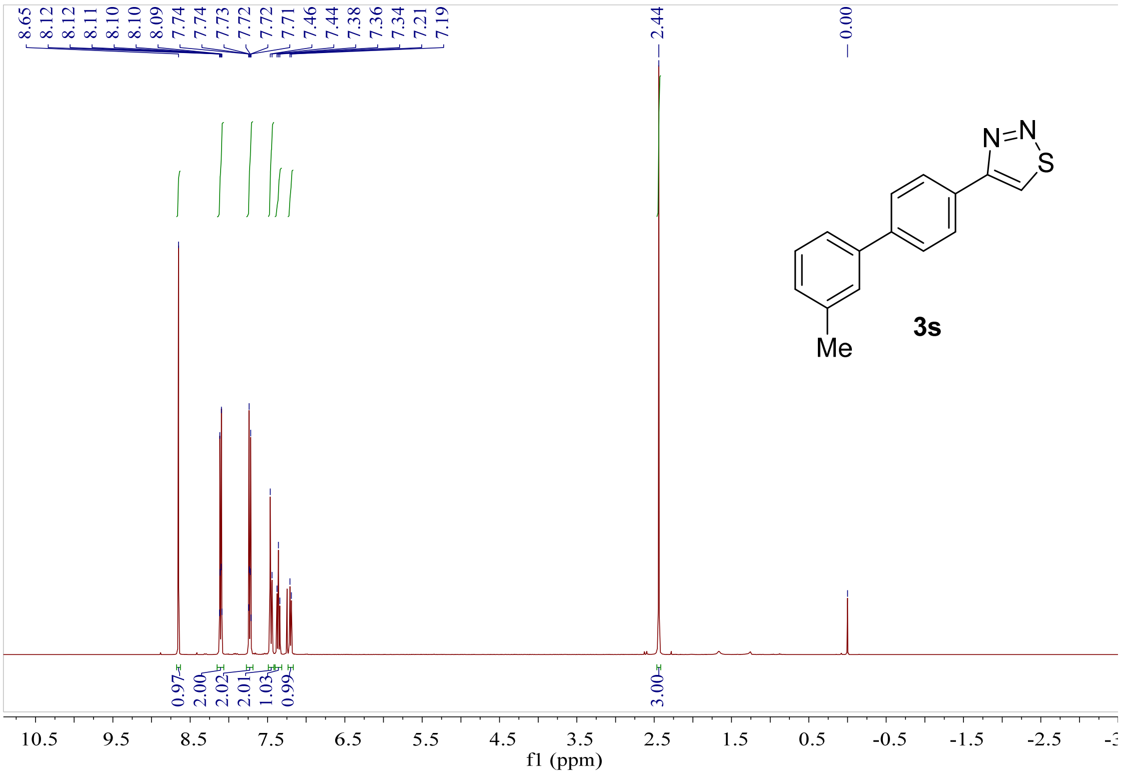
8.65
8.12
8.12
8.11
8.10
8.10
8.09
7.74
7.74
7.73
7.72
7.72
7.71
7.46
7.44
7.38
7.36
7.34
7.21
7.19
2.44
0.00
0.97
2.00
2.02
2.01
1.03
0.99
3.00
N=N
S
Me
3s
10.5 9.5 8.5 7.5 6.5 5.5 4.5 3.5 2.5 1.5 0.5 -0.5 -1.5 -2.5
f1 (ppm)

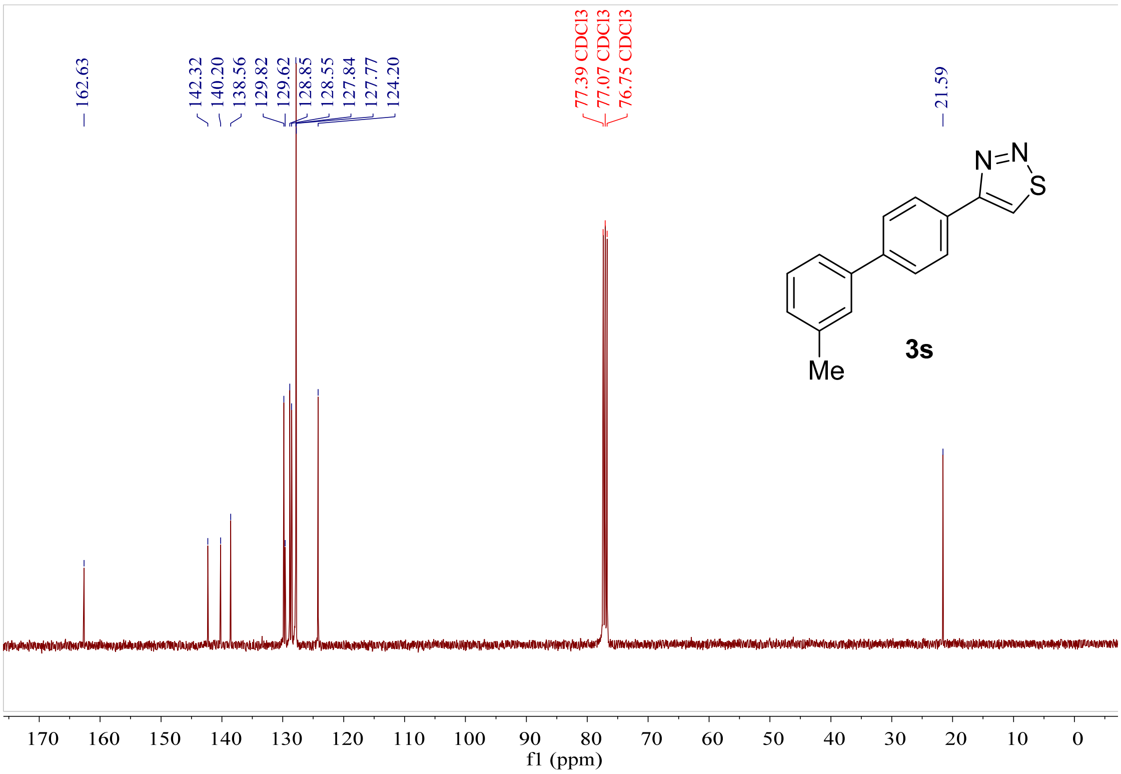
162.63
142.32
140.20
138.56
129.82
129.62
128.85
128.55
127.84
127.77
124.20
77.39 CDCl3
77.07 CDCl3
76.75 CDCl3
21.59
N=N
S
Me
3s
170 160 150 140 130 120 110 100 90 80 70 60 50 40 30 20 10 0
f1 (ppm)

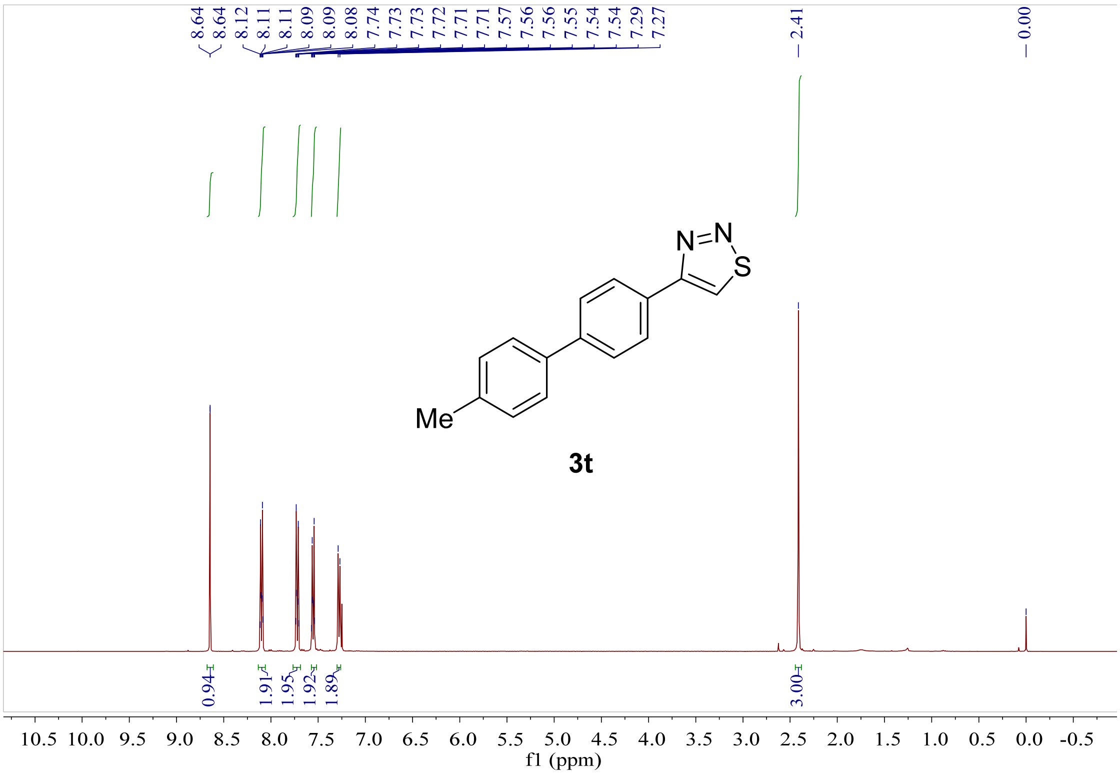
8.64
8.64
8.12
8.11
8.11
8.09
8.09
8.08
7.74
7.73
7.73
7.72
7.71
7.71
7.57
7.56
7.56
7.55
7.54
7.54
7.29
7.27
2.41
0.00
N=N
S
Me
3t
0.94
1.91
1.95
1.92
1.89
3.00
10.5 10.0 9.5 9.0 8.5 8.0 7.5 7.0 6.5 6.0 5.5 5.0 4.5 4.0 3.5 3.0 2.5 2.0 1.5 1.0 0.5 0.0 -0.5
f1 (ppm)

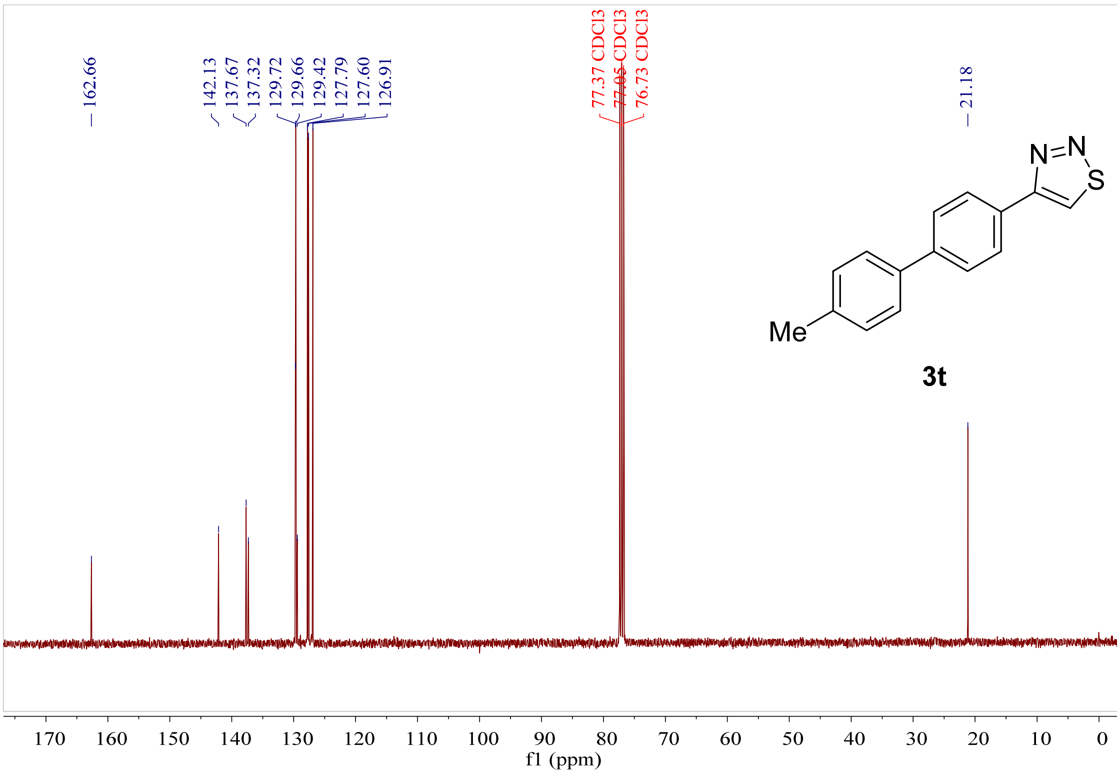
162.66
142.13
137.67
137.32
129.72
129.66
129.42
127.79
127.60
126.91
77.37 CDCl3
77.05 CDCl3
76.73 CDCl3
21.18
N=N
S
Me
3t
170 160 150 140 130 120 110 100 90 80 70 60 50 40 30 20 10 0
f1 (ppm)

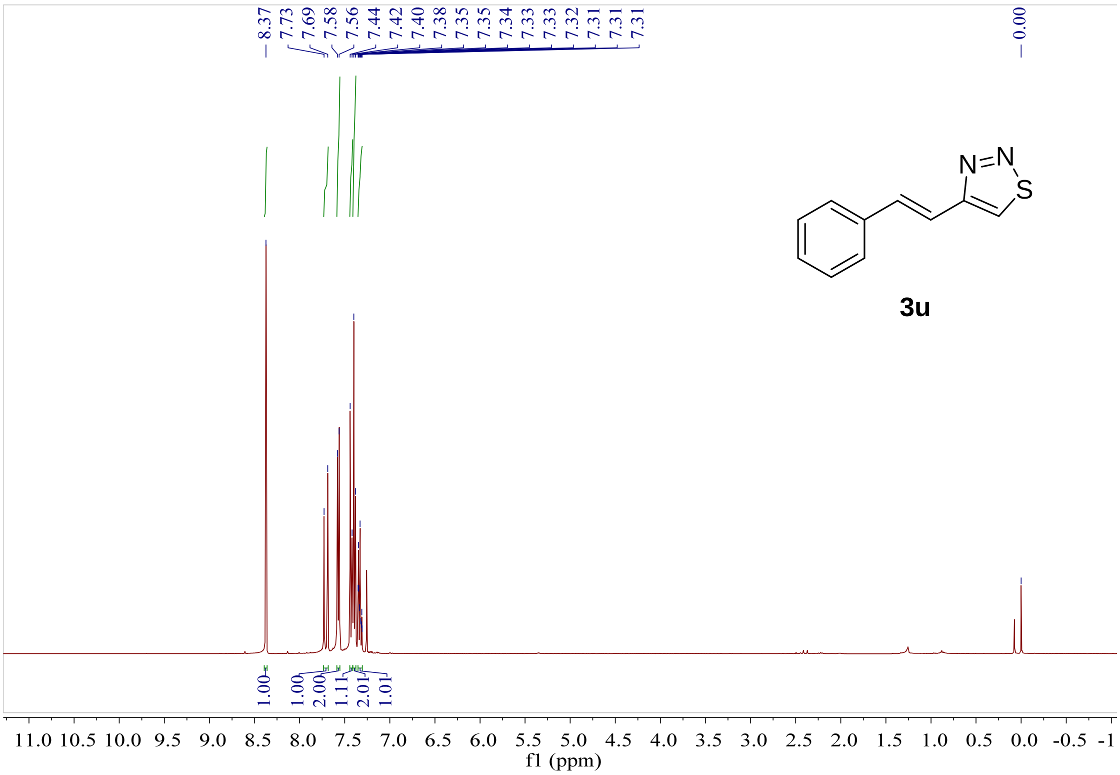
8.37
7.73
7.69
7.58
7.56
7.44
7.42
7.40
7.38
7.35
7.35
7.34
7.33
7.33
7.32
7.31
7.31
7.31
0.00
N=N
S
3u
1.00
1.00
2.00
1.11
2.01
1.01
11.0 10.5 10.0 9.5 9.0 8.5 8.0 7.5 7.0 6.5 6.0 5.5 5.0 4.5 4.0 3.5 3.0 2.5 2.0 1.5 1.0 0.5 0.0 -0.5 -1
f1 (ppm)

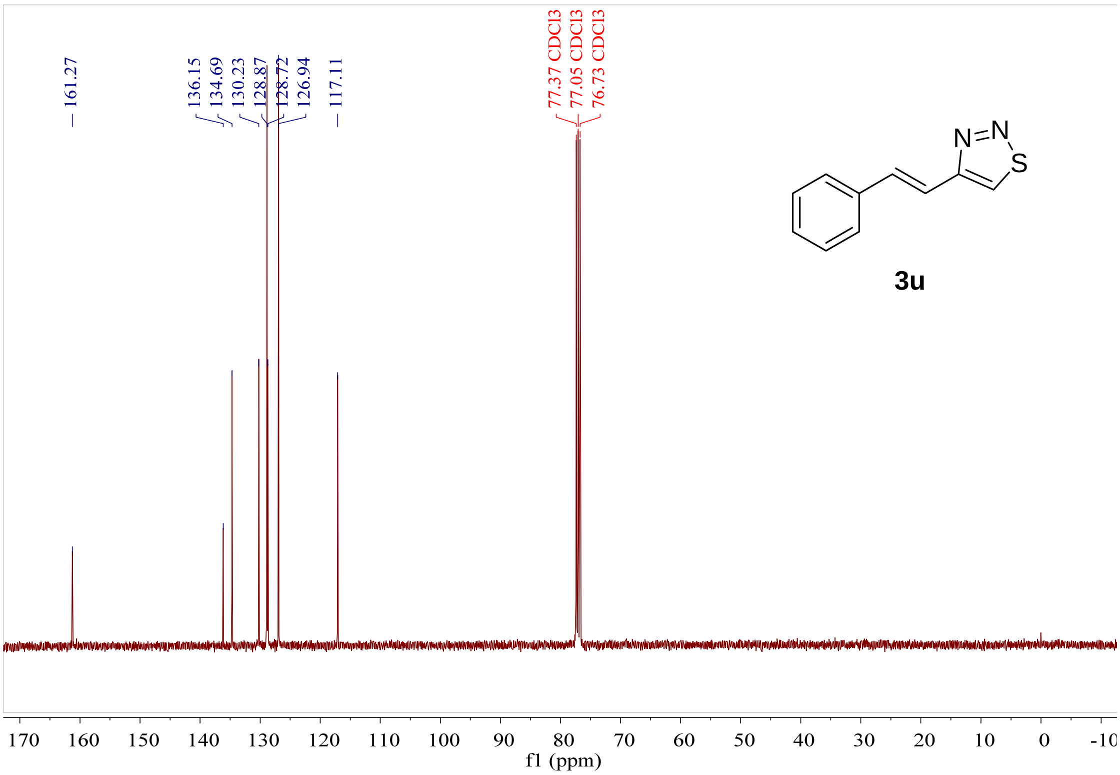
161.27
136.15
134.69
130.23
128.87
128.72
126.94
117.11
77.37 CDCl3
77.05 CDCl3
76.73 CDCl3
N=N
S
3u
170 160 150 140 130 120 110 100 90 80 70 60 50 40 30 20 10 0 -10
f1 (ppm)

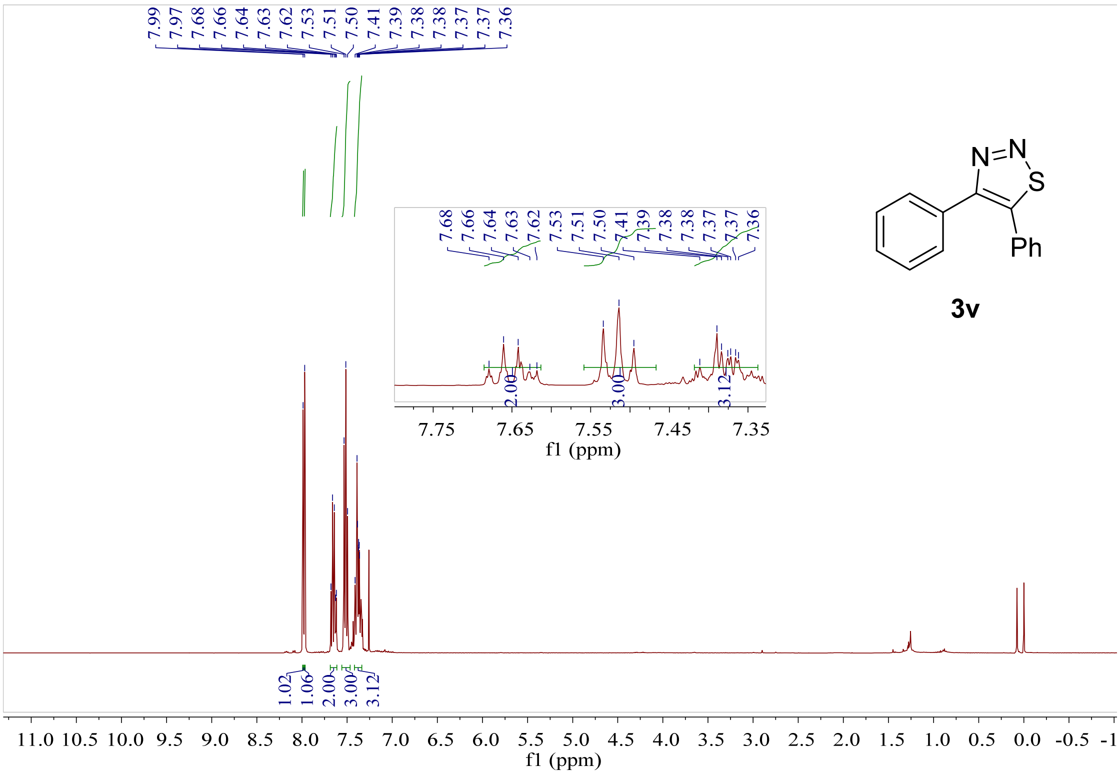
7.99
7.97
7.68
7.66
7.64
7.63
7.62
7.53
7.51
7.50
7.41
7.39
7.38
7.38
7.37
7.37
7.36
N=N
S
Ph
3v
1.02
1.06
2.00
3.00
3.12
7.75 7.65 7.55 7.45 7.35
f1 (ppm)
11.0 10.5 10.0 9.5 9.0 8.5 8.0 7.5 7.0 6.5 6.0 5.5 5.0 4.5 4.0 3.5 3.0 2.5 2.0 1.5 1.0 0.5 0.0 -0.5 -1
f1 (ppm)

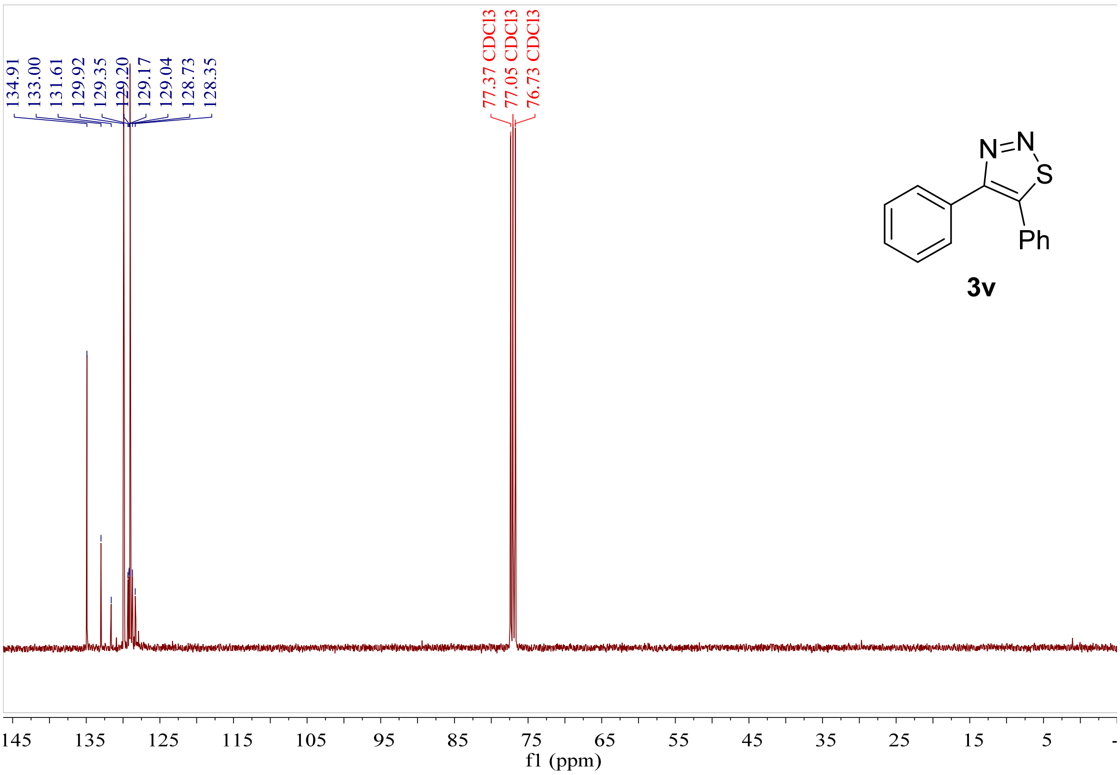
134.91
133.00
131.61
129.92
129.35
129.20
129.17
129.04
128.73
128.35
77.37 CDCl3
77.05 CDCl3
76.73 CDCl3
N=N
S
Ph
3v
145 135 125 115 105 95 85 75 65 55 45 35 25 15 5
f1 (ppm)

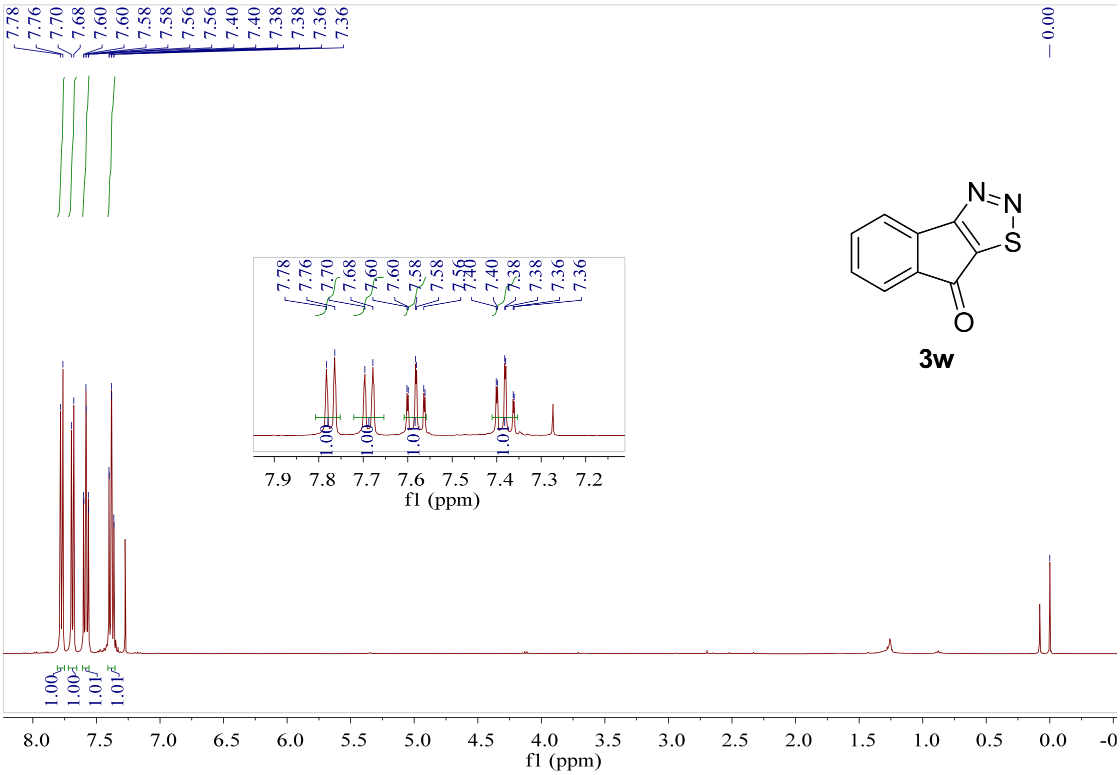
7.78
7.76
7.70
7.68
7.60
7.60
7.58
7.58
7.56
7.56
7.40
7.40
7.38
7.38
7.36
7.36
0.00
1.00
1.00
1.01
1.01
3w
N
N
S
O
7.9 7.8 7.7 7.6 7.5 7.4 7.3 7.2
f1 (ppm)
8.0 7.5 7.0 6.5 6.0 5.5 5.0 4.5 4.0 3.5 3.0 2.5 2.0 1.5 1.0 0.5 0.0
f1 (ppm)

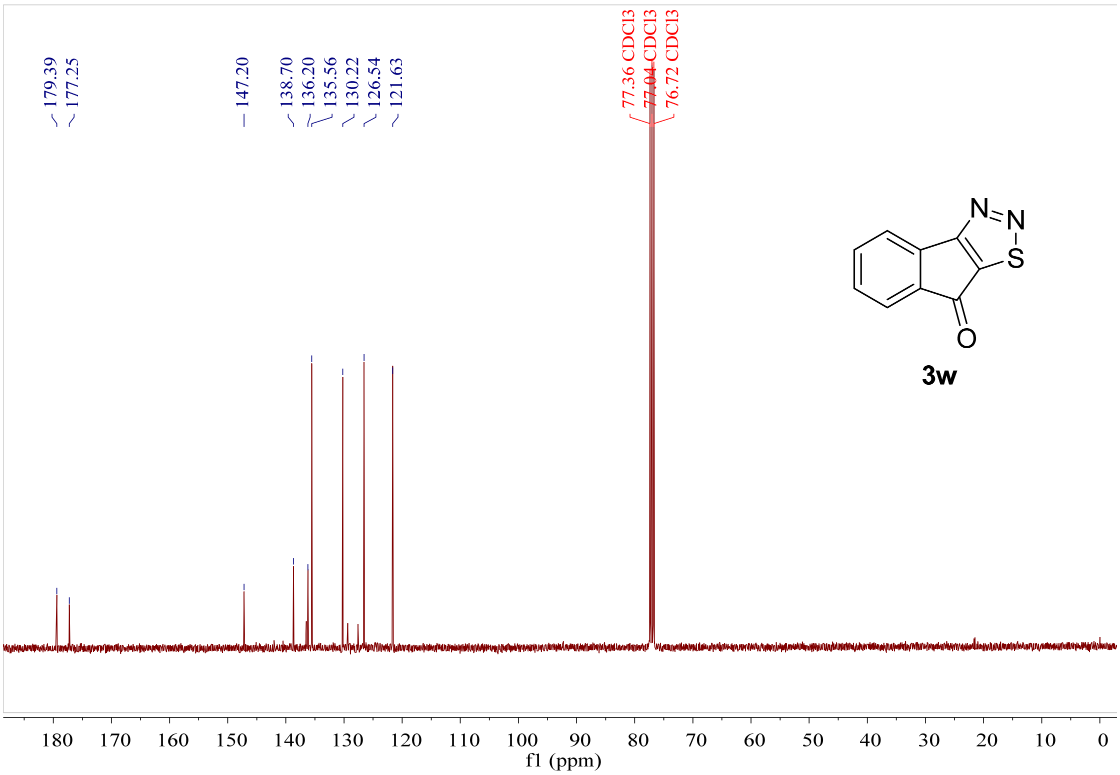
179.39
177.25
147.20
138.70
136.20
135.56
130.22
126.54
121.63
77.36 CDCl3
77.04 CDCl3
76.72 CDCl3
3w
N
N
S
O
180 170 160 150 140 130 120 110 100 90 80 70 60 50 40 30 20 10 0
f1 (ppm)

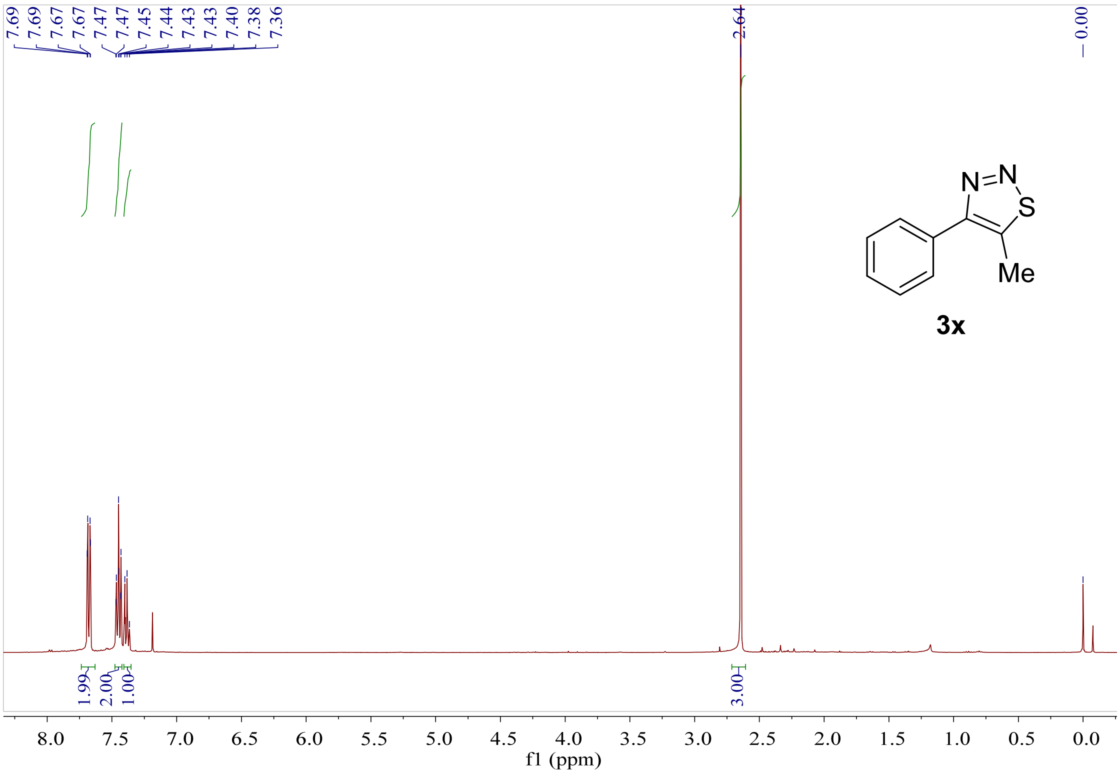
7.69
7.69
7.67
7.67
7.47
7.47
7.45
7.44
7.43
7.43
7.40
7.38
7.36
2.64
0.00
N=N
S
Me
3x
1.99
2.00
1.00
3.00
8.0 7.5 7.0 6.5 6.0 5.5 5.0 4.5 4.0 3.5 3.0 2.5 2.0 1.5 1.0 0.5 0.0
f1 (ppm)

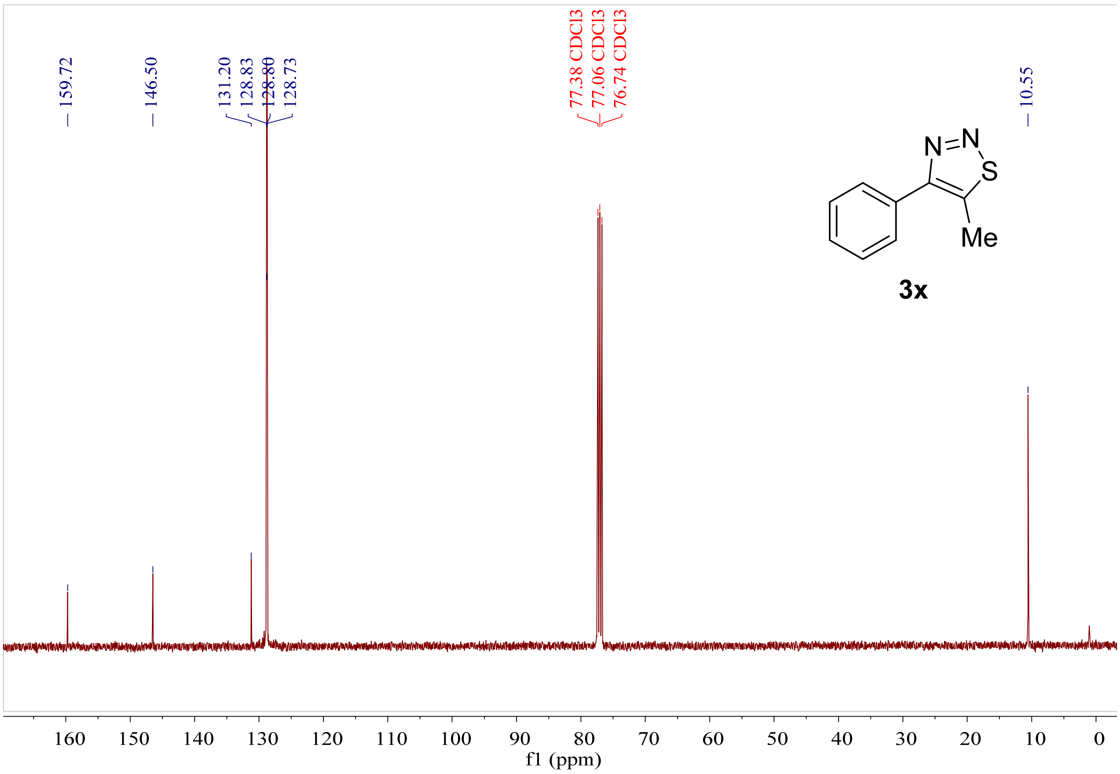
159.72
146.50
131.20
128.83
128.80
128.73
77.38 CDCl3
77.06 CDCl3
76.74 CDCl3
10.55
N=N
S
Me
3x
160 150 140 130 120 110 100 90 80 70 60 50 40 30 20 10 0
f1 (ppm)

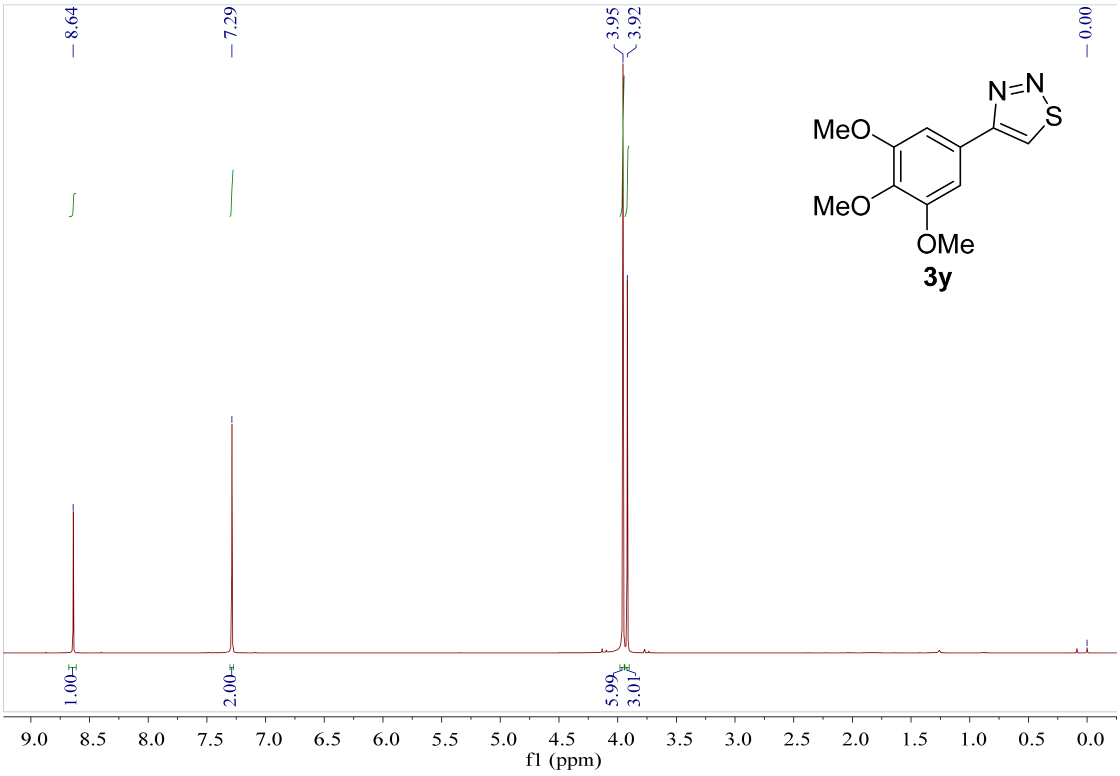
8.64
7.29
3.95
3.92
0.00
MeO
MeO
OMe
N=N
S
3y
1.00
2.00
5.99
3.01
9.0 8.5 8.0 7.5 7.0 6.5 6.0 5.5 5.0 4.5 4.0 3.5 3.0 2.5 2.0 1.5 1.0 0.5 0.0
f1 (ppm)

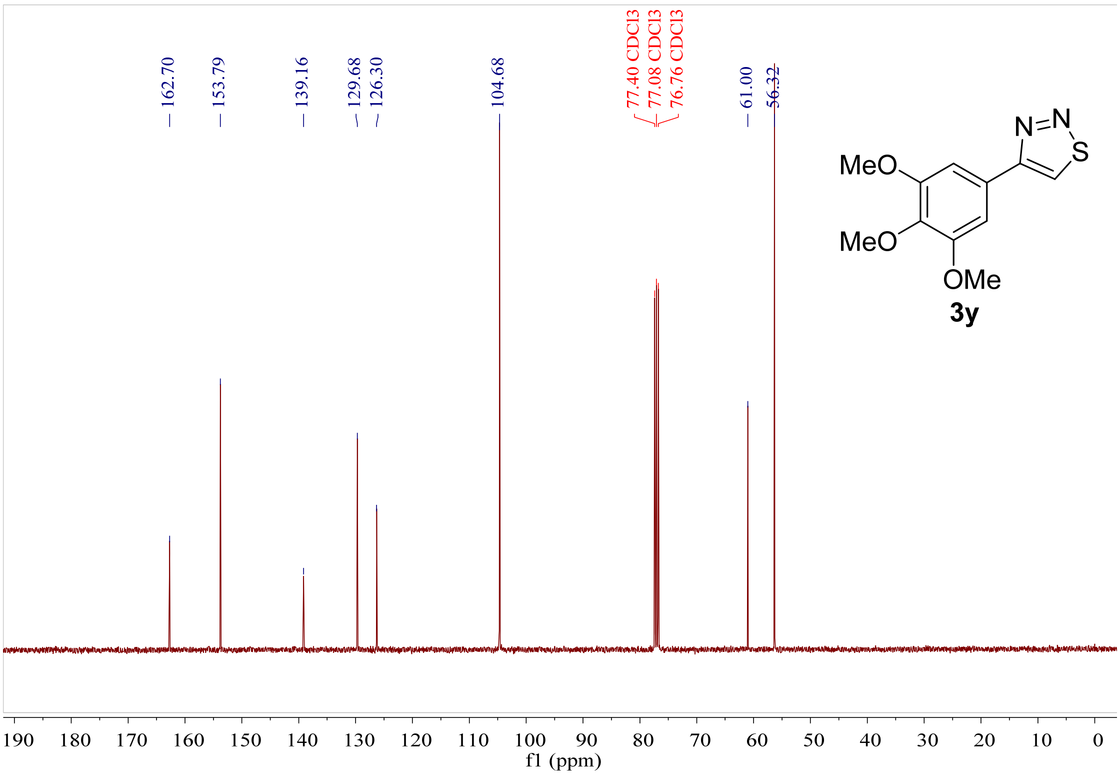
162.70
153.79
139.16
129.68
126.30
104.68
77.40 CDCl3
77.08 CDCl3
76.76 CDCl3
61.00
56.32
MeO
MeO
OMe
N=N
S
3y
190 180 170 160 150 140 130 120 110 100 90 80 70 60 50 40 30 20 10 0
f1 (ppm)

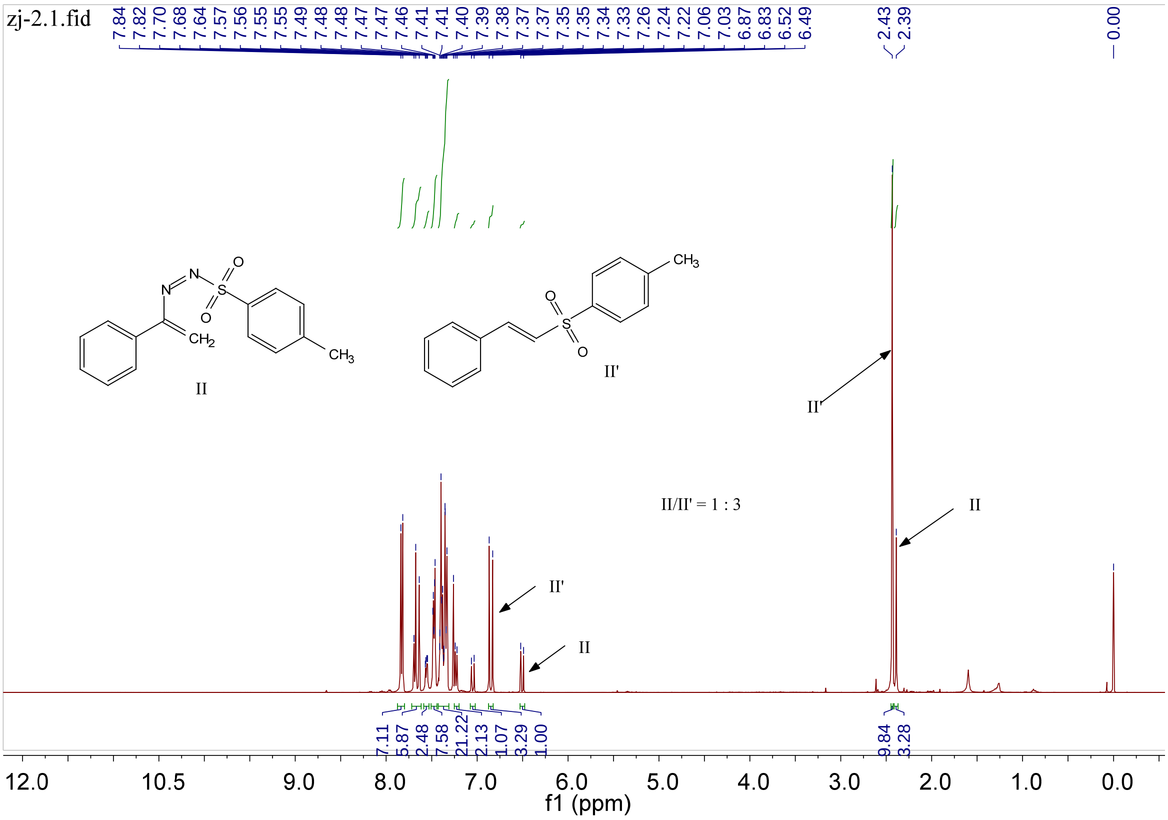
zj-2.1.fid
7.84 7.82 7.70 7.68 7.64 7.57 7.56 7.55 7.49 7.48 7.48 7.47 7.46 7.41 7.41 7.40 7.39 7.38 7.37 7.35 7.34 7.33 7.26 7.24 7.22 7.06 7.03 6.87 6.83 6.52 6.49
2.43 2.39
0.00
II
II'
II/II' = 1 : 3
7.11 5.87 2.48 7.58 21.22 2.13 1.07 3.29 1.00
9.84 3.28
12.0 10.5 9.0 8.0 7.0 6.0 5.0 4.0 3.0 2.0 1.0 0.0
f1 (ppm)
